# A structural exposé of noncanonical molecular reactivity within the protein tyrosine phosphatase WPD loop

**DOI:** 10.1038/s41467-022-29673-y

**Published:** 2022-04-25

**Authors:** Huanchen Wang, Lalith Perera, Nikolaus Jork, Guangning Zong, Andrew M. Riley, Barry V. L. Potter, Henning J. Jessen, Stephen B. Shears

**Affiliations:** 1 Signal Transduction Laboratory, National Institute of Environmental Health Sciences, National Institutes of Health, Research Triangle Park, NC 27709, USA; 2 Genome Integrity and Structural Biology Laboratory, National Institute of Environmental Health Sciences, National Institutes of Health, Research Triangle Park, NC 27709, USA; 3 Institute of Organic Chemistry, and CIBSS - the Center for Integrative Biological Signaling Studies, University of Freiburg, 79104 Freiburg, Germany; 4 Drug Discovery and Medicinal Chemistry, Department of Pharmacology, University of Oxford, Mansfield Road, Oxford OX1 3QT, UK

## Abstract

Structural snapshots of protein/ligand complexes are a prerequisite for gaining atomic level insight into enzymatic reaction mechanisms. An important group of enzymes has been deprived of this analytical privilege: members of the protein tyrosine phosphatase (PTP) superfamily with catalytic WPD-loops lacking the indispensable general-acid/base within a tryptophan-proline-aspartate/glutamate context. Here, we provide the ligand/enzyme crystal complexes for one such PTP outlier: *Arabidopsis thaliana* Plant and Fungi Atypical Dual Specificity Phosphatase 1 (A*t*PFA-DSP1), herein unveiled as a regioselective and efficient phosphatase towards inositol pyrophosphate (PP-InsP) signaling molecules. Although the WPD loop is missing its canonical tripeptide motif, this structural element contributes to catalysis by assisting PP-InsP delivery into the catalytic pocket, for a choreographed exchange with phosphate reaction product. Subsequently, an intramolecular proton donation by PP-InsP substrate is posited to substitute functionally for the absent aspartate/glutamate general-acid. Overall, we expand mechanistic insight into adaptability of the conserved PTP structural elements.

Considerable efforts continue to be made to understand the molecular basis of enzyme-catalyzed hydrolysis of phosphate esters and anhydrides^1^. A particular challenge for this objective is presented by the protein tyrosine phosphatase (PTP) family, in no small part because phosphotyrosine phosphatase activity is not the only function for this family of enzymes^2^. A significant number of PTPs dephosphorylate alternate substrates such as RNA, phosphatidylglycerophosphate, inositol phospholipids, and a specialized class of signaling molecules known as diphospho-*myo*-inositol polyphosphates (inositol pyrophosphates, or PP-InsPs; Fig. 1a, b)^2–8^. Despite the evolution of these catalytic differences, there has been a high degree of conservation of key structural features of the PTP active site (Supplementary Fig. 1)^2–5,9^. One of these prominent structural elements is a flexible loop named WPD after its three most highly conserved residues, which includes an Asp (or occasionally Glu) that is typically described as an indispensable proton-donor to the leaving group (Supplementary Figs. 1, 2a). This catalytic acid is inserted into the active site by the closure of the WPD loop. Much attention is being devoted to determining how differences in conformational dynamics of this loop can contribute to catalytic versatility within the PTP family^9,10^.

Nevertheless, there are examples of PTPs that show intriguing departures from the canonical aspects of the WPD loop. For example, the human *DUSP23* gene encodes a VH1-like member Z (VHZ) protein in which the WPD-loop catalytic-acid, Asp65, is subservient to a remote Glu134 fulfilling the primary general acid function^11^. The ability of substrate to enter the catalytic pocket in either of two binding modes allows Asp65 to substitute as the catalytic acid when Glu134 is mutated^11^. Other similar dual general acid PTPs have been identified (e.g., T*k*Ptp expressed by the hyperthermophilic archaeon *Thermococcus kodakaraensis* KOD1^12^). Furthermore, there are some important PTPs in which the WPD motif is entirely absent from the host loop (Supplementary Fig. 2a). These enzymes include human CDC25 phosphatase, which supervises cell-cycle checkpoints^13^, the phosphoinositide/protein phosphatase PTEN^14,15^, MCE1, an mRNA capping enzyme that is essential for mRNA processing^16^, Baculovirus RNA 5'-phosphatase^8^ and its human ortholog, DUSP11/PIR1^7^, which participates in innate immune responses to viral infection^17^, and Siw14, a PTP-type PP-InsP phosphatase in *Saccharomyces cerevisiae*^6^ (Supplementary Fig. 2a,b). There has been only limited speculation in the literature as to how PTP reactions might proceed in the complete absence of a classical WPD-loop catalytic acid^7,8,13,14,18,19^. Practical progress towards resolving this deeply puzzling situation requires high resolution structures of members of this particular group of enzymes (i.e., those described in Supplementary Fig. 2a) in complex with their natural substrate. To date, no published studies of these specific enzymes have yielded the necessary enzyme/substrate complexes. Our goal has been to bridge this significant gap in our mechanistic understanding.

In this work, we begin by adding a protein to the list of PTPs that lack the WPD motif (Supplementary Fig. 2a), i.e., an *Arabidopsis thaliana* ortholog of Siw14 that that is encoded by a gene at locus tag At1g05000^20^. Hereafter we refer to the protein by its alternate nomenclature: A*t*PFA-DSP1. We demonstrate that this PTP actively hydrolyzes PP-InsPs. This is a significant accomplishment in itself: little is known about the nature of phosphatases in plants that might act as signaling off-switches by hydrolyzing PP-InsPs^21,22^, even though these polyphosphates license molecular defenses against herbivorous larvae and necrotrophic pathogens^23^, and supervise homeostasis of the growth-limiting phosphate micronutrient^24^. Next, we describe the crystal structures of the catalytic core of A*t*PFA-DSP1 (Supplementary Table 1) in complex with PP-InsPs and also a fluorinated PP-InsP analog^25^ at resolutions of up to 1.7 Å. These structural complexes include a variety of pre-reactant-, reactant-, intermediate- and product-bound states. Analysis of these structures, in concert with descriptions of ligand specificity and kinetic properties, together provide a data-driven proposal for a PTP reaction cycle that does not utilize any amino-acid residue as a general acid. Instead, we posit that PP-InsP substrate itself provides a proton to the leaving phosphate group, via a water relay. Additionally, we conclude that the hydrolyzed phosphate remains trapped as an enzyme-product complex, until it can be released in a prisoner exchange with another PP-InsP molecule; the latter process is assisted by the WPD loop. Overall, our description of this reaction cycle dramatically expands understanding of the mechanistic significance of diversity in WPD loop chemistry, while not diverging from the overall context of conservation of structural elements. Our array of structural snapshots also represents a valuable resource for future practical and computational studies of non-canonical chemical barcodes within the WPD loop that tailor reactivity of individual PTP family members.

## Results and discussion

### A*t*PFA-DSP1 is an active and selective PP-InsP phosphatase

**Fig. 1:**
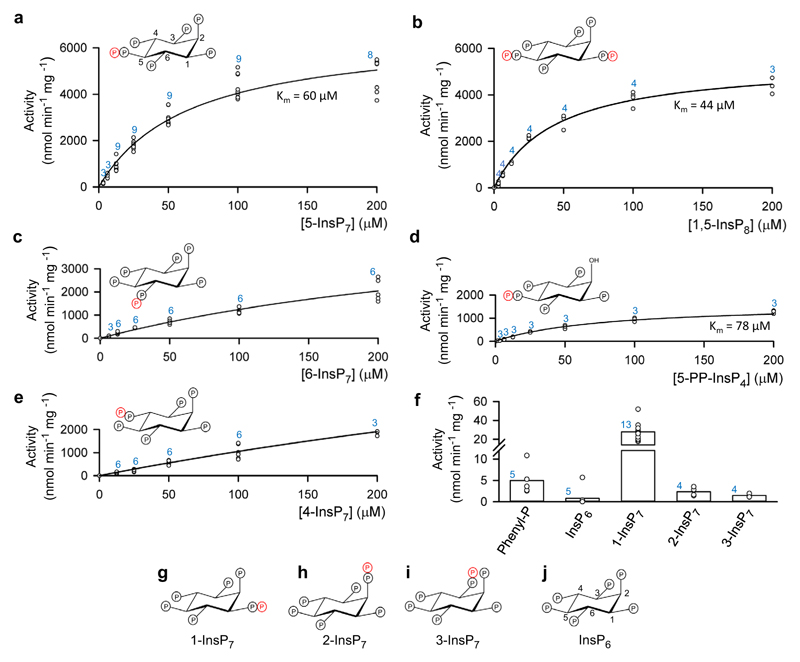
Structures of ligands used in this study and their rates of hydrolysis by A*t*PFA-DSP1. Michaelis–Menten kinetic plots are shown for the phosphatase activities of A*t*PFA-DSP1 towards: (**a**), 5-InsP_7_, (**b**), 1,5-InsP_8_, (**c**), 6-InsP_7_, (**d**), 5-PP-InsP_4_ and (**e**), 4-InsP_7_. Activity data (circles, some overlapping) are from each independent experiment at which the indicated substrate concentration was tested; the total number of such experiments is given above each data set in blue font. K_m_ values were calculated when statistically appropriate. The insets in panels (**a–e**) depict chair conformations of each substrate; the positions of each β-phosphate are emphasized in red. In panel (**f**), vertical bars represent mean values of activities against the weakest substrates when all were assayed at 10 μM concentrations. Activity data (circles, some overlapping) are from each independent experiment; the total number of such experiments is given above each data set in blue font. Phenylphosphate is abbreviated as Phenyl-P. Structures of the inositol phosphates are given as chair conformations in panels (**g**) (1-InsP_7_), (**h**) (2-InsP_7_, (**i**) (3-InsP_7_) and (**j**) (InsP_6_). Locants (using standard nomenclature for *myo*-inositol) are provided with the structures of 5-InsP_7_ and InsP_6_. Source data are provided as a Source Data file.

A previous study^18^, has demonstrated that A*t*PFA-DSP1 non-selectively hydrolyzes nucleotides and a variety of other phosphorylated substrates, although the authors acknowledged these molecules were unlikely to be physiologically relevant substrates. We confirmed slow rates of dephosphorylation for such molecules (at 10 μM; mean values (nmol min^−1^ mg^−1^) ± standard errors, followed by the number of independent experiments in parentheses; source data are provided as a Source Data file): ATP, 14 ± 2 (8); GTP, 13 ± 2 (9); phosphoribosyl pyrophosphate 15 ± 4 (5). Low activity towards phenyl phosphate 5.0 ± 1.5 (Fig. 1c) excludes protein phosphotyrosine as a natural substrate. Furthermore, we found up to two orders of magnitude higher rates of phosphatase activity against three naturally occurring PP-InsPs: 5-diphosphoinositol 1,2,3,4,6-pentakisphosphate (5-InsP_7_) (Fig. 1a), 1,5-bis-diphosphoinositol 2,3,4,6-tetrakisphosphate (1,5-InsP_8_) (Fig. 1b) and 6-diphosphoinositol 1,2,3,4,5-pentakisphosphate (6-InsP_7_) (Fig. 1c) Note that 6-InsP_7_ has only been definitively identified in *Dictyostelids*^26^, however, *Arabidopsis* and rice synthesize material identified as 6-InsP_7_ and/or (diphosphoinositol 1,2,3,5,6-pentakisphosphate (4-InsP_7_)^27^). We are not aware of a previous description of a standalone PP-InsP phosphatase expressed in any plant.

This enzyme also shows strong positional specificity. For example, the specificity constant (k_cat_/K_m_) for 5-diphosphoinositol 1,3,4,6-tetrakisphosphate (5-PP-InsP_4_), which has a 2-hydroxyl group, is approximately 6-fold lower than that for 5-InsP_7_ (see Fig. 1a, d), from which we conclude that the 2-phosphate of 5-InsP_7_ contributes to substrate recognition. Moreover, the enzyme has only weak activity against 4-InsP_7_ (Fig. 1e) and 1-diphosphoinositol 2,3,4,5,6-pentakisphosphate (1-InsP_7_) (Fig. 1f, g) and negligible activity towards either 2-diphosphoinositol 1,3,4,5,6-pentakisphosphate (2-InsP_7_), 3-diphosphoinositol 1,2,4,5,6-pentakisphosphate (3-InsP_7_) or inositol hexakisphosphate (InsP_6_) (Fig. 1f, h–j). Note that 2-InsP_7_ and 3-InsP_7_ have to date not been identified in any organism.

### Overall structure of A*t*PFA-DSP1

A low resolution (3.5 Å) structure of an A*t*PFA-DSP1 protein construct has previously been described, but significantly, without any candidate natural substrate in the crystal complex^18^. Consequently, the latter structure is not instructive for understanding reaction mechanisms. Nevertheless, it provided a template for molecular replacement to help us solve the structure of our fully traceable A*t*PFA-DSP1^49–215^ construct, to include several high-resolution crystal complexes (1.65–1.9 Å) (Supplementary Table 1, Supplementary Fig. 2b, c). There are two molecules in each asymmetric unit, although gel filtration analysis showed that the protein construct behaves as a monomer in solution (Supplementary Fig. 2d). The core structure of PFA-DSP1^49–215^ is similar to the equivalent region of the S*c*Siw14 ortholog (RMSD = 0.621 Å, derived by superimposing 798 comparable atoms; Supplementary Fig. 2e, f). Both structures exhibit a canonical PTP fold, in which their catalytic sites are defined by the three substrate-binding loops. The most N-terminal of these structural elements in A*t*PFA-DSP1 is equivalent to the WPD loop of canonical PTPs, although it does not contain the eponymous tripeptide motif (Supplementary Fig. 2a, f). Note that this loop makes a significant contribution to active site topology, as does the equivalent loop in canonical PTPs (Supplementary Fig. 2a, f). The C-terminus of the WPD loop in A*t*PFA-DSP1 retains a highly conserved Pro that in other PTPs is believed to act as a hinge to help dictate the range of motion of this flexible structural element^9^. Two additional substrate binding loops are described here as the P-loop and α5–α6 loop; these are equivalent to the P-loop and Q-loop of other Cys-based PTPs^3,4^. The amino-acid sequences of these catalytically important loop structures are extremely well conserved in other plant orthologs (Supplementary Fig. 2g).

**Fig. 2:**
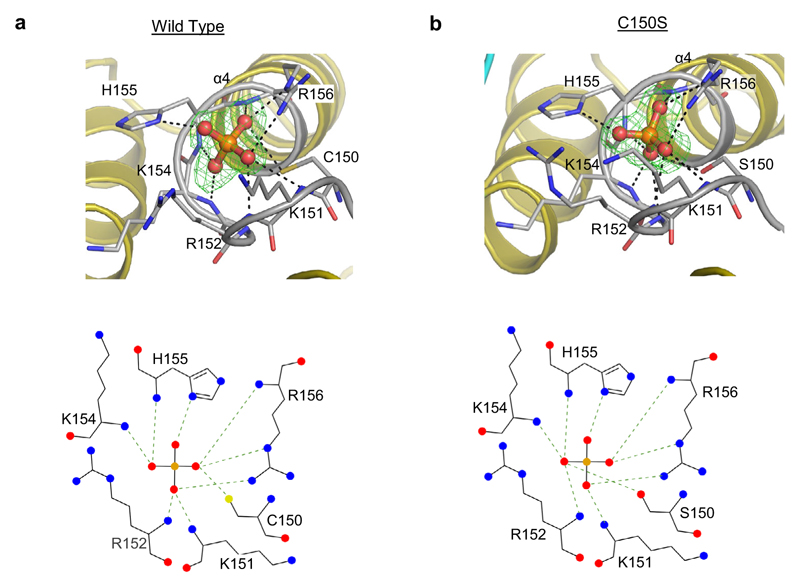
Description of the Pi(A) orientation associated with crystals of freshly purified A*t*PFA-DSP1^49–215^ and A*t*PFA-DSP1^49–150,C150S,151–215^. Panels (**a, b**), show the phosphate ion in stick and ball format (phosphorus is orange and oxygen in red) within a mixed stick- and ribbon-style rendition of the catalytic center of wild type and the C150S protein constructs, respectively (nitrogen is blue, sulfur is yellow). The omit Fo-Fc electron density maps, contoured at 5σ, are shown in green mesh; broken black lines show polar contacts. Corresponding ligand–protein interactions created by Ligplot+ are shown below each graphic. Source data are provided for (**a, b**) as PDB accession codes 7MOK and 7MOD, respectively.

These particular crystals of A*t*PFA-DSP1^49–215^ contain inorganic phosphate (Pi) in the catalytic center, proximal to the N-terminus of helix α4 (Fig. 2a). The oxygen atom that is positioned at the apex of the phosphate ion’s tetrahedral geometry points away from α4. This oxygen makes a hydrogen bond with the unprotonated N^δ1^ of His155 (we will return to this point below). We denote this orientation of Pi as pose A, i.e., Pi(A). The three other oxygen atoms of this Pi are intensively coordinated by amide groups of P-loop residues Lys151, Arg152 Lys154, His155, and Arg156. The side chains of Arg156 and Cys150/Ser150 also make polar contacts with Pi(A) (Fig. 2a, b). A previous structural analysis of the S*c*Siw14 orthologue found the active site occupied by a sulfate ion^19^ that is in an orientation equivalent to the Pi(A) pose (see below).

### Analysis of enzyme/substrate crystal complexes

A central goal of the current study was to prepare structural snapshots of enzyme/substrate crystal complexes for a PTP that does not utilize a canonical catalytic acid. Deriving such crystals for A*t*PFA-DSP1 was initially problematic; we were unable to displace enzyme-associated Pi by soaking crystals of wild type enzyme with 5-InsP_7_. This observation also speaks to how tightly the enzyme holds on to the Pi product (and see below). We therefore used a substrate-trapping strategy^3^ by mutating Cys150 to Ser, which reduced 5-InsP_7_ phosphatase activity 4000-fold (Table 1; Supplementary Fig. 3). Pi persisted in crystals of the freshly isolated A*t*PFA-DSP1^49–150,C150S,151–215^ mutant (Fig. 2b; Supplementary Fig. 4a), but it could now be successfully substituted by 5-InsP_7_ (Supplementary Table 1; Supplementary Fig. 4b), presumably because Pi is held less tightly than is the case with wild type protein.

**Fig. 3:**
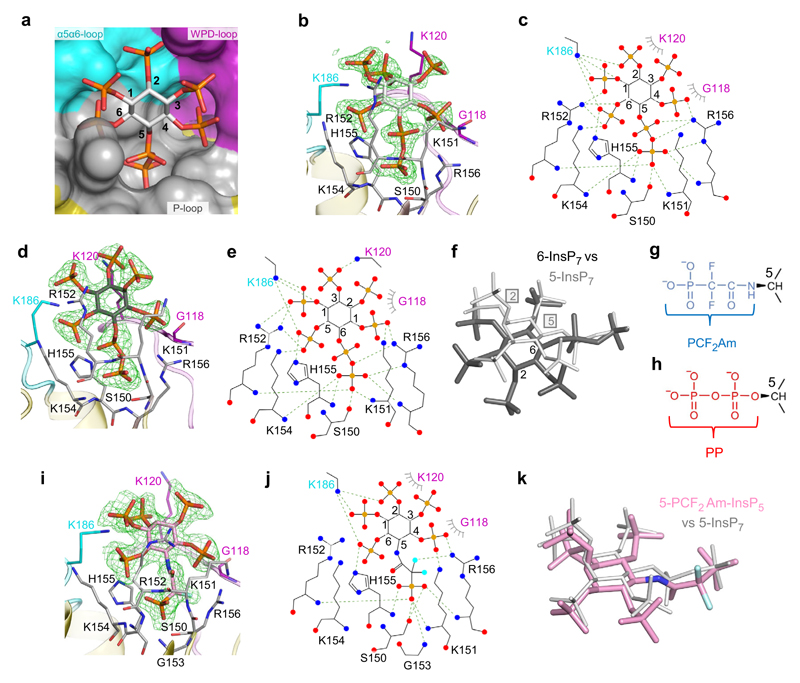
Binding of 5-InsP_7_, 6-InsP_7_ and 5-PCF_2_Am-InsP_5_ by A*t*PFA-DSP1^49–215^. **a** Surface representation colored to match structural elements correspond to gray for P-loop, cyan for α5-α6 loop, purple for WPD-loop, and yellow for the remainder. The 5-InsP_7_ is shown in stick format; carbon is white, phosphorus is orange and oxygen is red. Phosphate groups are numbered according to standard nomenclature. Panel (**b**) shows a similar orientation of 5-InsP_7_, with key interacting residues in stick format; nitrogen is blue, and oxygen. Panel (**c**) is a rendering of the ligand–protein interactions created by Ligplot+. Polar contacts within 3.2 Å are depicted with broken green lines. Hydrophobic interactions are shown in grey eyelash style. Equivalent data are shown in panels (**d, e**) for 6-InsP_7_ as the ligand; the latter’s carbons are colored dark gray. Panel (**f**) superimposes 6-InsP_7_ (dark gray; numbers denote positions of 2- and 6-phosphates) upon 5-InsP_7_ (light gray; boxed numbers denote 2- and 5-phosphates). Panels (**g, h**) compare the chemical structures of the α-phosphono-α,α-difluoroacetamide group (PCF_2_Am; blue) and the 5-diphosphate group (PP; red)respectively. Panels (**i, j**), show binding interactions for 5-PCF_2_Am-InsP_5_ (carbon is pink, and fluorine is cyan). Panel (**k**) superimposes 5-PCF_2_Am-InsP_5_ upon 5-InsP_7_ using the same color schemes as in panels (**i, f**) The omit Fo-Fc electron density maps, contoured at 5σ, are shown in green mesh. Source data files are provided as PDB accession codes 7MOE, 7MOF and 7MOG.

The 5-InsP_7_ substrate is held in an 11.6 Å wide and 15.2 Å deep pocket by polar interactions between the 1-, 4-, 5β-, and 6-phosphates and multiple residues in the P-loop: Lys151, Arg152, Lys154, His155 and Arg156 (Fig. 3a–c). These data also reveal that the His155 imidazole ring is flipped (180° *χ*^2^ angle changes) relative to its orientation in the Pi-bound crystal structures (Fig. 3b, c; Supplementary Fig. 4a, b). We analogize such behavior by depicting His155 as a Janus residue, such that the catalytic core is overlooked by either of the two faces of the imidazole ring, depending upon the nature of the ligand: in this case, 5-InsP_7_ (Fig. 3a–c) or Pi(A) (Fig. 2a, b). The protonated N^*ε*2^ in His155 forms a polar contact with the PP-InsP substrate, and the N^δ1^ forms a hydrogen bond with the hydroxyl group of Asp191, in which the carbonyl oxygen is further stabilized by Arg188 (Supplementary Fig. 4b). Support for the proposed catalytic significance of Asp191 and His155 emerged from mutagenic studies, which reduced activity by >90% (Table 1).

The 1- and 2-phosphates of 5-InsP_7_ also make multiple polar interactions with Lys186 in the α5-α6 loop (Fig. 3b, c). Gly118 and Lys120 from the WPD-loop make contributions to ligand binding with van der Waals interactions. Ser150, which replaces the catalytic cysteine, is located at the bottom of the ligand binding pocket and it helps to trap 5-InsP_7_ through its polar contact with one of the terminal oxygen atoms from the 5-β-phosphate moiety (Fig. 3a–c). We further noted that if a 1-β-phosphate were to be added to 5-InsP_7_, it would be solvent exposed, thereby rationalizing why 1,5-InsP_8_ is also actively hydrolyzed by this enzyme (Fig. 1b). Interestingly, catalytically productive binding of 1-InsP_7_, 2-InsP_7_ and 3-InsP_7_ is hindered by P-loop residues that would clash with either the axial 2-diphosphate or 2-monophosphate. This helps to explain why 1-InsP_7_, 2-InsP_7_ and 3-InsP_7_ are not efficient substrates for A*t*PFA-DSP1 (Fig. 1f).

Our observation that A*t*PFA-DSP1 exhibits similar catalytic activities towards both 5-InsP_7_ and 6-InsP_7_ (Fig. 1c) is rationalized by our structural data: due to the symmetry properties of the *myo*-inositol ring, the orientation of key, structural recognition features of 5-InsP_7_–particularly the diphosphate group and the flanking phosphate groups–are spatially mimicked by 6-InsP_7_ as a consequence of the latter being rotated in the active site relative to the position of 5-InsP_7_ (Fig. 3d–f). Thus, residues that interact with 5-InsP_7_ have an equivalent function with 6-InsP_7_. Interestingly, the WPD-loop makes a contribution to 6-InsP_7_ binding: Lys120 has polar contact with the 2-phosphate, while Gly118 has van der Waals interactions (Fig. 3d, e). *Dictyostelium discoideum* is known to synthesize 6-InsP_7_^26^; our accommodation of the latter into the active site of A*t*PFA-DSP1 may be a realistic structural model to rationalize putative 6-InsP_7_ phosphatase activity by a candidate D*d*PFA-DSP1 gene (DDB_G0285909). *Arabidopsis* and rice synthesize material identified as 6-InsP_7_ and/or 4-InsP_7_^27^.

There is considerable interest in the development of metabolically resistant PP-InsP bioisosteres that yield useful structural information for inhibitor development, and may also assist in deriving recalcitrant crystal complexes (see^28^). One recent development has been to replace the scissile 5-β-phosphate of 5-InsP_7_ with a phosphonodifluoromethyl (PCF_2_) group to yield 5-PCF_2_Am-InsP_5_ (Fig. 3g–i)^25^; for the purpose of synthetic strategy and analogue stability, the 5-α-phosphate group is replaced by an acetamide linkage. Interestingly, the addition of a PCF_2_ group to substrate analogs of certain protein tyrosine phosphatases can increase ligand affinity^29,30^. Furthermore, we^25^ have previously hypothesized that the binding affinity of 5-PCF_2_Am-InsP_5_ might be enhanced for ligand/protein interactions that do not involve Mg^2+^ (as is the case for the capture of PP-InsPs by A*t*PFA-DSP1). Indeed, we obtained a crystal complex of A*t*PFA-DSP1^49–150,Cys150Ser,151–215^ with 5-PCF_2_Am-InsP_5_ in the active site (Fig. 3i–k). The orientation of 5-PCF_2_Am-InsP_5_ is very similar to that of 5-InsP_7_, except that the 5-PCF_2_Am group makes a gain-of-function interaction between one fluorine atom and the guanidinium group of Arg156. Our data validate the value of 5-PCF_2_Am-InsP_5_ for the study of specific modes of PP-InsP/protein interactions.

**Fig. 4:**
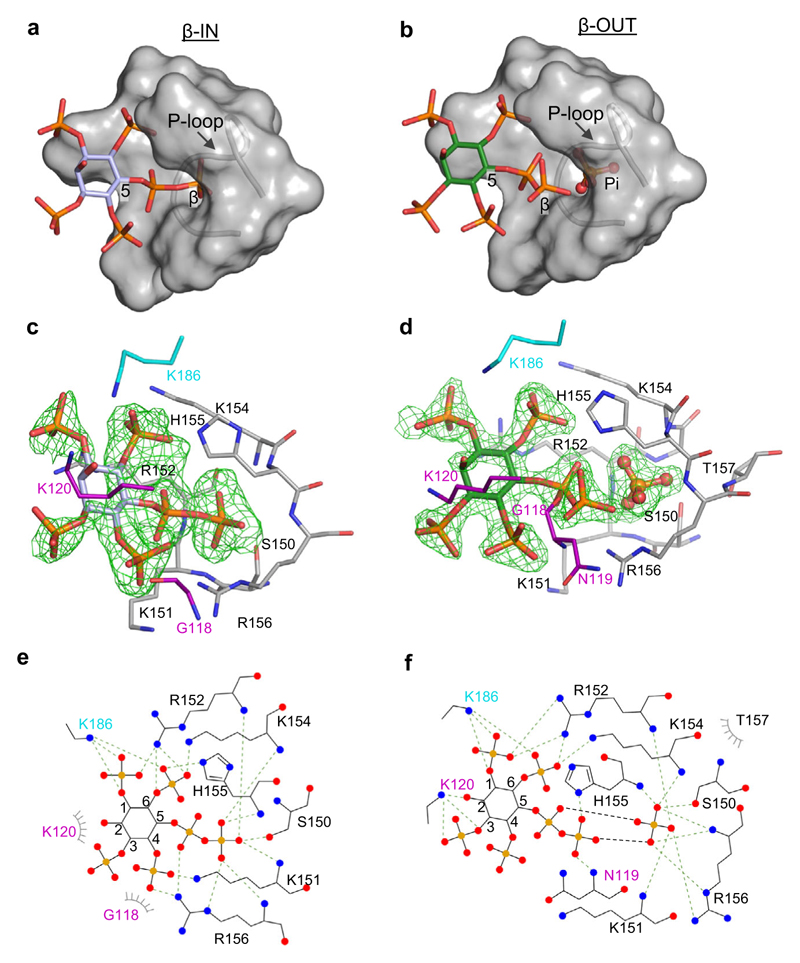
A*t*PFA-DSP1 binds 5-PP-InsP_4_ in two orientations, which are associated with either the presence or absence of Pi. Panels (**a, b**) are surface representations of the ligand binding pocket, with 5-PP-InsP_4_ drawn in stick format, in poses labeled β-IN (pale lavender carbons) and β-OUT (green carbons), respectively; phosphorus is orange and oxygens are red. Note the presence of Pi(B) (in stick and ball format) in panel (**b**). Panels (**c, d**) show the corresponding stick depictions of 5-PP-InsP_4_ and its interacting amino-acid residues (nitrogens are colored blue). The Fo-Fc electron density maps (green mesh) are contoured at 3σ in panel (**c**) and 5σ in panel (**d**). Panels (**e, f**) are renderings of the corresponding ligand–protein interactions created by Ligplot+. The source data file is provided as PDB accession code 7MOH.

We also obtained instructive crystals of A*t*PFA-DSP1 in complex with 5-PP-InsP_4_ (Fig. 4) in which the orientation of the ligand differed between the two asymmetric units. In one, 5-PP-InsP_4_ adopts a configuration that is very similar to that of 5-InsP_7_, with an extended α-β phosphoanhydride that projects its β-phosphate into the catalytic pocket (‘β-IN’ pose, Fig. 4a; Supplementary Fig. 4c–f). In the other asymmetric unit, the β-phosphate of 5-PP-InsP_4_ is diverted away from the catalytic pocket (‘β-OUT’ pose) at a near right angle or a *cis* conformation to the α-phosphate; the phosphorus atom of this β-phosphate is 4 Å displaced from its equivalent in the β-IN pose (Fig. 4a–f; Supplementary Fig. 4d, g, h). These conclusions are strengthened by the low values for the β-factors associated with the two alternate positions of the β-phosphate (Supplementary Fig. 4f, g). Remarkably, PP-InsP_4_ β-OUT substrate and Pi product are captured together in the same crystal complex (Fig. 4d, f; Supplementary Materials Fig. 4d). Bearing in mind that we could not derive crystals of the apoenzyme (i.e., either Pi and/or substrate is always present), we hypothesize that these crystal complexes describe two reaction states of a process that we analogize as a prisoner exchange: entry of PP-InsP substrate is tightly coupled to Pi exit. Interestingly, the WPD-loop appears to play a particularly significant role in delivering substrate to the exchange point: in the β-OUT configuration, Lys120 in this loop has three interactions with the substrate’s 2-hydroxyl and 3-phosphate groups, and Asn119 interacts with the 5-β-phosphate (Fig. 4d, f). All of these interactions are absent from the β-IN configuration (Fig. 4c, e). Also of interest in that the Janus residue His155 imidazole ring is flipped 180° in the β-OUT pose relative to its β-IN orientation (Supplementary Fig. 4e, h).

Another key observation to emerge from these experiments with A*t*PFA-DSP1^49–150,Cys150Ser,151–215^ is that in the crystal complex that contains 5-PP-InsP_4_ substrate and Pi, the latter’s oxygen atom at the apex of the tetrahedral structure points towards the α4 helix (Supplementary Fig. 4d, 5a). We name this orientation as Pi(B), to distinguish it from Pi(A), in which the apex of the tetrahedron points away from the α4 helix (Fig. 2). We are aware of only one other PTP structure that contains Pi in a pose equivalent to Pi(B): this protein is a mutant version of Wzb (the catalytic Cys is mutated to Ala; Supplementary Fig. 5b), a low molecular weight PTP produced by the prokaryote pathogen *Vibrio vulnificus*^31^. On the other hand, two crystal complexes, PTPMT1 (PDB: 3RGO^32^) and MTMR6 (PDB: 2YF0) contain a Pi(B)-like conformation of a sulfate ion, in which the oxygen atom at the apex of the tetrahedron points towards the α helix and forms two hydrogen bonds with S109 or T343, respectively (Supplementary Fig 5c, d). In contrast, a sulfate ion in a crystal complex with S*c*Siw14^19^ is in the pose equivalent to that for Pi(A) (Supplementary Fig 5e).

**Fig. 5:**
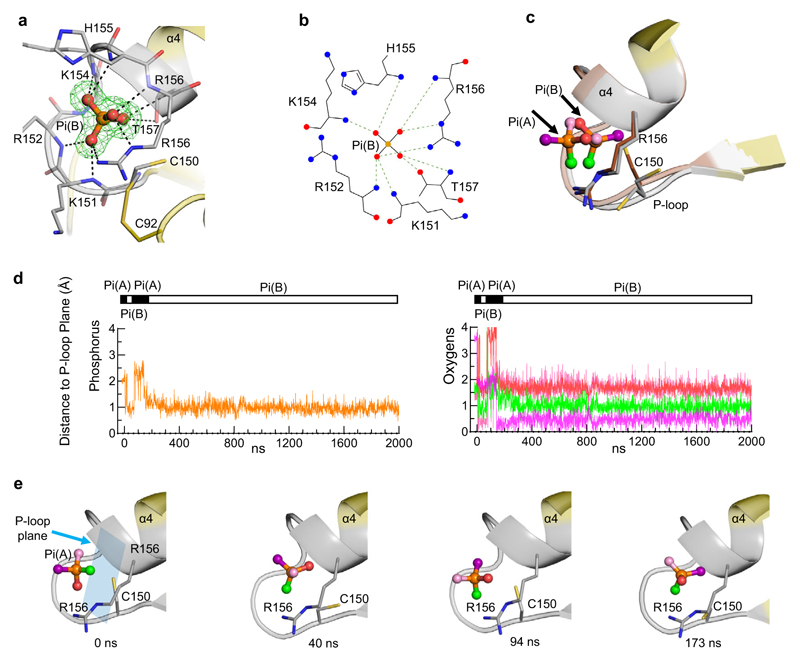
Analysis of Pi mobility inside the catalytic center. **a** Pi(B), in stick and ball format (phosphorus is orange, oxygen is red), in a crystal structure complex with A*t*PFA-DSP1^49–215^ (PDB accession code 7MOL) obtained in the absence of mercaptoethanol (see text). Broken lines depict polar interactions of Pi(B) (≤3.2 Å) with nearby residues (nitrogens are blue). The Fo-Fc electron density map (green mesh) is contoured at 5σ. **b** corresponding rendering of the ligand–protein interactions created by Ligplot+. **c** Pi(B) from panel (**a**) is superimposed upon Pi(A) (taken from Fig. 2a); the P-loops of the corresponding proteins are colored gray for Pi(B) and light brown/dark brown for Pi(A). Note the two conformations of Cys150, one of which may form a disulfide bond (see panel **a**). **d, e** the movement of the phosphorus atom is indicated by the orange trace, and each of the oxygen atoms of Pi(A) were arbitrarily colored either purple, light pink, red or green; this color scheme illustrates the predicted movements of each atom during molecular dynamics simulations, with reference to the plane of the backbone residues of the P-loop (blue rectangle; residues 151–156). Horizontal bars illustrate the switching between Pi poses A (black) and B (white). The coloration in this simulation has been transferred to Pi(B) in panels (**c**) and (**e**). Three additional replicates of the data in panel (**d**) are provided in Supplementary Fig. S8, and the initial 1000 ns of each of these aspects of all four simulations are animated in Supplementary Movies 1, 2, 3 and 4. Source data are provided as a Source Data file.

We also observed the Pi(B) pose in crystals of wild type enzyme prepared in the absence of β-mercaptoethanol (Fig. 5a, b). In this Pi(B) pose, the side chain of Cys150 may adopt alternate conformations, one of which may permit an intramolecular disulfide bond with Cys92 (Fig. 5a, c). Another distinguishing feature of Pi(B) is that it makes an interaction with the backbone of His155 (Figs. 4f; 5a, b), whereas Pi(A) makes polar contact with the His155 side chain (Fig. 2). Furthermore, we noted that Pi(B) can make polar contacts with both the side chain and amide backbone of Thr157 (Fig. 5a, b) whereas Pi(A) does not interact with this residue (Fig. 2). The sidechain of Thr157 could also contribute to catalysis if it were to assist in lowering the pKa of Cys150 (see below). Evidence that a Thr157 is catalytically important was derived from analysis of a Thr157Ala mutant, which reduced enzyme activity >97% (Table 1).

### Pi rotation within the catalytic pocket

Our capture of two alternate static poses of Pi within the catalytic site (Fig. 2; Fig. 5a–c) prompted us to consider the possibility of there being dynamic interchange between them. To interrogate this idea, four independent 2μs molecular dynamics simulations of A*t*PFA-DSP1^49–215^ were initiated with di-anionic Pi(A) in the catalytic pocket (i.e., structure 7MOK; Supplementary Table 1). Throughout each of these simulations, the root mean squared deviations of the heavy backbone atoms settled around 2 Å (Supplementary Fig 6a), indicative of a relatively stable overall protein configuration. From the root mean square fluctuations for Cα of the individual amino acids (Supplementary Fig 6b) it is concluded that the P-loop is a particularly stable entity. Moreover, dynamic cross correlation maps indicate no significant correlation between the movements of the P-loop and the considerably more dynamic WPD loop (Supplementary Fig 7). Thus, we used the relatively stable plane of P-loop backbone residues 151 to 156 as a reference point for monitoring the dynamics of the Pi ligand. Remarkably, these simulations show reorientations of Pi between two conformations that closely resembled those of Pi(A) and Pi(B) (Fig. 5d, e; Supplementary Fig. 8; Supplementary Movies 1–4), each of which has ionic interactions with P-loop residues (Supplementary Fig. 9). An analysis of the positional distribution of the phosphorus atom is consistent with a binary distribution of Pi between configurations A and B in the ratio 1:4 (Supplementary Fig. 10). Moreover, the one oxygen atom in Pi that is the most distant from the P-loop plane (as in Pi(A)) is only observed during 20% of the simulation time (Supplementary Fig. 10). The observed preference for the Pi(B) conformation found in our simulations is consistent with its stronger interaction free energy value (−81.6 ± 2.2 kcal/mol) compared to pose A (−68.1 ± 1.4 kcal/mol). Finally, in pose B, the phosphorus atom is on average 1.1Å closer to the P-loop plane compared to its position in pose A, which compares well with the difference of 1.5Å that was determined experimentally (Fig. 5c).

### Contending with the conundrum of PTP-catalyzed substrate hydrolysis in the absence of an Asp/Glu catalytic acid

In canonical PTPs the WPD motif includes a highly conserved general acid—an Asp (or occasionally Glu)—that is typically considered to be an indispensable proton-donor to the leaving group (Supplementary Fig. 1). This motif is absent from A*t*PFA-DSP1, other plant orthologs and S*c*Siw14 (Supplementary Fig. 2a, f, g). We have not identified another candidate Asp/Glu from outside the WPD loop. Furthermore, the proximity of Arg156 to the highly negatively charged PP-InsP substrate should oppose any dramatic reduction in the value of its guanidinium pKa that would be required for it to act as a general acid^33^. Also, there is no precedent in the PTP field for an Arg to perform a catalytic acid function^33^.

**Fig. 6:**
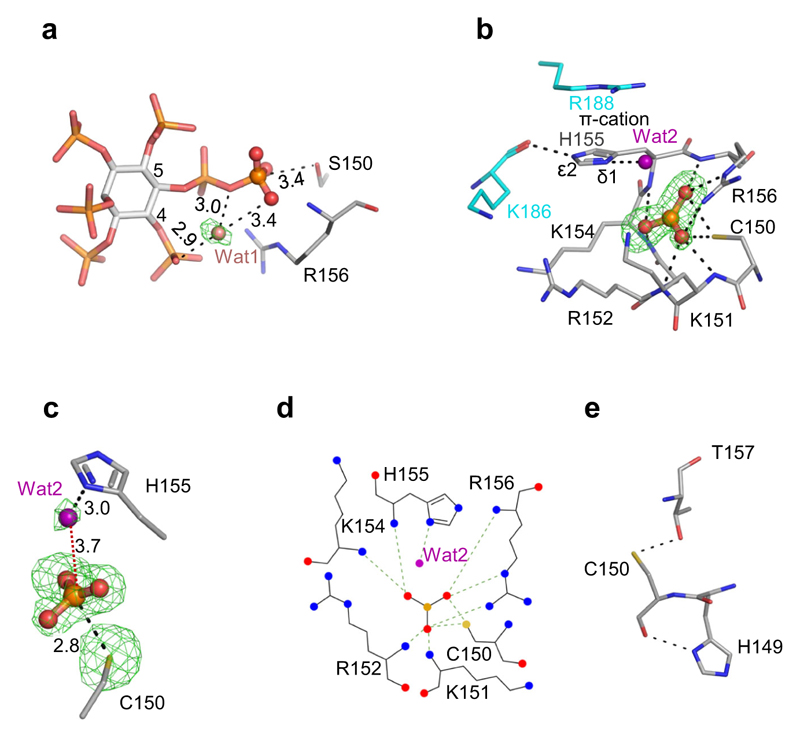
The formation of a metaphosphate-like reaction intermediate in A*t*PFA-DSP1^49–215^. **a** the relative positions of a catalytic water (Wat1; pink sphere) and 5-InsP_7_ (stick format) in a crystal complex with *AtPFA-DSP1*^49-150,C150S,151-215^ (PDB accession code 7MOD); polar interactions are highlighted with broken black lines, with bond distances marked in Å. The omit Fo-Fc electron density map is contoured at 5σ and shown in green mesh. **b** a reaction intermediate that we designate to be a metaphosphate anion (ball and stick format; orange for phosphorus, red for oxygens). The crystal complex depicted in this panel was obtained by soaking 0.1 mM 5-InsP_7_ into A*t*PFA-DSP1^49-215^ at pH 8.0 for 2 h (7MOM; Supplementary Table 1). Broken black lines designate polar interactions with residues (stick format; blue for nitrogen, yellow for sulfur). The omit Fo-Fc electron density map is contoured at 4σ and shown in green mesh. A putative reactive water molecule (Wat2) is shown as a purple sphere. Distances are described in Å. His155 is shown in N^*ε*2^-protonated *τ* tautomer state. **c** relative positions of Wat2 and the putative metaphosphate to illustrate distances between elements (Å); those within polar interaction distance are depicted as broken gray lines. The omit Fo-Fc electron density map is contoured at 4σ and shown in green mesh. Panel (**d**) is a rendering of the corresponding ligand–protein interactions created by Ligplot+. Panel (**e**) shows polar interactions (<3.2 Å; broken black lines) that Cys150 has with Thr157 and His149.

**Fig. 7:**
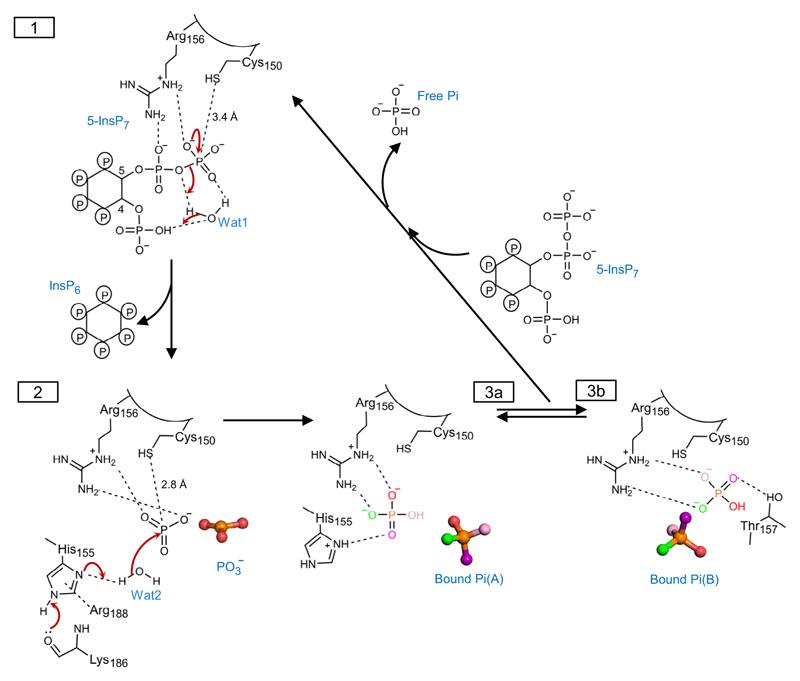
Proposed relationship between structural snapshots for A*t*PFA-DSP1 in the context of 5-InsP_7_ hydrolysis. Key polar contacts are highlighted with black dashed lines. Coloring of oxygen atoms is only for illustrative purposes. State **1** depicts presumed canonical nucleophilic attack by Cys150 on the β-phosphate of enzyme-bound 5-InsP_7_. We presume a thiophosphate intermediate is formed, although we did not capture one in our crystals. Also shown is a water molecule (Wat1) that we postulate to shuttle a proton from the substrate’s 4-phosphate to the diphosphate’s bridging oxygen, with the release of InsP_6_ product. State **2** depicts the proposed activation by His155 of a second reactive water (Wat2) to facilitate its capture by an enzyme-stabilized metaphosphate intermediate. The resulting enzyme-bound Pi, rotating between its ‘A’ and ‘B’ orientations, are depicted in States **3a** and **3b**. Despite this limited mobility, Pi remains trapped within the active site until a prisoner exchange with fresh substrate, so that a new catalytic cycle can begin. Some cautionary notes: all protonation states are illustrative and not definitive (nevertheless, there is consensus that the phosphate group that is targeted for hydrolysis by PTPs is di-anionic^9^), and it has previously been argued a metaphosphate intermediate cannot exist^38^ (see main text for further discussion).

It is therefore significant that our multiple PP-InsP/enzyme structures include a spatially conserved water molecule, Wat1 (Fig. 6a; Supplementary Fig. 11a, b), that forms a polar contact with the β-phosphate, a hydrogen bond with the diphosphate’s bridging oxygen atom, and a polar contact with an adjacent monophosphate group. Interestingly, Wat1 is not observed in a crystal complex with phenyl phosphate (Supplementary Fig. 11c), which is not an efficient substrate (Fig. 1c), even though the latter’s phosphate group is almost superimposable upon the β-phosphate of 5-InsP_7_ (Supplementary Fig. 11d). Thus, we posit that PP-InsPs recruit Wat1 to assist their own hydrolysis: for 5-InsP_7_ as substrate, we propose that a proton from the C-4 phosphate is relayed by Wat1 to the bridging oxygen atom of the diphosphate on the neighboring C-5, thereby stabilizing the phosphate leaving group (Fig. 7). This enzymatic requirement for a water molecule to shuttle a proton from a monophosphate to a diphosphate could make a significant contribution to catalytic regiospecificity while reducing the substrate’s intramolecular steric and electrostatic interference. This is a striking example of enzymatic adaptation to the unique physicochemical properties of the PP-InsP family.

Furthermore, we superimposed the A*t*PFA-DSP1-bound 5-InsP_7_ upon the tyrosine from an Asp-Ala-Asp-Glu-Tyr-Leu-vanadate substrate analog/PTP1B crystal complex^34^; this Tyr overlays the 5- and 6-phosphates of 5-InsP_7_ (Supplementary Fig. 11e). Thus, some basic structural elements of A*t*PFA-DSP1 and PTP1B are well conserved geometrically, despite the huge physicochemical differences in the nature of their respective substrates. Nevertheless, these data lead to an important conclusion that it would be electrostatically and sterically incompatible for A*t*PFA-DSP1 to have a general acid that is spatially equivalent to Asp181 in PTP1B, as it would clash with the negatively charged PP-InsP substrate (Supplementary Fig. 11e). An additional viewpoint (building on the proposed proton relay discussed above) is that the 4-phosphate of 5-InsP_7_ functionally replaces the catalytic Asp residue.

### Identification of a metaphosphate-like reaction intermediate in the A*t*PFA-DSP1 reaction cycle

In experiments in which crystals of wild type A*t*PFA-DSP1^49–215^ were soaked with reduced concentrations of 5-InsP_7_ at pH 8.0, we observed a near-planar, triangular-shaped electron density consistent with a metaphosphate; all three of its presumed P–O bonds are an appropriate 1.5 Å in length (Fig. 6b–d). There was no evidence of a pentacovalent phosphorane that would have signified an associative reaction pathway^35^. The putative metaphosphate intermediate in wild type enzyme is stabilized by intensive polar contacts with a cluster of residues in the P-loop, Lys151, Arg152, Lys154, His155, and Arg156, including the latter’s positively charged guanidinium group (Fig. 6b–d; Fig. 7).

Computational studies with PTPs support the idea that a metaphosphate-like species occurs during the formation and subsequent hydrolysis of the canonical PTP Cys-thiophosphate intermediate^9,36^. If formation of the latter is also a characteristic of A*t*PFA-DSP1, the pKa of Cys would need to be significantly depressed^37^. Other PTPs accomplish this through a network of hydrogen bonds that the catalytic Cys has with residues in the P-loop and the contiguous α4 helix^37^. Thr157 and His149 could potentially contribute to such a phenomenon in A*t*PFA-DSP1 (Fig. 6e), although we have not observed a thiophosphate intermediate.

We acknowledge authoritative reviews of the phosphoryl transfer and phosphatase literature which discount the possibility that a metaphosphate can accumulate as a reaction intermediate^1,38^. Nevertheless, there are descriptions of a metaphosphate as an intermediate in the reaction cycle of fructose-1,6-bisphosphatase^39,40^ and an evolutionarily related vanadium chloroperoxidase^41^ which, to our knowledge, have never been disproved experimentally. On the other hand, a previous description of a pentacoordinate phosphorane intermediate in crystals of β-phosphoglucomutase^42^ is now recognized to be a case of mistaken identity: the species in question turned out to be MgF_3_^−^, which was contributed by the crystallization buffer43. Both Mg^2+^ and F^−^ are present in our crystallization buffers. Therefore, we modeled 2mFo-Fc difference maps contoured at 1.5 σ; this analysis revealed that the longer Mg-F bond lengths of 1.8 Å extend beyond the center of the electron density and approach its boundary (Supplementary Fig 12a); further modeling with Fo-Fc difference maps shows that negative density features are especially prominent for two of the three fluoride atoms (Supplementary Fig 12b).

We also detected a metaphosphate-like molecule in a crystal complex obtained upon soaking 5-InsP_7_ into A*t*PFA-DSP1^49–150,Cys150Ser,151–215^ at pH 10 for one day (Supplementary Figs. 12c, d, 13). Bearing in mind uncertainty over side chain pKa values in a protein microenvironment^44^, we posit that a finite degree of reactivity is licensed by an extended time of incubation of the protein at very alkaline pH, along with the proximity of Ser150 to the β-phosphorus atom of 5-InsP_7_ (Fig. 6a; Supplementary Fig. 13). The modeled Fo-Fc electron density difference for a metaphosphate is below 1 σ (Supplementary Fig. 12c), indicating that there is excellent agreement between the experimental data and the model. In contrast, the modeled Fo-Fc for MgF_3_^-^ again showed significant disagreement of electron density features (Supplementary Fig. 12d).

We have considered how a metaphosphate intermediate might be incorporated into a proposed reaction pathway. A metaphosphate is an excellent target for capturing a polarized water molecule, to generate the Pi end-product^45^. We identified a potential candidate, Wat2, in both wild type and Cys150Ser mutant enzymes, and in both cases Wat2 could be activated by the His155 Janus residue (Fig. 6b–d; Fig. 7; Supplementary Fig. 13). The N^δ1^ in His155 is in the N^*ε*2^-protonated *τ* tautomer state, stabilized by a carbonyl oxygen from the backbone of Lys186 (Fig. 6b; Supplementary Fig. 13). In addition, a π-cation interaction can be formed between Arg188 and His155 (Fig. 6b, Supplementary Fig. 13) which is expected to further assist the latter’s deprotonation^46^. Nevertheless, in the wild type enzyme, the 3.7 Å distance and 144° angle from Wat2 to the metaphosphate are not an optimal environment for this reaction, which in addition to the latter’s intensive contacts with P-loop residues (see above), are factors that may assist our crystallographic capture of this proposed intermediate state. In such an event, we propose His155 substitutes for the general base activity of the missing catalytic Asp/Glu), that normally supports this phase of the PTP reaction cycle (Fig. 7; Supplementary Fig. 1). Consistent with this idea, mutation of His155 reduced catalytic activity by 95–98% (Table 1).

In conclusion, our multiple structural snapshots include substrate/enzyme crystal complexes for a Cys-based PTP-phosphatase that lacks a functional canonical catalytic acid. We have linked together these individual structural data-sets (Supplementary Table 1) to construct a reaction cycle that extends the chemical continuum of the PTP family reaction cycle, while not departing from an overall conservation of fundamental structural elements (Fig. 7). Consequently, we can now rationalize how A*t*PFA-DSP1 is optimized for regiospecific and rapid hydrolysis of the β-phosphates of PP-InsP substrates.

In the plant kingdom there is a wide distribution of orthologs of A*t*PFA-DSP1 (Supplementary Fig. 2g); over-expression of PFA-DSPs in transgenic *Arabidopsis* and rice down-regulates immune responses to pathogens^47^. Plant immunity is enhanced by 1,5-InsP_8_^23^, and the latter is a preferred substrate for these PFA-DSPs (Fig. 1). Consequently, we propose a reduction in cellular 1,5-InsP_8_ levels upon phosphatase overexpression can account for the negative impact of these enzymes upon plant immune responses. Similarly, we propose that impaired tolerance to drought upon overexpression of O*s*PFA-DSP in transgenic rice^48^ arises out of an accompanying decrease in PP-InsP levels. Pursuit of A*t*PFA-DSP1 functionality will require further experiments performed in vivo, although we note that the PP-InsP phosphatase activity of the yeast ortholog (S*c*Siw14) has previously been verified in a deletion mutant strain of yeast^20^.

Our structural data also enrich the PTP research field by providing a valuable resource for future practical and theoretical studies to interrogate the reaction cycles of PFA-DSP1s (e.g. Supplementary Fig. 2g), and those of other PTP family members that also operate efficiently without a catalytic acid, such as PTEN^14,15^, human CDC25 phosphatase^49^, MCE1^16^ and PIR1/PIR1^7^ (Supplementary Fig. 2a). Finally, our data represent a significant extension to the known chemical versatility of the WPD loop, thereby greatly extending our understanding of its important contributions to tailoring reactivity profiles for individual PTP family members.

## Methods

### Protein expression and purification

For expression of A*t*PFA-DSP1, we purchased a codon-optimized cDNA (Genscript Inc.) with the following sequence: ATGAAACTGGTTGAAAAAACCACCACCACCGAACAGGATAATGGTGAAGATTTTTGTCGTACCATTATTGAAGTTAGCGAAGTTAATCGTAATGTTTTTCAGGCACCGGGTGGTGAAGCAGATCCGTTTCGTGTTGTTAGCGGTGAAGAACTGCATCTGATTCCGCCGCTGAATTTTTCTATGGTGGATAATGGCATTTTTCGCTCTGGCTTTCCGGATTCTGCTAATTTTTCTTTTCTGCAG ACCCTGGGCCTGCGCTCAATTATTTATCTGTGCCCGGAACCGTATCCGG AATCAAATCTGCAGTTTCTGAAAAGTAATGGTATTCGTCTGTTTCAGTTTGGTATTGAAGGTAATAAAGAACCGTTTGTTAATATTCCGGATCATAAAATTCGTATGGCACTGAAAGTGCTGCTGGATGAAAAAAATCATCCGGTGCTGATTCATTGTAAACGTGGCAAACATCGTACCGGCTGTCTGGTGGGCTGCCTGCGCAAACTGCAGAAATGGTGCCTGACCTCAATTTTTGATGAATATCAGCGCTTTGCGGCTGCGAAAGCCCGCGTGTCAGATCAGCGTTTTATGGAAATTTTTGATGTGAGCAGCTTTAGCCATATTCCGATGAGTTTTAGTTGTTCTATTCGC

The Gateway expression system (Invitrogen) was used to subclone into the pDest-566 vector the cDNAs that encode one of several versions of A*t*PFA-DSP1: either full-length enzyme, or a series of full-length enzymes with single site mutations (using a site-directed mutagenesis kit (Stratagene), or residues 49-215, or residues 49-215 in which Cys150 was mutated (see below). This vector also encodes a 6xHis tag, maltose-binding protein tag, and tobacco etch virus protease cleavage site at the N terminus. All mutants were verified by sequencing. Primers are listed as below (mutations in upper case). All proteins were expressed and purified similarly.

C150S 5′-gaaaaaaatcatccggtgctgattcatTCTaaacgtggcaaacatcgtaccg
C150S 3′-cgatgtttgccacgtttAGAatgaatcagcaccggatgatttttttcatc
H155D 5′-ctgattcattgtaaacgtggcaaaGATcgtaccggctgtctggtggg
H155D 3′-caccagacagccggtacgATCtttgccacgtttacaatgaatcagcac
H155E 5′-ctgattcattgtaaacgtggcaaaGAAcgtaccggctgtctggtggg
H155E 3′-caccagacagccggtacgTTCtttgccacgtttacaatgaatcagcac
H155L 5′-ctgattcattgtaaacgtggcaaaCTGcgtaccggctgtctggtggg
H155L 3′-caccagacagccggtacgCAGtttgccacgtttacaatgaatcagcac
T157A5′-cattgtaaacgtggcaaacatcgtGCCggctgtctggtgggctg
T157A 3′-ccaccagacagccGGCacgatgtttgccacgtttacaatgaatc
D191A 5′-gcccgcgtgtcaGCtcagcgttttatggaaatttttgatgtgagc
D191A 3′-caaaaatttccataaaacgctgaGCtgacacgcgggctttcg

The recombinant plasmids were transformed into DE3 competent *E. coli* cells (Stratagene) that were pretransformed with chaperone plasmid pGro7 (Takara Clontech). An overnight culture of the transformed *E. coli* cells was inoculated into nutrient-rich 2 × YT medium (16 g/liter Tryptone, 10 g/liter yeast extract, 5.0 g/liter NaCl) supplemented with 0.07% (w/v) L-arabinose at pH 7.5 and grown at 37 °C to an *A*_595_ of 0.7. Isopropyl β-d-thiogalactopyranoside (0.1 mM) was then added, and cultures were continued at 15 °C for 20 h.

The cells were harvested by centrifugation at 5000 x g for 10 min and disrupted using a constant cell disruption system (Constant Systems Ltd.) under 20 k.p.s.i. Recombinant wild type (WT) and mutant proteins were purified by several chromatographic procedures performed at 4 °C. First, the protein was applied to a nickel-nitrilotriacetic acid-agarose column (Qiagen), which was then washed with buffer containing 300 mM NaCl, 20 mM Tris-HCl (pH 7.2), 20 mM imidazole. Protein was eluted by increasing the imidazole concentration to 400 mM. Next, the eluate was applied to a HiTrap™ Heparin HP column (Cytiva) and eluted with 10 column volumes of a 50–2000 mM NaCl gradient in 20 mM Tris-HCl (pH 7.2). After cleavage using the tobacco etch virus protease, the protein was further purified using another HiTrap Heparin HP column followed by a Superdex™ 200 gel filtration column (Cytiva) that was eluted with 150 mM NaCl, 20 mM Tris-HCl (pH 7.2). Purified proteins were concentrated to 0.4–10 mg ml^−1^ and stored in aliquot at −80 °C. It is likely Pi in the protein’s active site (see Figures) is captured during the bacterial cultures, since Pi was not added to either the purification buffers or the crystallization buffers (see below).

### Crystallization

The crystallization of core catalytic domain of A*t*PFA-DSP1 (residues 49–215) was optimized by hanging drop vapor diffusion against a well buffer of 0.4 M NaCl, 50 mM β-mercaptoethanol at 25 °C (3 μl of 5.5 mg/ml protein plus 1 μl of well buffer in the crystallization drop). The formed crystals were soaked in 30% PEG400, 13 mM MgCl_2_, 33 mM NaF, 50 mM β-mercaptoethanol, 66 mM HEPES, pH 7.2, and 0.05–10 mM ligands. For some experiments, the soaking buffer was changed to either 66 mM Tris-HCl (pH 8.0) or 50 mM N-cyclohexyl-2-aminoethanesulfonic acid. Soaking was performed at 25 °C for up to six days. More details for each of the resulting crystal complexes are listed in Supplementary Table 1.

### Data collection, structure determination, and refinement

Diffraction data were collected using Advanced Photon Source beam line 22-ID and 22-BM. All data were processed with the program HKL2000^50^. The complex structures of AtPFA-DSP1 were determined by molecular replacement from the previously reported A*t*PFA-DSP1 structure that has a significantly lower atomic resolution and did not contain any potential substrates (PDB: 1XRI).The initial structure was further rebuilt with Coot^51^ and refined with REFMAC^52^ from the CCP4 package. The molecular graphics representations were prepared with the program PyMOL (Schrödinger, LLC). Atomic coordinates and structure factors have been deposited in the Protein Data Bank under accession codes 7MOD, 7MOE, 7MOF, 7MOG, 7MOH, 7MOI, 7MOJ, 7MOK, 7MOL and 7MOM.

### Enzyme assays

The catalytic activity of full-length A*t*PFA-DSP1 was originally screened using 10 μM concentrations of each of the PP-InsPs and the other indicated organic phosphates. All of the InsP_7_ isomers, and the 1,5-InsP_8_, were chemically synthesized and characterized as described previously^53,54^. New syntheses of 5-InsP_7_ and 1,5-InsP_8_ were performed as described previously^53,55^, and purity was verified as >95% by capillary electrophoresis mass spectrometry^56^ (Supplementary Fig. 14a,b). Previous work^25,54,57^ has described the synthesis and characterization of the other InsP_7_ isomers that have been used in this study, as well as the 5-PP-InsP_4_, and the 5-PCF_2_Am-InsP_5_. All other organic phosphates were purchased from Sigma–Aldrich. Technical replicates of the same protein sample were each performed in independent assays. Reactions (100 μl; 30 °C; 30 min) contained 20 mM HEPES (pH 7.2), 100 mM KCl, 0.8 mM MgCl_2_, and 20 μM EDTA. Reactions were quenched with 100 μl of phosphate detection reagent (36:1 v/v of 2.6% sodium molybdate in 2.5 M HCl: 0.126% malachite green chloride). Pi release was quantified from the absorbance at 620 nm^58^. In some assays, 10 mM β-mercaptoethanol was added to the reaction buffer; there was less than a 5% change in enzyme activity. Subsequently, reaction kinetics were determined for the most efficiently hydrolyzed substrates (1,5-InsP_8_, 4-InsP_7_, 5-InsP_7_, 6-InsP_7_ and 5-PP-InsP_4_), at various concentrations as described in the figures. Data were analyzed using GraphPad Prism.

Mutant versions of A*t*PFA-DSP1 were assayed as described above except for the C150S version; its low activity could not be accurately determined with the colorimetric method. Instead, 10 μM 5-InsP_7_ substrate was spiked with [^3^H]-radiolabeled 5-InsP_7_ (prepared as previously described^59^), reactions were quenched with perchloric acid^59^, neutralized^59^, and analyzed using a 250 × 4.6 mm Synchropak Q100 HPLC column. The elution gradient was generated by mixing Buffer A (1 mm Na_2_EDTA) with Buffer B (Buffer A plus 2.5 M NH_4_H_2_PO_4_, pH 4.0) as follows: 0–1 min, 30% B; 1–21 min, 30–60% B, 21–24 min, 60–70% B. The eluate was mixed in-line with 2.5 volumes of MonoFlow 4 scintillation fluid (National Diagnostics), and radioactivity was assayed with a BetaRam 6 C detector (LabLogic) using Laura6 data collection software.

### Molecular dynamics simulations

The starting configurations for the molecular dynamics trajectories were based on PDB ID 7MOK; missing atoms and protons were introduced by using the leap module of Amber.18^60^, 29 Na^+^ and 29 Cl^−^ ions were added to provide the 100 mM effective ionic concentration, plus an additional three Cl^−^ ions for charge neutralization. The system was solvated in a box of TIP3P water with the box boundary extending to 20 Å from the nearest peptide atom (resulting in 50,481 atoms in the simulation box). All Lys, Arg, Glu and Asp residues are considered to be in their charged states. His149 and His155 were considered δ-protonated due to their proposed hydrogen-bonding with ligand; remaining His residues were deemed ε-protonated. Prior to equilibration, the solvated system was sequentially subjected to (1) 500 ps belly dynamics with fixed peptide, (2) minimization (5,000 steps), (3) constant temperature (200 K) and constant pressure (1 atm) dynamics (~1 ns) at fixed protein to assure a reasonable starting density around 1 g/cc, (4) minimization (5,000 steps), (5) step-wise heating MD at constant volume (to bring the temperature up to 300 K in 3 ns), and (6) constant volume simulation for 10 ns with a constraint force constant of 10 kcal/mol applied only on backbone heavy atoms. After releasing all constraining forces within the next 20 ns of the equilibration period, sampling was increased by performing four independent, constant temperature (Langevin thermostat) constant volume molecular dynamics simulations for 2 μs each. All trajectories were calculated using the PMEMD module of Amber.18 with 1 fs time step. Long range coulombic interactions were handled using the PME method with a 10 Å cut-off for the direct interactions. The amino-acid parameters were selected from the FF14SB forcefield of Amber.18, the phosphate forcefield was selected from the gaff2 parameters in Amber.18, and the charges (Supplementary Table 2) were generated from single point B3LYP/6-31 G* calculations of an optimized geometry using Gaussian-09^61^. The partial atomic charges and the gaff2 atom types used for Pi in the study are provided in Supplementary Table 3. At the salt concentration of 100 mM, the MMGBSA module with the standard parameters was used to estimate binding energies from 100 samples selected from molecular dynamics simulations for each pose of Pi.

### Reporting summary

Further information on research design is available in the Nature Research Reporting Summary linked to this article.

## Supplementary Material

The online version contains supplementary material available at https://doi.org/10.1038/s41467-022-29673-y.

Supplementary Information

Supplementary Movie 1

Supplementary Movie 2

Supplementary Movie 3

Supplementary Movie 4

## Figures and Tables

**Table 1:** Mutagenic analysis of dephosphorylation by core catalytic domain *AtPFA-DSP1*^49–215^.

	WT	C150S	H155D	H155E	H155L	T157A	D191A
Speciﬁc activity nmol min^−1^ mg^−1^	674 ± 60 (7)	0.15 ± 0.03 (6)	35.1 ± 2.7 (7)	15.8 ± 1.4 (9)	12.6 ± 0.1 (6)	15.1 ± 0.8 (9)	46.2 ± 1.5 (7)
Relative activity %	100	0.02	5.2	2.3	1.9	2.2	6.9

Data are means and standard errors; the numbers of independent experiments (see Methods) are provided in parentheses. Experiments were performed with 10 _μ_M 5-InsP_7_; a colorimetric assay was used to determine Pi release, except for the C150S mutant, for which 5-[^3^H]InsP_7_ hydrolysis was directly monitored by HPLC (see Methods and Supplementary Fig. 3). Source data are provided as a Source Data file.

## Data Availability

The data that support this study are available from the corresponding authors upon reasonable request. Structural data generated during the course of this study are available at the PDB under accession codes 7MOD, 7MOE, 7MOF, 7MOG, 7MOH, 7MOI, 7MOJ, 7MOK, 7MOL, 7MOM. Previously reported structural data used in the course of this study are available at the PDB under the following accession codes: 1XR1, 2YF0, 3RGO, 3I7Z, 6E3B, 6BYF, 7DHF. Source data are provided with this paper.
