# Supplementary material for: A structural exposé of noncanonical molecular reactivity within the protein tyrosine phosphatase WPD loop
Source: Nat Commun. Author manuscript; Available in PMC 2022 May 2. (PMC9038691; doi:10.1038/s41467-022-29673-y)
Supplement: Supplementary Information [file EMS144585-supplement-Supplementary_Information.pdf]

Supplementary Information

A structural *exposé of* noncanonical molecular reactivity within the protein tyrosine phosphatase WPD loop

Huanchen Wang[1]*, Lalith Perera[2], Nikolaus Jork[3], Guangning Zong[1], Andrew M. Riley[4], Barry V.L. Potter[4], Henning J. Jessen[3] and Stephen B. Shears[1]*

*Corresponding authors. Emails shears@niehs.nih.gov; huanchen.wang@nih.gov

This PDF file includes:

Figs. 1 to 12
Tables 1-3

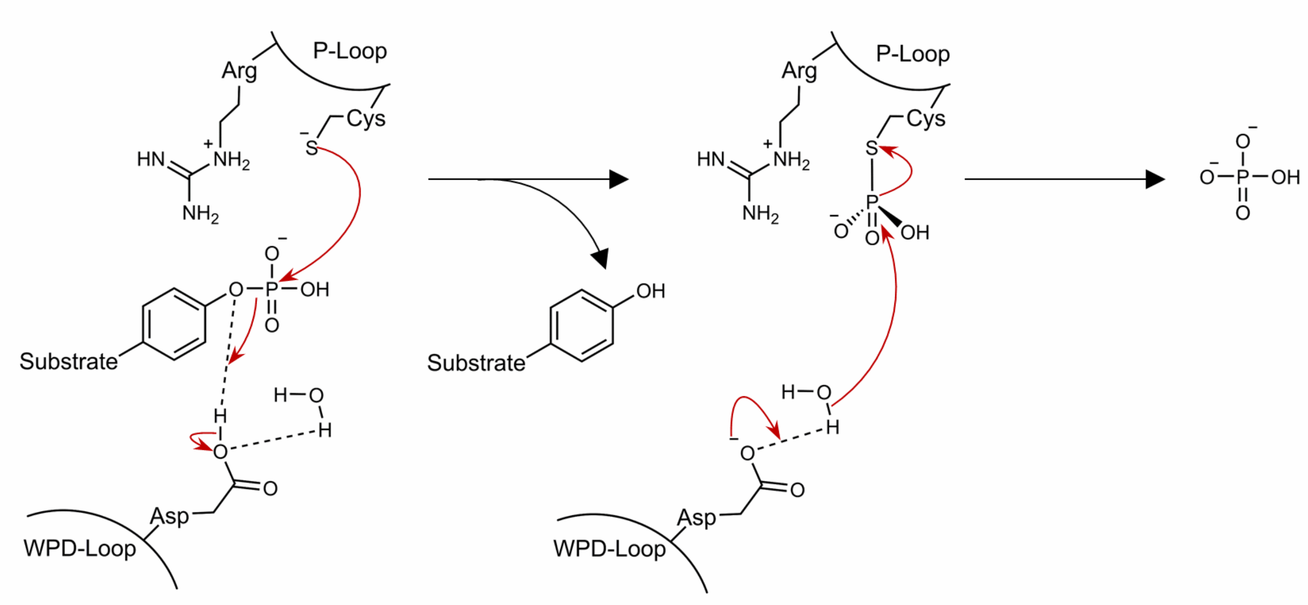

Supplementary Figure 1. The canonical catalytic reaction mechanism of cysteine-based protein tyrosine phosphatase (PTP). Substrate hydrolysis is assisted by a catalytic general acid/base, typically an aspartate from the WPD loop. A thiophosphate intermediate is formed during catalysis. In the next step, an aspartate residue (or glutamate in some cases) activates a water molecule for nucleophilic attack upon a thiophosphate intermediate. A free phosphate ion is formed.

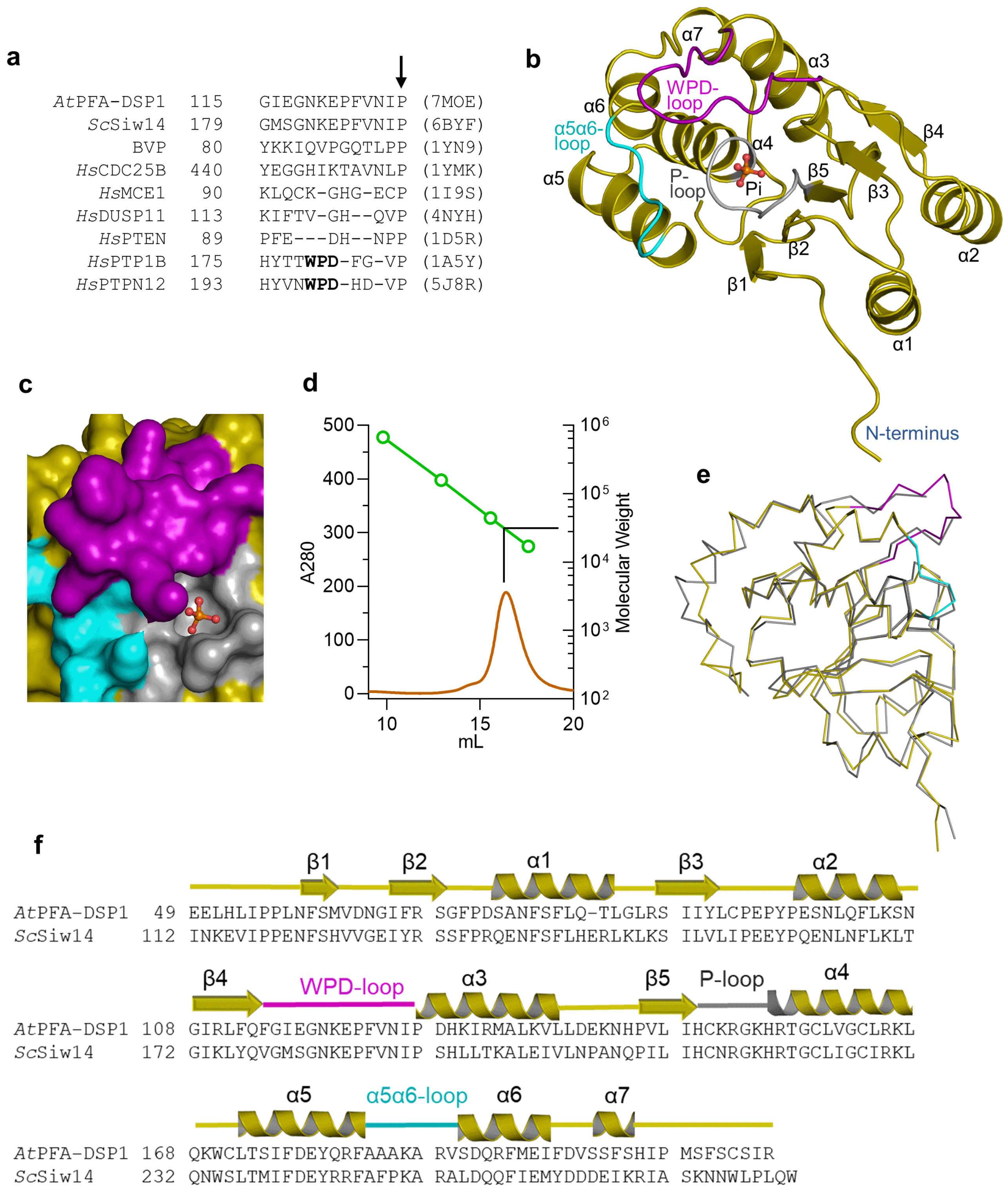
a
AtPFA-DSP1 115 GIEGNKEPFVNIP (7MOE)
ScSiw14 179 GMSGNKEPFVNIP (6BYF)
BVP 80 YKKIQVPGQTLPP (1YN9)
HsCDC25B 440 YEGGHIKTAVNLP (1YMK)
HsMCE1 90 KLQCK-GHG-ECP (1I9S)
HsDUSP11 113 KIFTV-GH--QVP (4NYH)
HsPTEN 89 PFE---DH--NPP (1D5R)
HsPTP1B 175 HYTTWPD-FG-VP (1A5Y)
HsPTPN12 193 HYVNWPD-HD-VP (5J8R)
b
α7
α3
WPD-loop
α6
α5α6-loop
α4
β4
α5
P-loop
Pi
β5
β3
β2
α2
β1
α1
N-terminus
c
d
500
400
300
200
100
0
A280
10
15
20
mL
10^6
10^5
10^4
10^3
10^2
Molecular Weight
e
f
β1 β2 α1 β3 α2
AtPFA-DSP1 49 EELHLIPPLNFSMVDNGIFR SGFPDSANFSFLQ-TLGLRS IIYLCPEPYPESNLQFLKSN
ScSiw14 112 INKEVIPPENFSHVVGEIYR SSFPRQENFSFLHERLKLKS ILVLIPEEYPQENLNFLKLT
β4 WPD-loop α3 β5 P-loop α4
AtPFA-DSP1 108 GIRLFQFGIEGNKEPFVNIP DHKIRMALKVLLDEKNHPVL IHCKRGKHRTGCLVGCLRKL
ScSiw14 172 GIKLYQVGMSGNKEPFVNIP SHLLTKALEIVLNPANQPIL IHCNRGKHRTGCLIGCIRKL
α5 α5α6-loop α6 α7
AtPFA-DSP1 168 QKWCLTSIFDEYQRFAAAKA RVSDQRFMEIFDVSSFSHIP MSFSCSIR
ScSiw14 232 QNWSLTMIFDEYRRFAFPKA RALDQQFIEMYDDDEIKRIA SKNNWLPLQW

g

```
Zm      -----------------------------------------LVSAEELLLVPPLNFA 16
Dc      --------------SDTCRTIGV----------AVVSKSPPATGDYDDDLQFTPPFNFA 35
Os      --------------------------------------------GEEATLVPPLNFA 13
At      MKLVEKTTTTEQDNGEDFCRTIIEVSEVNRNVFQAPGGEADPFRVVSGEELHLIPPLNFS 60
Pp      -------------------------------------------VVGDELNLIPPLNFA 15
Rc      --------------------------------------------------IPPLNFA 7
Cs      --------------------------------------------------IPPLNFS 7

                                                          WPD-loop
Zm      MVDHGVYRSGFPDASNLPFLETLRLRSVLCLCPEPYPEANLEFLRAHGIKLFQFGIDGSK 76
Dc      MVDHGIYRSGFPDPTNFSFIQTLGLRSIVYLCPEPYPEANVEFLRSNGIRLFQFGVDGSK 95
Os      MVDDGIFRSGFPAAANFRFLKSLNLRSIVYLCPEPYPETNAEFLAKNGIKLHQFGIEGRK 73
At      MVDNGIFRSGFPDSANFSFLQTLGLRSIIYLCPEPYPESNLQFLKSNGIRLFQFGIEGNK 120
Pp      MVDNGIFRSGFPDSANFAFLQTLGLRSIICLCPEPYPEATTEFLKDGGIRLYQFGIEGYK 75
Rc      MVDNGIFRSGFPDSNNLSFLQTLGLRSIICLCPEPYPETNVEFLKSNGIKLFQFGIEGYK 67
Cs      MVDNGIFRSGFPDSANFSFLQTLGLRSIICLCPEPYPDINMEFLKSNGIRLFQFGIESNK 67

      WPD-loop                 P-loop
Zm      EPFVNIPEDRIREALEVILDASNHPVLIHCKRGKHRTGCVVGCFRKLQRWCLTSIFDEYQ 136
Dc      EPFVNIPEETIREALKTVIDIRNHPL-IHCKRGKHRTGCVVGCLRKLQRWCLTSIFDEYQ 154
Os      EPFVNIPDDKIREALKVVLDVKNQPLLIHCKRGKHRTGCVVGCLRKLQKWCLSSVFDEYQ 133
At      EPFVNIPDHKIRMALKVLLDEKNHPVLIHCKRGKHRTGCLVGCLRKLQKWCLTSIFDEYQ 180
Pp      EPFVNIPEDTIREALKVVLDAKNHPVLIHCKRGKHRTGCLVGCLRKLQKWCLSSIFDEYQ 135
Rc      EPFINIPEDTIREALKVVLDVRNHPILIHCKRGKHRTGCLVGCLRKLQRWCLSSVFDEYQ 127
Cs      EPFVNIPEDTIREALQVVLDVKNHPVLIHCKRGKHRTGCLVGCIRKLQRWCLSSVFDEYQ 127

         α5α6-loop
Zm      RFAAAKTRVSDLRFMELFDVSSIKHLAFESS----   167
Dc      RFAAVKARVSDQRFMELFDVSTFKHIPKEFSC---   186
Os      RFAAAKARSTDQRFMELFDISSLKHLTAS------   162
At      RFAAAKARVSDQRFMEIFDVSSFSHIPMSFSCSIR   215
Pp      RFAAAKARVSDQRFMELFDVSTLKHMPMSFSCSKR   170
Rc      RFAAAKARVADQRFMEMFDVSSLKHLPMPFSCSKR   162
Cs      RFAAAKARVSDQRFMELFDVSSLKHQPMSFSCS--   160
```

Supplementary Figure 2. Overall structure of *At*PFA-DSP1$^{49\text{-}215}$. **a**, A structure-based, manual alignment of the amino-acid sequences of the WPD loops of PTP1B and PTPN12 (WPD motif highlighted in bold font), plus the sequences of corresponding loops in proteins that each lack the WPD motif. PDB numbers given in parentheses. The arrow highlights a conserved Pro residue that may act as a hinge to help direct the range of motion of the loop. **b**, Ribbon diagram of *AtPFA-DSP1*$^{49\text{-}215}$ in complex with trapped Pi. Coloring and naming of structural elements correspond to those previously used for *Sc*Siw14 [1]: grey for P-loop, cyan for α5-α6 loop, purple for WPD-loop, and yellow for the remainder. The Pi is depicted in ball and stick format (phosphorus is colored orange, and oxygen is red) **c**, Surface representation colored to match the structural elements shown in panel a. **d,** Gel filtration analysis. The apparent molecular weight is 22.0 kDa which is consistent with the theoretical monomeric molecule weight 19.6 kDa. **e,** Superimposition of Cα traces of *Sc*Siw14$^{116\text{-}281}$ (PDB accession code 6BYF; transparent white) and *At*PFA-DSP1$^{49\text{-}215}$ (PDB accession code 7MOK; yellow). **f,** Sequence alignment of *At*PFA-DSP1$^{49\text{-}215}$ and

*Sc*Siw14$^{112\text{-}281}$. The secondary structural elements are also given above the alignment. **g**, Candidate orthologs of *At*PFA-DSP1 identified through a BLAST search of several plant genomes assembled into the EnsemblPlants database (https://plants.ensembl.org). These sequences were aligned with Clustal Omega. The indicated loop sequences are those identified for AtPFA-DSP1. Accession numbers are as follows: *Zm*, *Zea mays*, Zm00001eb095500_P001; *Os*, *Oryza sativa*, ASM465v1; *Dc*, *Daucus carota* ASM162521v1; *Pp*, *Populus trichocarpa* POPTR_014G159100v3; *Rc, Rosa chinensis,* RchiOBHm-V2; *Cs*, *Cannabis sativa* evm.TU.10.1109.

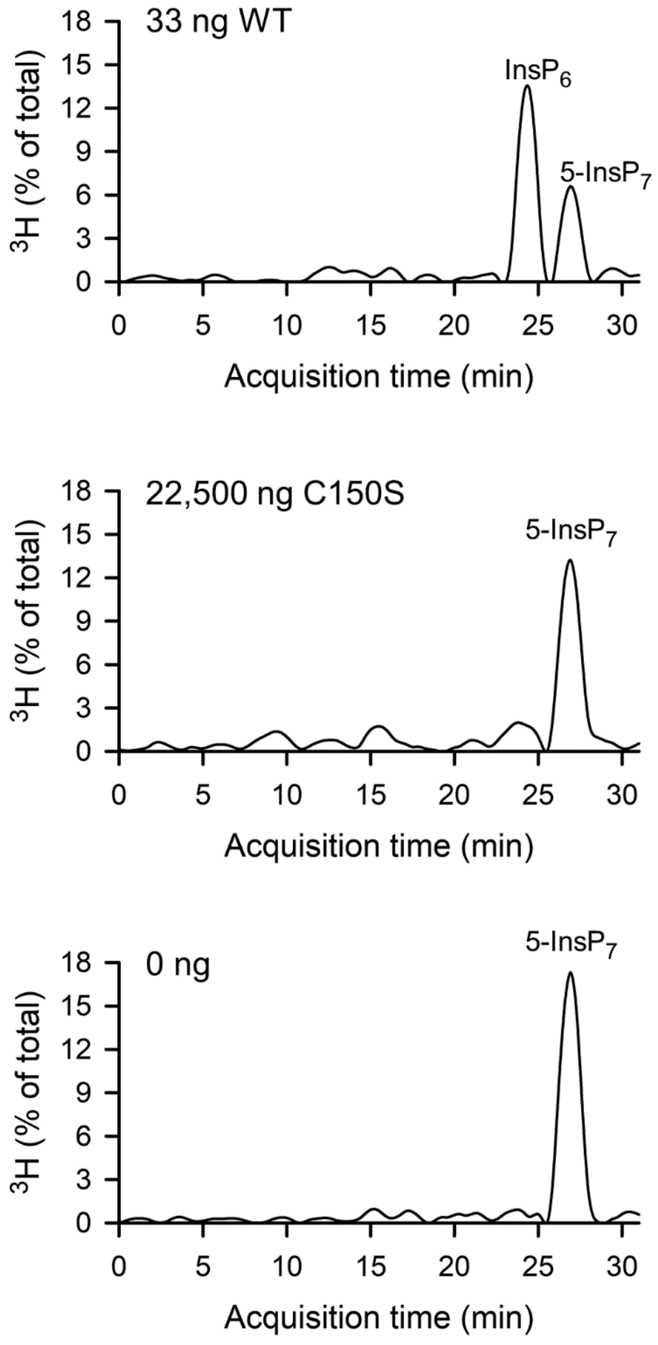

Supplementary Figure 3. HPLC analysis of the activity of WT and C150S *At*PFA-DSP1$^{49\text{-}215}$ towards 5-[^{3}H]InsP$_7$. HPLC analysis of enzyme assays was performed as described in the Methods; ^{3}H-elution profiles were exported from the in-line BetaRam radioisotope detector into SigmaPlot, which performed Loess smoothing and peak integrations. A representative dataset is shown. Mean values of reaction rates for the WT and C150S mutant are (means ± standard errors from 6 independent experiments): 593 ± 54 and 0.15 ± 0.03 nmol min^{-1} mg^{-1} respectively. Source data are provided as a Source Data file.

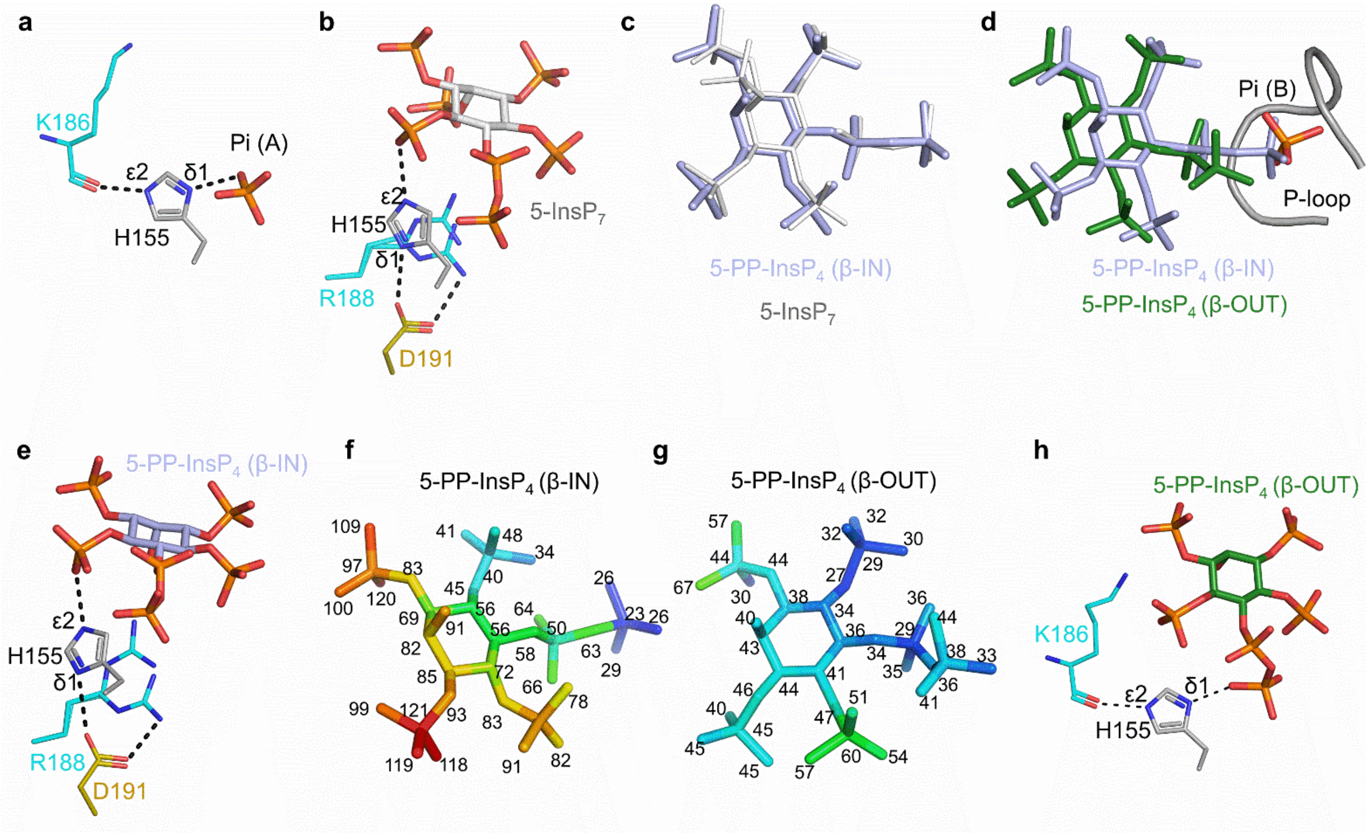

Supplementary Figure 4. Orientations of various *At*PFA-DSP1 ligands and their interactions with His155. In the stick format graphics, phosphorus is orange, oxygen is red, and nitrogen is dark blue (unless otherwise indicated), and the coloration of the protein reflects various structural elements, i.e., P-loop: carbon is gray, σ5/σ6 loop, carbon is cyan; σ6 helix, carbon is gold. Polar contacts are depicted with broken black lines. Panel **a** shows polar contacts that involve residue His155 when the catalytic pocket contains Pi(A). Panel **b** shows polar contacts that involve residue His155 and D191 when 5-$InsP_7$ is bound. Panel **c** is a superimposition of bound 5-$InsP_7$ and the β-IN pose of 5-PP-$InsP_4$. Panel **d** is a superimposition of 5-PP-$InsP_4$ in the β-IN (lavender) and β-OUT (green) orientations (the centers of each carbon ring are 1.4 Å apart). Panels **e** show polar contacts that involve His155 and D191 when the catalytic pocket contains 5-PP-$InsP_4$ β-IN. Panels **f** and **g** show β-factor values (rainbow format: dark blue = 20, red = 120) for 5-PP-$InsP_4$ in the β-OUT and β-IN orientations, respectively. Panels **h** show polar contacts that involve His155 and D191 when the catalytic pocket contains 5-PP-$InsP_4$ β-OUT. Source data are provided as PDB accession code 7MOH.

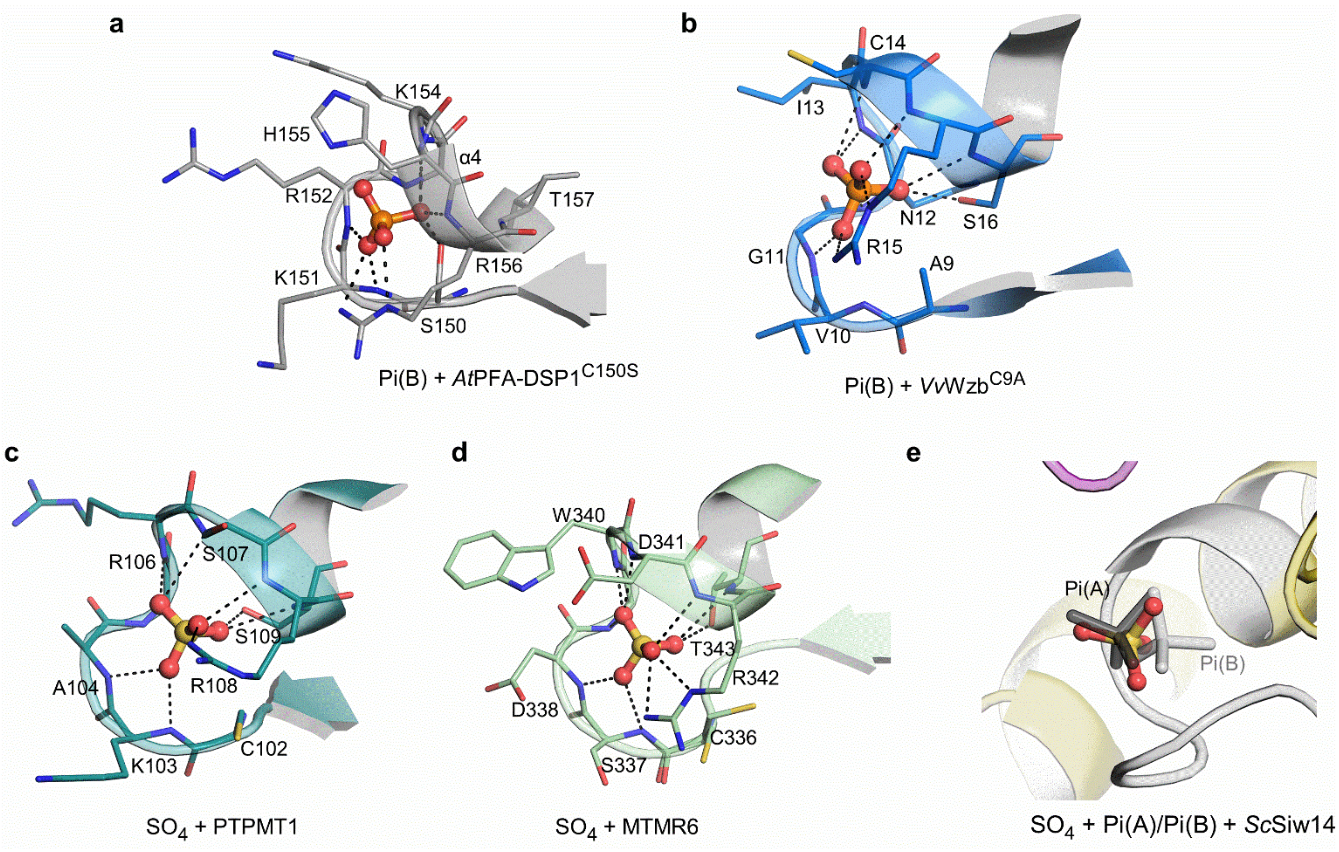

Supplementary Figure 5. Structures of the catalytic pockets of selected PTPs with either Pi or a sulfate ion. a, *At*PFA-DSP$^{149\text{-}215,C150S}$ in complex with PP-InsP4 OUT (not shown) and Pi in pose B. **b**, *Vv*Wzb^{Cys9Ala} in complex Pi in pose B (PDB: 7DHF). **c**, **d**, PTPMT1 (PDB: 3RGO) and MTMR6 (PDB: 2YFO) respectively, each of which contains a sulfate ion in a configuration that closely mimics the Pi(B) conformation. **e,** superimposition of sulfate, in crystal complex with *Sc*Siw14 (PDB: 6E3B), upon Pi(A) (see Fig 2a in the main text; dark gray) and Pi(B) (see Fig. 5a in the main text; light gray) in complex with *At*PFA-DSP1. In each panel, relevant aspects of each protein are shown in a mixture of ribbon and stick formats. Nitrogens are blue, oxygens are red, and sulfur is orange. Broken black lines depict polar interactions with the bound ion, depicted in stick and ball format.

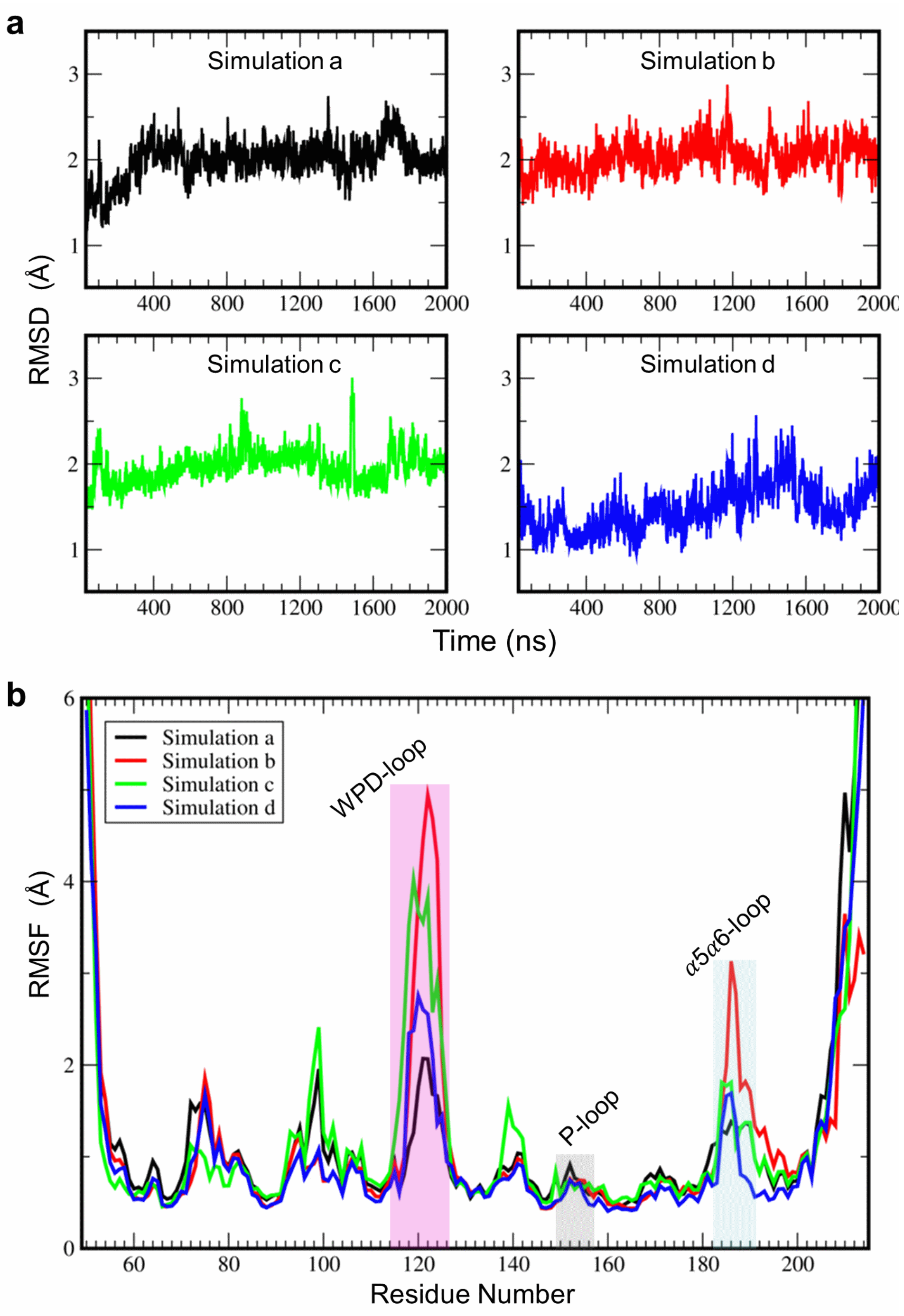

Supplementary Figure 6. Molecular dynamics simulations of global and local fluctuations of *At*PFA-DSP1$^{49-215}$. Four separate 2μs molecular dynamics simulations were initiated with a crystal complex of *At*PFA-DSP1$^{49-215}$ with Pi(A) as the ligand (PDB accession code 7MOK; Supplementary Table 1). **a**, the root mean square deviations of the heavy backbone atoms of *At*PFA-DSP1$^{49-215}$; simulation a is the source of the data given in Fig. 5d,e in the main text. The colors of each trace are retained in Supplementary Figures 9 and 10 to facilitate comparisons. **b**, root mean square fluctuations for backbone Cα atoms; line coloring corresponds to each of the simulations shown in

panel **a**. The WPD-loop, P-loop and α5α6-loops are highlighted with purple, gray and blude shading, respectively. Source data are provided as a Source Data file.

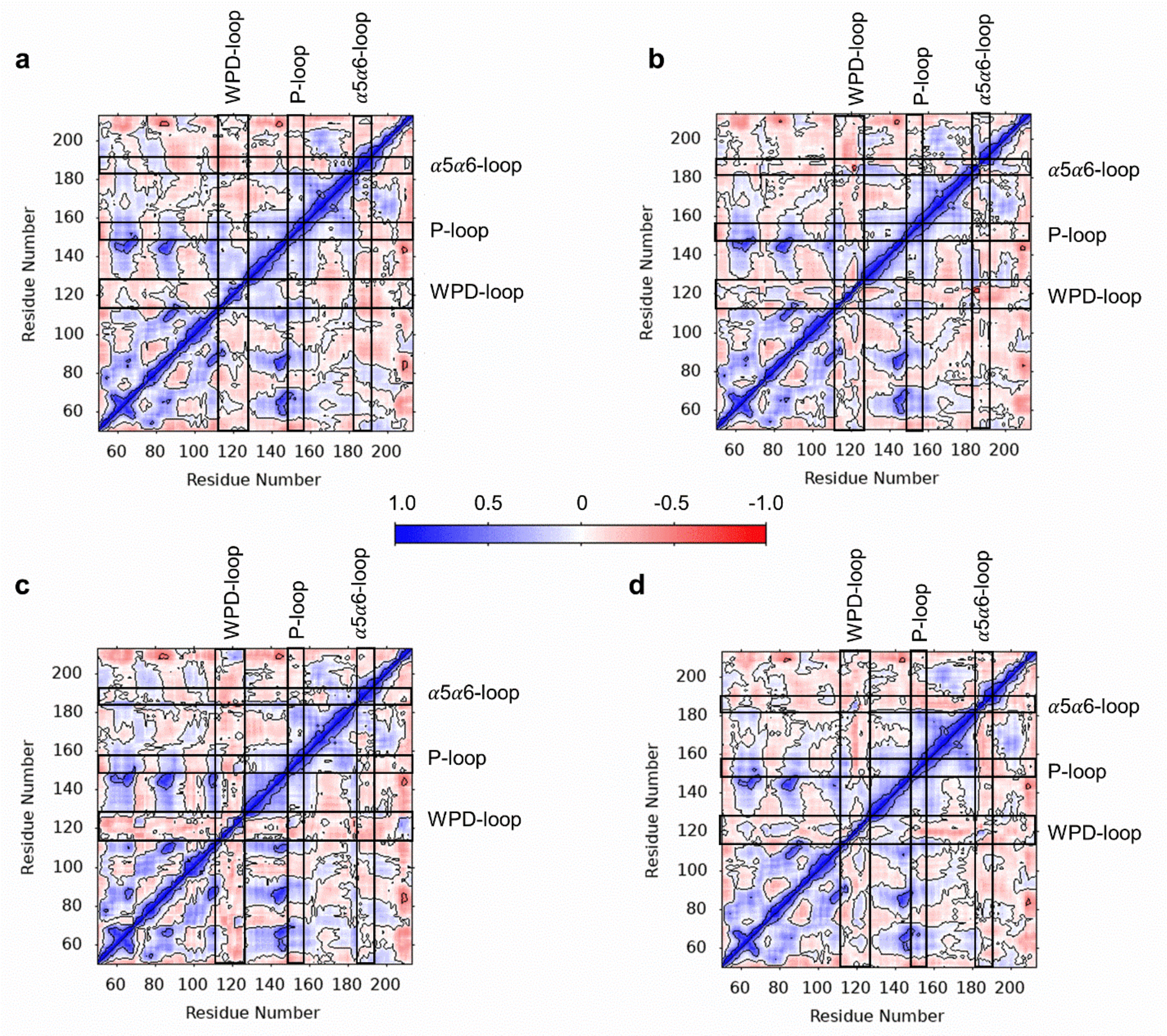

Supplementary Figure 7. Dynamic cross correlation matrix obtained from molecular dynamics simulations of *At*PFA-DSP1$^{49\text{-}215}$. The data in panel a are derived from the simulation provided in Fig. 5 d,e in the main text. Data in panels b, c and d were derived from the simulations b,c and d described in Supplementary Fig. 6. Dynamic cross correlations between residue pairs (presented as a heat map) were calculated from the distances between their Cα atoms. The central horizontal bar is the key to the heat map that represents the degree of correlation (blue) or anti-correlation (red). Source data are provided as a Source Data file.

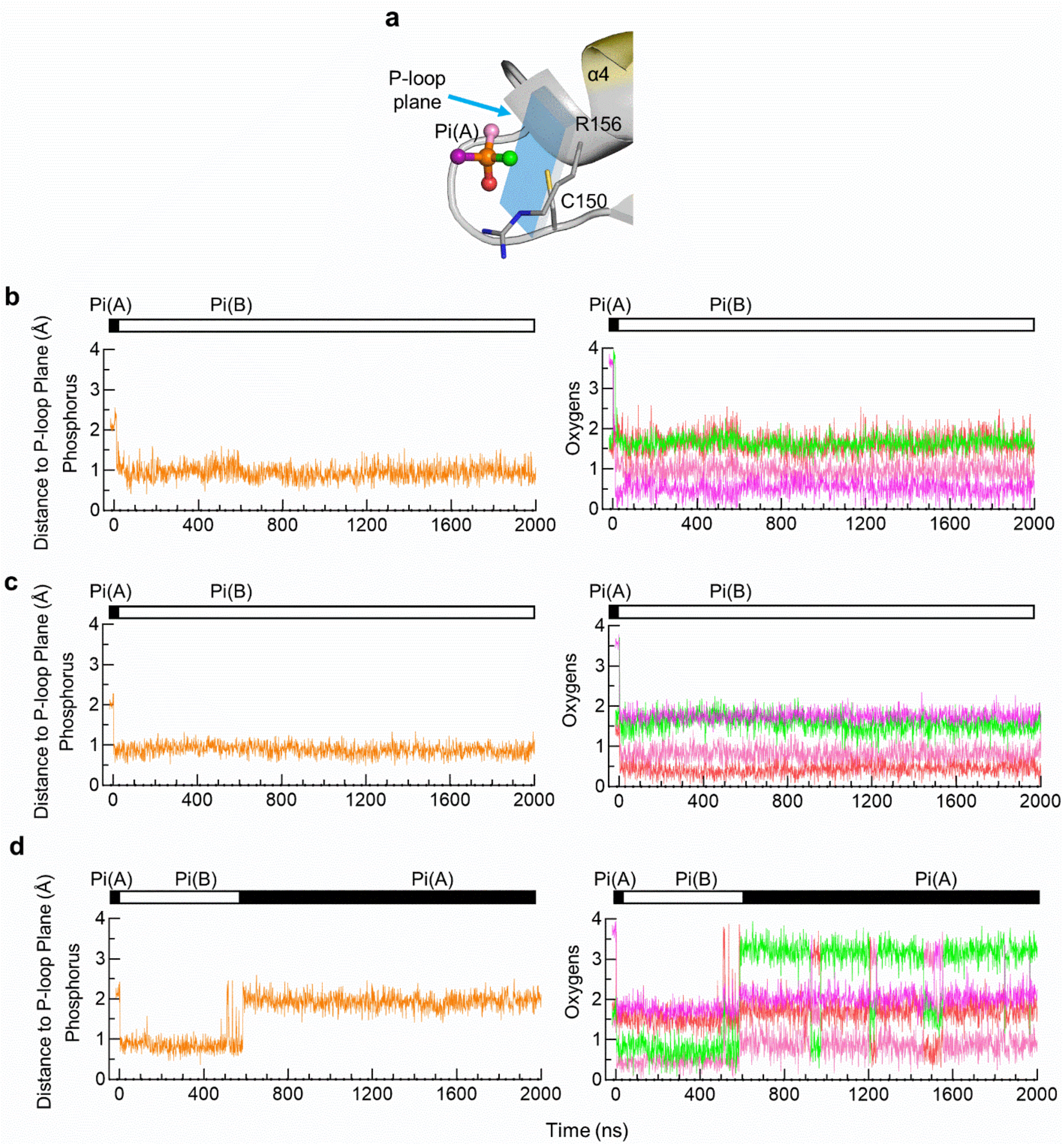

Supplementary Figure 8. Molecular dynamics simulations of phosphate mobility inside the catalytic center of *At*PFA-DSP1[49-215]. Panel **a**, initial configuration of Pi(A) for the simulations, in relation to the P-loop plane (see also Fig. 5e in the main text). Panels **b**, **c** and **d** depict the simulated movements of individual atoms of di-anionic Pi, relative to the fixed P-loop plane; data were sourced from simulations b,c and d described in Supplementary Fig. 6a. Horizontal bars illustrate the switching between Pi poses A (white) and B (black). Source data are provided as a Source Data file.

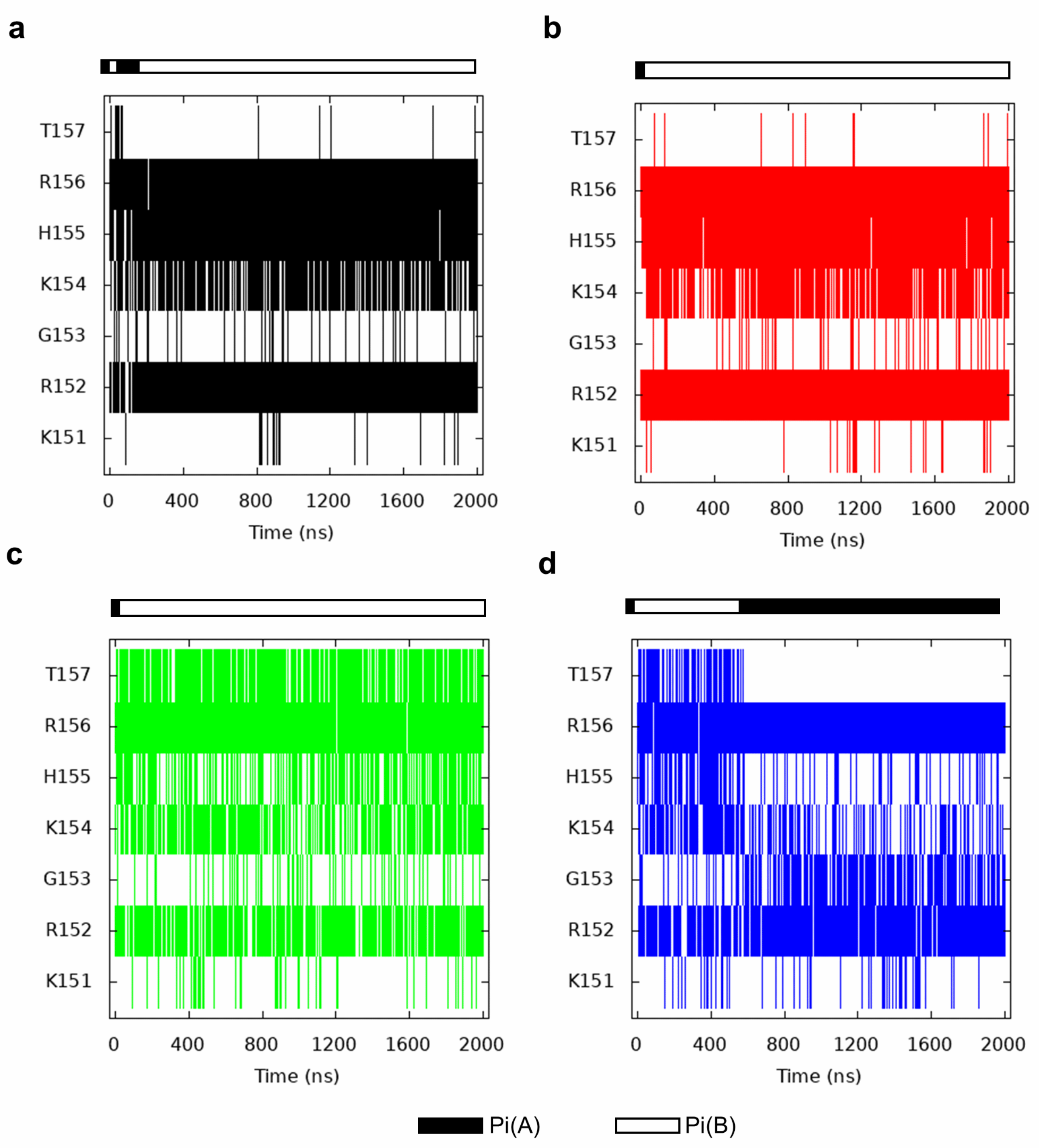

Supplementary Figure 9. Simulated ionic interactions between bound Pi and P-loop residues in *At*PFA-DSP1$^{49\text{-}215}$. The data in panel a are sourced from the simulation shown in Fig. 5 d,e in the main text.. Data in panels b, c and d were derived from Supplementary Fig. 6a,b (simulations b, c and d respectively; the same color coding is retained). Horizontal bars illustrate the switching between Pi poses A (black) and B (white). Source data are provided as a Source Data file.

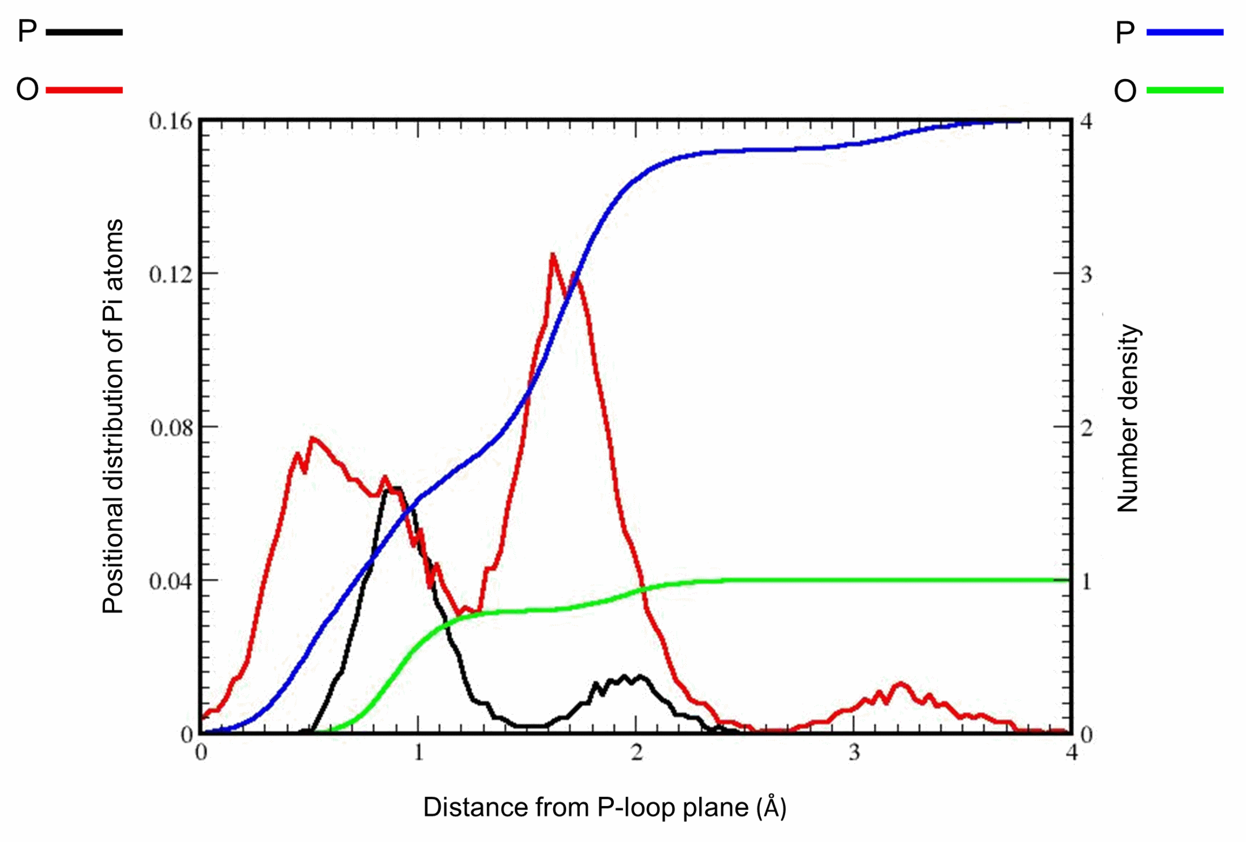

Supplementary Figure 10. Distributions of atomic distances from Pi atoms to the P-loop plane of *At*PFA-DSP1[49-215]. These data are collected from all four simulations (Supplementary Fig. 5a). The left-hand y-axis shows the positional distribution of phosphorous (P; black line) and all four oxygens (O; red line). The right-hand y-axis shows the number density of phosphorous (P; blue line) and all oxygen atoms (green line). The source data are provided as a Source Data file.

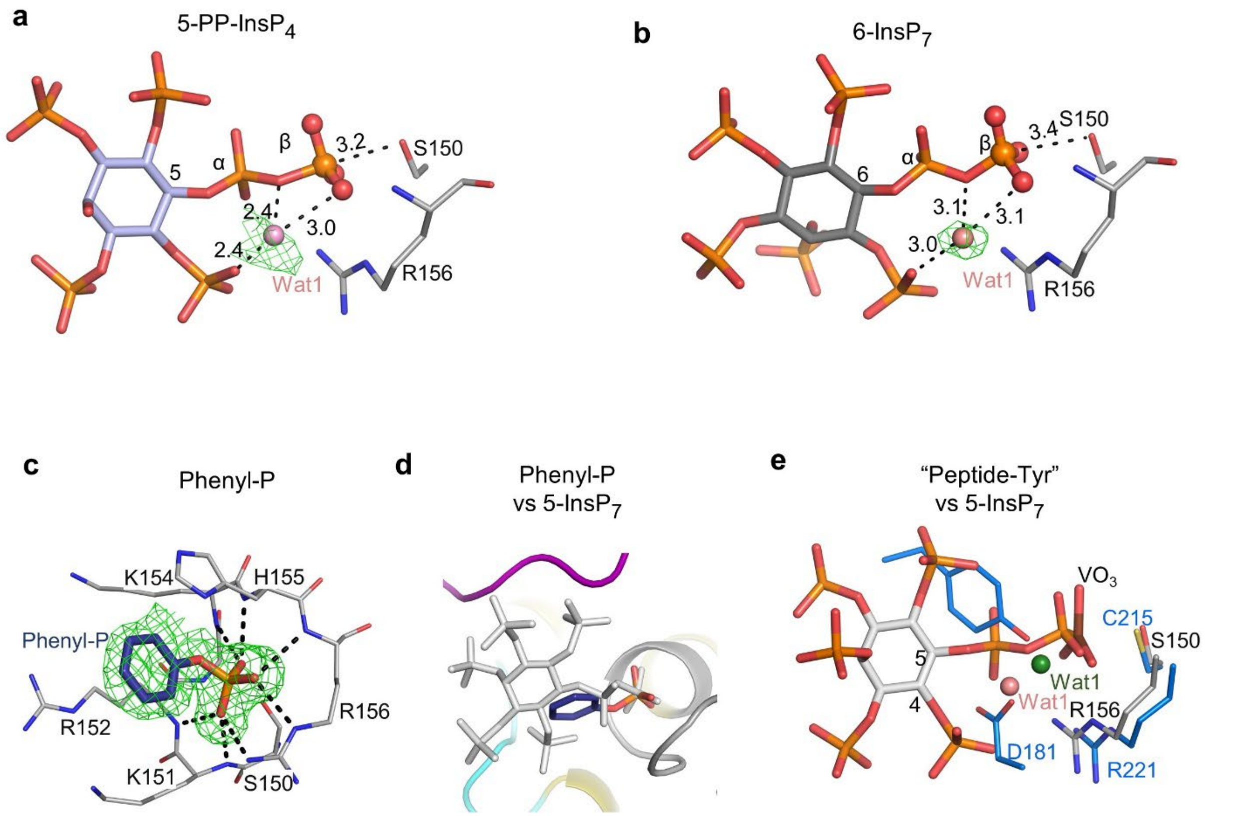

Supplementary Figure 11. Analysis of the catalytic environment for *At*PFA-DSP1 in the absence of an Asp/Glu catalytic acid. Data are shown from ligand/enzyme crystal complexes of *At*PFA-DSP1$^{49\text{-}215;\ C150S}$. Panel **a-c**, describe the positions of 5-PP-InsP$_4$, 6-InsP$_7$ and phenyl phosphate ('Phenyl-P') respectively. Pink spheres depict Wat1 (which is not found in the complex with Phenyl-P). The omit Fo-Fc electron density maps are contoured at 3σ and shown in green mesh. Contacts are highlighted with black dashed line and distances are in Å. Panel **d** is a superimposition of bound 5-InsP$_7$ (white stick) and Phenyl-P (blue stick). Panel **e** is a superimposition of enzyme-bound 5-InsP$_7$ and PTP1B (marine blue) with bound peptide-tyrosine (colored blue and red) from Asp-Ala-Asp-Glu-Tyr-Leu-Tyr (PDB accession code: 3I7Z). Water is shown in green or light pink. Vanadium is shown in brown.

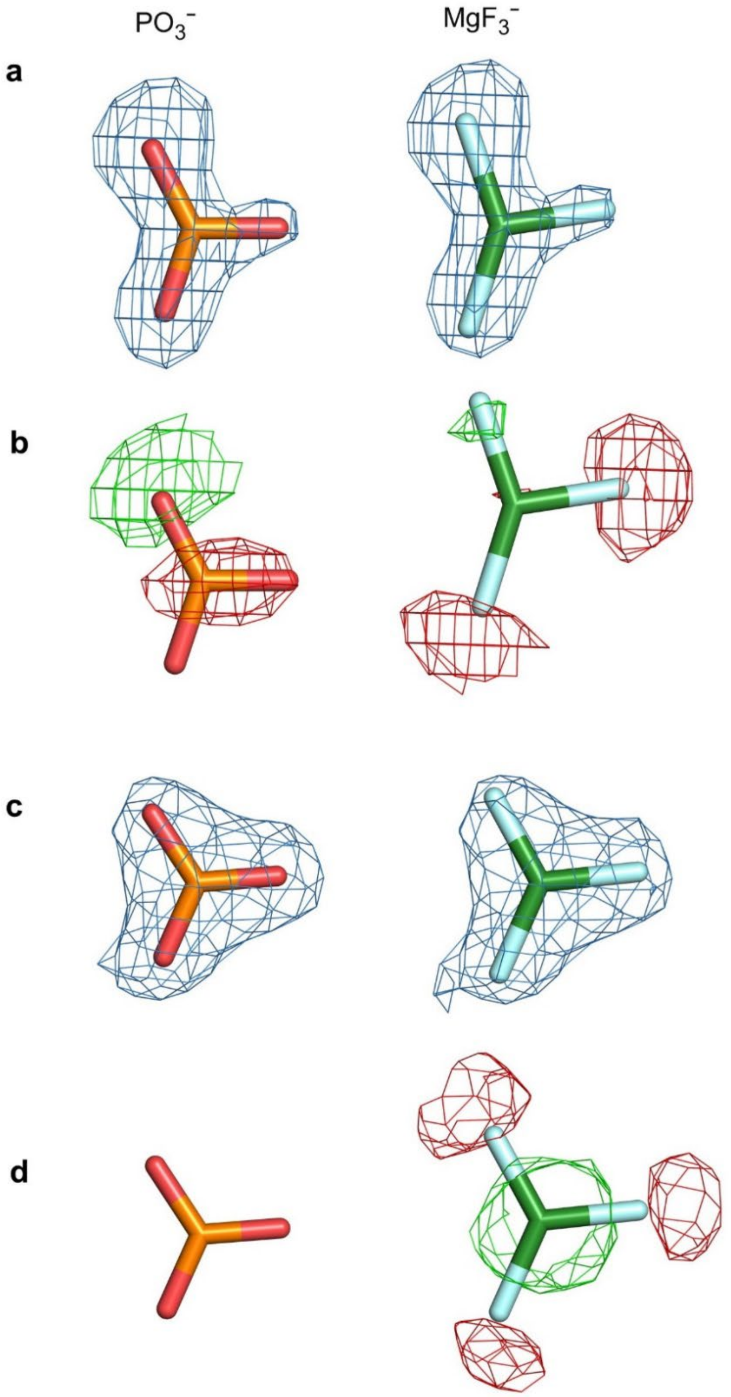

Supplementary Figure 12. Electron density maps modeled on either MgF_3^- or a metaphosphate (PO_3^-). In these stick format graphics, magnesium is colored dark green, fluoride is colored cyan, phosphorus is orange and oxygen is red. The 2mFo-FC maps (blue mesh) are contoured at 1.5σ. The modeled Fo-Fc electron density maps are contoured at 3σ (green mesh) and -3σ (red mesh). Panels **a** and **b** are obtained from experiments with *At*PFA-DSP1$^{49-215}$ (PDB accession code 7MOM). Panels **c** and **d** were obtained from experiments with *At*PFA-DSP1$^{49-215; C150S}$ (PDB accession code 7MOJ).

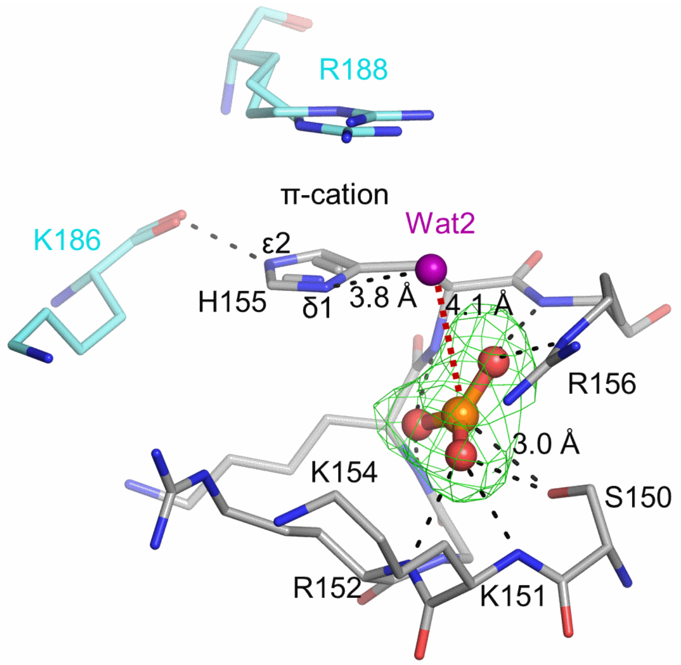

Supplementary Figure 13. A metaphosphate-like intermediate captured in *At*PFA-DSP1$^{49\text{-}215;\ C150S}$. Crystal complexes were generated by soaking 5-InsP$_7$ into the *At*PFA-DSP1$^{49\text{-}215;\ C150S}$ mutant for 2 days at pH 7.2 followed by 1 day at pH 10 (PDB Accession code 7MOJ). Relevant amino acid residues are shown in stick format, the metaphosphate is shown in stick and ball format (phosphorus is orange; oxygens are red), and the water molecule Wat2 is shown as a purple sphere. The distance between Wat2 and phosphorous in Å is indicated by the broken red line. Other distances that are polar interactions are depicted as broken gray lines. The omit Fo-Fc electron density map is contoured at 4σ and shown in green mesh. His155 is shown in N$^{\varepsilon 2}$-protonated τ tautomer state.

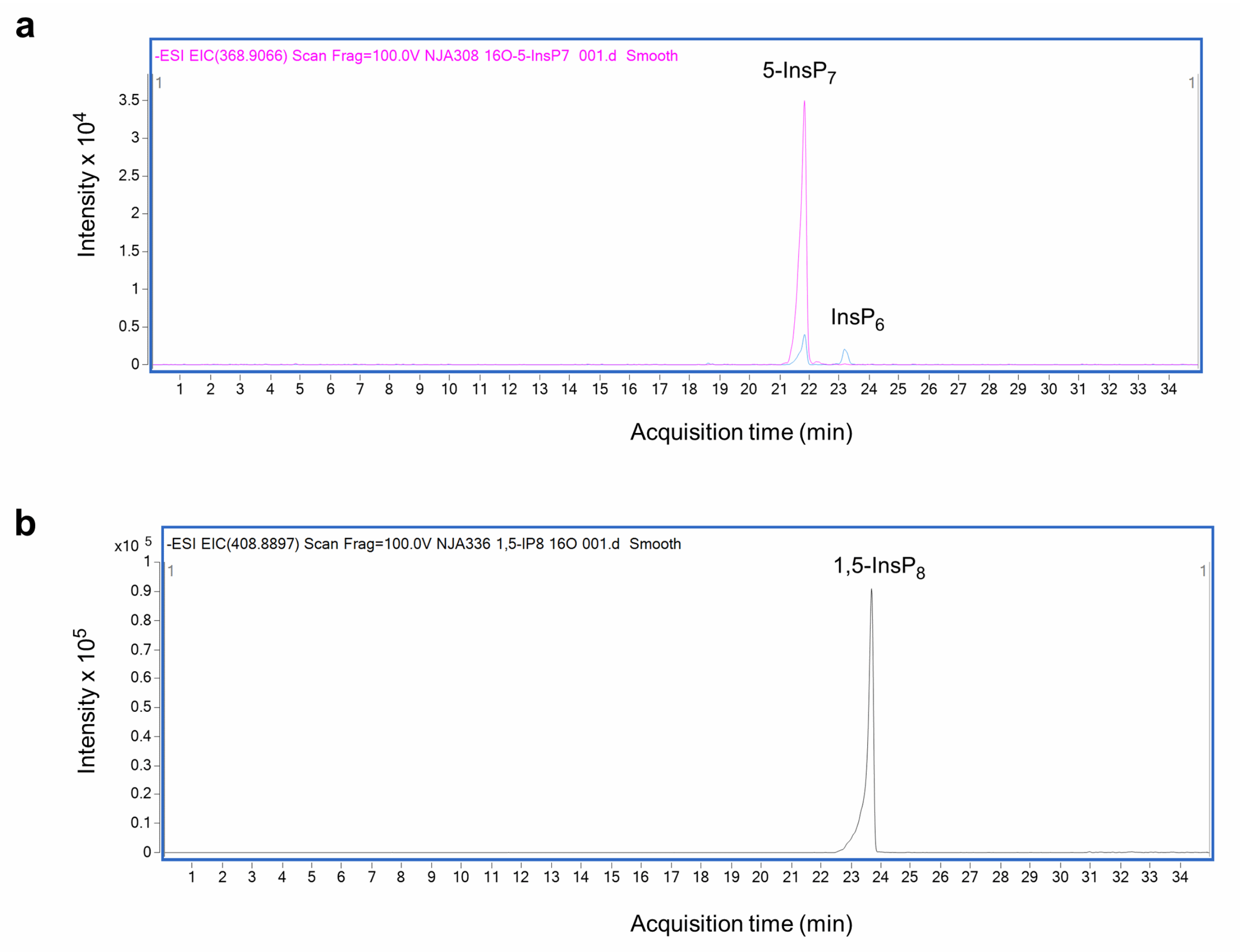

Supplementary Figure 14. Analysis of 5-InsP$_7$ and 1,5-InsP$_8$ by capillary electrophoresis mass spectrometry. Panel **a** shows data for 5-InsP$_7$ (purple trace; retention time = 21.9 min) and < 5% impurities (blue trace) of InsP$_6$ (retention time = 23.1 min) and a neutral loss fragment during ionization of 5-InsP$_7$ (retention time = 21.9 min). Panel b shows data for 1,5-InsP$_8$ (retention time 23.5 min) with <1% impurity.

PDB Accession IDs	7MOD	7MOE	7MOF	7MOG	7MOH
**Protein**	C150S	C150S	C150S	C150S	C150S
**Ligands**	Pi(A)	5-IP_7	6-IP7	5PCF_2Am-IP_5	5-PP-IP4/Pi(B)
**pH**	7.2	7.2	7.2	7.2	7.2
**Space group**	$P2_13$	$P2_13$	$P2_13$	$P2_13$	$P2_13$
**Cell parameters (Å)**	125.36	124.6	124.9	124.65	124.13
**Resolution (Å)**	50.0-1.65 (1.68)	50.0-1.70 (1.73)	50.0-1.95 (1.98)	50-1.80 (1.83)	50.0-1.9 (1.93)
**Rmeas***	0.065 (0.955)	0.068 (0.721)	0.045 (0.737)	0.084 (0.853)	0.049 (0.858)
I/σ I*	31.3 (2.5)	27.8 (2.7)	33.0 (3.1)	23.5 (3.2)	40.9 (2.7)
**Completeness (%)***	100.0 (100.0)	99.7 (99.9)	100.0 (100.0)	100.0 (100.0)	99.9 (100.0)
**Redundancy***	7.8 (7.7)	7.8 (7.6)	7.5 (7.5)	8.2 (8.6)	7.5 (7.5)
**Refinement**					
**Resolution (Å)***	44.4-1.65 (1.69)	41.6-1.7 (1.74)	39.5-1.95 (2.0)	37.6-1.80 (1.85)	37.4-1.90 (1.95)
**Reflections (refinement)**	74329 (5335)	67164 (4901)	45031 (3219)	56694 (4118)	47393 (3417)
**Reflections (R-free)**	3995 (286)	3547 (246)	2391 (168)	3020 (210)	2524 (185)
**Rwork***	0.117 (0.187)	0.120 (0.205)	0.109 (0.155)	0.119 (0.160)	0.117 (0.170)
**Rfree***	0.143 (0.203)	0.149 (0.232)	0.152 (0.226)	0.147 (0.186)	0.148 (0.204)
**No. atoms**					
**Protein**	2777	2659	2566	2569	2552
**Ligands**	10	80	116	82	77
**Solvent**	426	385	337	293	262
**R.m.s. deviations**					
**RMS(bonds Å)**	0.013	0.013	0.015	0.013	0.014
**RMS(angles ◦)**	1.61	1.66	1.74	1.73	1.69
**B-factors ($Å^2$)**					
**Average**	23.68	25.54	30.45	32.23	31.05
**Macromolecules**	20.77	21.87	26.42	29.75	28.32
**Ligands**	15.28	41.81	62.68	58.56	54.44
**Solvent**	42.86	47.49	50.03	46.61	50.76

PDB Accession IDs	7MOI	7MOJ	7MOK	7MOL	7MOM
**Protein**	C150S	C150S	WT	WT	WT
**Ligands/Ion**	Phenyl-P	PO3	Pi(A)	Pi(B)	PO3
**pH**	7.2	10	7.2	7.2	8.0
**Space group**	$P2_13$	$P2_13$	$P2_13$	$P2_13$	$P2_13$
**Cell parameters (Å)**	124.73	125.15	124.45	124.34	124.9
**Resolution (Å)**	50.0-1.80 (1.83)	50.0-1.90 (1.93)	50.0-1.85 (1.88)	50-1.90 (1.93)	50.0-1.70 (1.73)
**Rmeas***	0.040 (0.931)	0.074 (0.871)	0.036 (0.820)	0.97 (0.940)	0.060 (0.969)
I/σ I*	49.6 (2.5)	27.8 (2.7)	43.0 (3.0)	27.5 (2.7)	40.8 (2.6)
**Completeness (%)***	99.9 (99.7)	100.0 (100.0)	100.0 (100.0)	100.0 (100.0)	100.0 (100.0)
**Redundancy***	6.6 (5.4)	7.5 (7.5)	9.4 (9.3)	11.3 (11.7)	10.0 (9.8)
**Refinement**					
**Resolution (Å)***	37.6-1.80 (1.85)	39.4-1.9 (1.95)	39.4-1.85 (1.90)	39.4-1.90 (1.95)	39.53-1.70 (1.74)
**Reflections (refinement)**	56394 (3929)	48867 (3475)	51942 (3753)	48048 (3480)	67721 (4887)
**Reflections (R-free)**	3011 (198)	2601 (192)	2782 (211)	2561 (188)	3627 (270)
**Rwork***	0.123 (0.182)	0.114 (0.170)	0.108 (0.160)	0.115 (0.183)	0.115 (0.189)
**Rfree***	0.149 (0.216)	0.148 (0.215)	0.143 (0.224)	0.157 (0.234)	0.141 (0.200)
**No. atoms**					
**Protein**	2526	2639	2809	2645	2792
**Ligands**	22	8	10	10	8
**Solvent**	301	445	391	365	438
**R.m.s. deviations**					
**RMS(bonds Å)**	0.013	0.014	0.014	0.015	0.013
**RMS(angles ◦)**	1.59	1.71	1.7	1.89	1.74
**B-factors ($Å^2$)**					
**Average**	29.01	30.47	30.41	28.21	23.01
**Macromolecules**	26.93	26.32	27.99	25.47	20.00
**Ligands**	31.02	31.5	27.49	25.07	22.69
**Solvent**	46.33	55.06	47.89	48.12	42.22

Supplementary Table 1. Structural data collection and refinement statistics. *The numbers in parentheses are given for the highest resolution shell.

Atom name	Atomic charge (e)
P	0.3437
O1	-0.7240
O2	-0.5060
O3	-0.7225
O4	-0.7297
HO2	0.3385

Supplementary Table 2. Gaussian charges. Charges were calculated using the CM5 method using gaussian.09.

Atom name	Gaff2 atom type	Partial atomic charge (e)
P	p5	0.3437
O1	oh	-0.7254
O2	o	-0.5060
O3	o	-0.7254
O4	o	-0.7254
HO2	ho	0.3385

Supplementary Table 3. Gaff2 atom type and atomic charge parameters applied to the phosphate ion.

Supplementary Reference

1. Wang, H., Gu, C., Rolfes, R.J., Jessen, H.J. & Shears, S.B. Structural and biochemical characterization of Siw14: A protein-tyrosine phosphatase fold that metabolizes inositol pyrophosphates. *J Biol Chem* **293**, 6905-6914 (2018).
