# Supplementary material for: A structural exposé of noncanonical molecular reactivity within the protein tyrosine phosphatase WPD loop
Source: Nat Commun. 2022 Apr 25;13:2231. doi: 10.1038/s41467-022-29673-y (PMC9038691; doi:10.1038/s41467-022-29673-y)
Supplement: Supplementary file 1 [file 41467_2022_29673_MOESM2_ESM.pdf]

Description of Additional Supplementary Files

File name: Supplementary Movie 1

Description: Molecular dynamics simulation a: phosphate mobility inside the catalytic center of *At*PFA-DSP1

File name: Supplementary Movie 2

Description: Molecular dynamics simulation b: phosphate mobility inside the catalytic center of *At*PFA-DSP1

File name: Supplementary Movie 3

Description: Molecular dynamics simulation c: phosphate mobility inside the catalytic center of *At*PFA-DSP1

File name: Supplementary Movie 4

Description: Molecular dynamics simulation d: phosphate mobility inside the catalytic center of *At*PFA-DSP1
