# Supplementary material for: A structural exposé of noncanonical molecular reactivity within the protein tyrosine phosphatase WPD loop
Source: Nat Commun. 2022 Apr 25;13:2231. doi: 10.1038/s41467-022-29673-y (PMC9038691; doi:10.1038/s41467-022-29673-y)
Supplement: Supplementary file 3 [file 41467_2022_29673_MOESM8_ESM.zip › source data/PDB_Validation_File.zip!/PDB_Validation_File/7MOD_D_1000256158_val-report-full-annotate_P1.pdf]

Full wwPDB X-ray Structure Validation Report (i)

May 3, 2021 – 11:37 AM EDT

PDB ID	:	7MOD
Title	:	Crystal Structure of Arabidopsis thaliana Plant and Fungi Atypical Dual Specificity Phosphatase 1(AtPFA-DSP1) Cys150Ser in Complex with Phosphate in Conformation A (Pi(A))
Deposited on	:	2021-05-01
Resolution	:	1.65 Å(reported)

This is a Full wwPDB X-ray Structure Validation Report.

This report is produced by the wwPDB biocuration pipeline after annotation of the structure.

We welcome your comments at *validation@mail.wwpdb.org*
A user guide is available at
https://www.wwpdb.org/validation/2017/XrayValidationReportHelp
with specific help available everywhere you see the (i) symbol.

The following versions of software and data (see references (i)) were used in the production of this report:

MolProbity	:	4.02b-467
Mogul	:	1.8.5 (274361), CSD as541be (2020)
Xtriage (Phenix)	:	1.13
EDS	:	2.18
buster-report	:	1.1.7 (2018)
Percentile statistics	:	20191225.v01 (using entries in the PDB archive December 25th 2019)
Refmac	:	5.8.0158
CCP4	:	7.0.044 (Gargrove)
Ideal geometry (proteins)	:	Engh & Huber (2001)
Ideal geometry (DNA, RNA)	:	Parkinson et al. (1996)
Validation Pipeline (wwPDB-VP)	:	2.18

CONFIDENTIAL VALIDATION REPORT

1 Overall quality at a glance (i)

The following experimental techniques were used to determine the structure:
X-RAY DIFFRACTION

The reported resolution of this entry is 1.65 Å.

Percentile scores (ranging between 0-100) for global validation metrics of the entry are shown in the following graphic. The table shows the number of entries on which the scores are based.

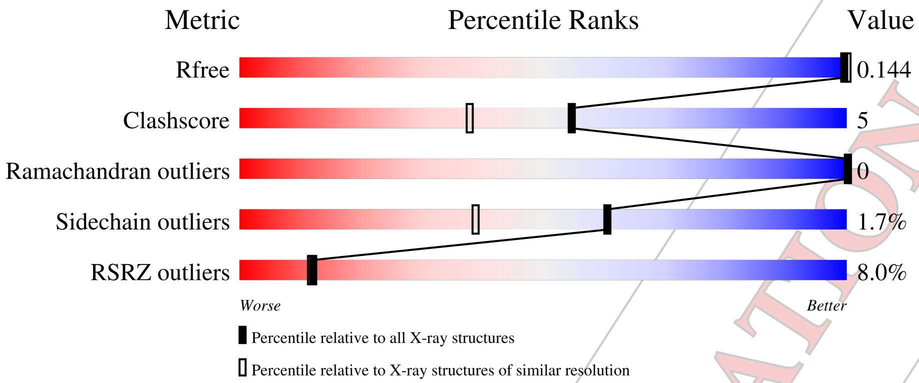

Metric	Whole archive (#Entries)	Similar resolution (#Entries, resolution range(Å))
R_{free}	130704	1827 (1.66-1.66)
Clashscore	141614	1931 (1.66-1.66)
Ramachandran outliers	138981	1891 (1.66-1.66)
Sidechain outliers	138945	1891 (1.66-1.66)
RSRZ outliers	127900	1791 (1.66-1.66)

The table below summarises the geometric issues observed across the polymeric chains and their fit to the electron density. The red, orange, yellow and green segments of the lower bar indicate the fraction of residues that contain outliers for >=3, 2, 1 and 0 types of geometric quality criteria respectively. A grey segment represents the fraction of residues that are not modelled. The numeric value for each fraction is indicated below the corresponding segment, with a dot representing fractions <=5% The upper red bar (where present) indicates the fraction of residues that have poor fit to the electron density. The numeric value is given above the bar.

Mol	Chain	Length	Quality of chain
1	A	171	11% / 85% 11% • •
1	B	171	5% / 86% 8% 6%

CONFIDENTIAL VALIDATION REPORT

2 Entry composition (i)

There are 3 unique types of molecules in this entry. The entry contains 3185 atoms, of which 0 are hydrogens and 0 are deuteriums.

In the tables below, the ZeroOcc column contains the number of atoms modelled with zero occupancy, the AltConf column contains the number of residues with at least one atom in alternate conformation and the Trace column contains the number of residues modelled with at most 2 atoms.

- Molecule 1 is a protein called Tyrosine-protein phosphatase DSP1.

Mol	Chain	Residues	Atoms					ZeroOcc	AltConf	Trace
1	A	164	Total 1405	C 902	N 251	O 242	S 10	0	9	0
1	B	161	Total 1354	C 870	N 239	O 235	S 10	0	7	0

There are 10 discrepancies between the modelled and reference sequences:

Chain	Residue	Modelled	Actual	Comment	Reference
A	45	GLY	-	expression tag	UNP Q9ZVN4
A	46	SER	-	expression tag	UNP Q9ZVN4
A	47	PHE	-	expression tag	UNP Q9ZVN4
A	48	THR	-	expression tag	UNP Q9ZVN4
A	150	SER	CYS	engineered mutation	UNP Q9ZVN4
B	45	GLY	-	expression tag	UNP Q9ZVN4
B	46	SER	-	expression tag	UNP Q9ZVN4
B	47	PHE	-	expression tag	UNP Q9ZVN4
B	48	THR	-	expression tag	UNP Q9ZVN4
B	150	SER	CYS	engineered mutation	UNP Q9ZVN4

- Molecule 2 is PHOSPHATE ION (three-letter code: PO4) (formula: O_4P) (labeled as "Ligand of Interest" by depositor).

CONFIDENTIAL VALIDATION REPORT

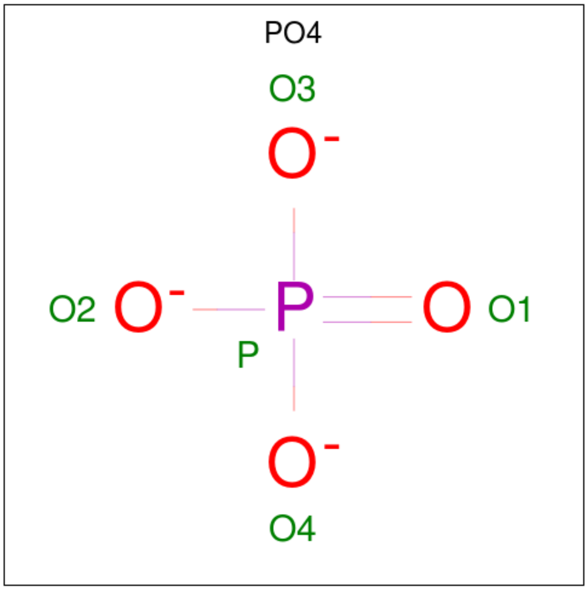

Mol	Chain	Residues	Atoms	ZeroOcc	AltConf
2	A	1	Total O P<br>5 4 1	0	0
2	B	1	Total O P<br>5 4 1	0	0

- Molecule 3 is water.

Mol	Chain	Residues	Atoms	ZeroOcc	AltConf
3	A	200	Total O<br>200 200	0	0
3	B	216	Total O<br>216 216	0	0

CONFIDENTIAL VALIDATION REPORT

3 Residue-property plots (i)

These plots are drawn for all protein, RNA, DNA and oligosaccharide chains in the entry. The first graphic for a chain summarises the proportions of the various outlier classes displayed in the second graphic. The second graphic shows the sequence view annotated by issues in geometry and electron density. Residues are color-coded according to the number of geometric quality criteria for which they contain at least one outlier: green = 0, yellow = 1, orange = 2 and red = 3 or more. A red dot above a residue indicates a poor fit to the electron density (RSRZ > 2). Stretches of 2 or more consecutive residues without any outlier are shown as a green connector. Residues present in the sample, but not in the model, are shown in grey.

- Molecule 1: Tyrosine-protein phosphatase DSP1

Chain A:

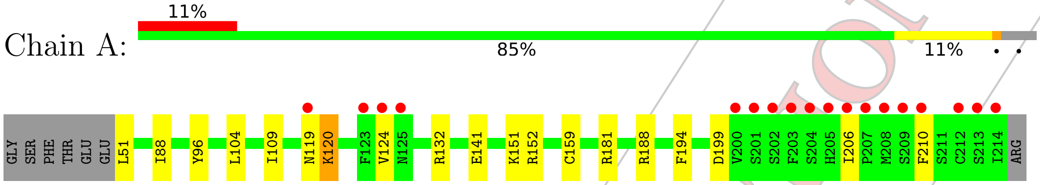

- Molecule 1: Tyrosine-protein phosphatase DSP1

Chain B:

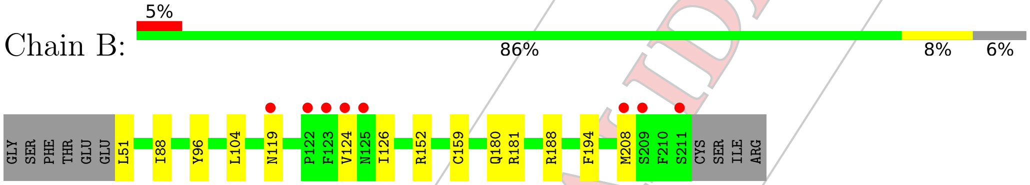

CONFIDENTIAL VALIDATION REPORT

4 Data and refinement statistics (i)

Property	Value	Source
Space group	P 21 3	Depositor
Cell constants<br>a, b, c, α, β, γ	125.36Å 125.36Å 125.36Å<br>90.00° 90.00° 90.00°	Depositor
Resolution (Å)	44.36 – 1.65<br>44.32 – 1.65	Depositor<br>EDS
% Data completeness<br>(in resolution range)	99.6 (44.36-1.65)<br>99.6 (44.32-1.65)	Depositor<br>EDS
R_{merge}	(Not available)	Depositor
R_{sym}	(Not available)	Depositor
$< I/\sigma(I) >$ [1]	2.84 (at 1.65Å)	Xtriage
Refinement program	REFMAC 5.8.0258	Depositor
R, R_{free}	0.117 , 0.143<br>0.119 , 0.144	Depositor<br>DCC
R_{free} test set	3995 reflections (5.10%)	wwPDB-VP
Wilson B-factor (Å^2)	13.4	Xtriage
Anisotropy	0.000	Xtriage
Bulk solvent k_{sol}(e/Å^3), B_{sol}(Å^2)	0.38 , 59.4	EDS
L-test for twinning[2]	$< \|L\| > = 0.51$, $< L^2 > = 0.34$	Xtriage
Estimated twinning fraction	0.022 for l,-k,h	Xtriage
F_o,F_c correlation	0.97	EDS
Total number of atoms	3185	wwPDB-VP
Average B, all atoms (Å^2)	23.0	wwPDB-VP

Xtriage's analysis on translational NCS is as follows: *The largest off-origin peak in the Patterson function is 2.96% of the height of the origin peak. No significant pseudotranslation is detected.*

[1] Intensities estimated from amplitudes.

[2] Theoretical values of $< |L| >$, $< L^2 >$ for acentric reflections are 0.5, 0.333 respectively for untwinned datasets, and 0.375, 0.2 for perfectly twinned datasets.

CONFIDENTIAL VALIDATION REPORT

5 Model quality (i)

5.1 Standard geometry (i)

Bond lengths and bond angles in the following residue types are not validated in this section: PO4

The Z score for a bond length (or angle) is the number of standard deviations the observed value is removed from the expected value. A bond length (or angle) with $|Z| > 5$ is considered an outlier worth inspection. RMSZ is the root-mean-square of all Z scores of the bond lengths (or angles).

Mol	Chain	Bond lengths		Bond angles	
		RMSZ	$\#|Z| > 5$	RMSZ	$\#|Z| > 5$
1	A	0.63	0/1441	0.78	2/1939 (0.1%)
1	B	0.64	0/1389	0.74	2/1870 (0.1%)
All	All	0.63	0/2830	0.76	4/3809 (0.1%)

There are no bond length outliers.

All (4) bond angle outliers are listed below:

Mol	Chain	Res	Type	Atoms	Z	Observed(^{o})	Ideal(^{o})
1	A	181	ARG	NE-CZ-NH2	-5.35	117.63	120.30
1	A	181	ARG	NE-CZ-NH1	5.20	122.90	120.30
1	B	181	ARG	NE-CZ-NH1	5.11	122.86	120.30
1	B	181	ARG	NE-CZ-NH2	-5.01	117.79	120.30

There are no chirality outliers.

There are no planarity outliers.

5.2 Too-close contacts (i)

In the following table, the Non-H and H(model) columns list the number of non-hydrogen atoms and hydrogen atoms in the chain respectively. The H(added) column lists the number of hydrogen atoms added and optimized by MolProbity. The Clashes column lists the number of clashes within the asymmetric unit, whereas Symm-Clashes lists symmetry-related clashes.

Mol	Chain	Non-H	H(model)	H(added)	Clashes	Symm-Clashes
1	A	1405	0	1406	17	0
1	B	1354	0	1350	12	0
2	A	5	0	0	0	0
2	B	5	0	0	0	0
3	A	200	0	0	6	0

Continued on next page...

CONFIDENTIAL VALIDATION REPORT

Continued from previous page...

Mol	Chain	Non-H	H(model)	H(added)	Clashes	Symm-Clashes
3	B	216	0	0	8	0
All	All	3185	0	2756	29	0

The all-atom clashscore is defined as the number of clashes found per 1000 atoms (including hydrogen atoms). The all-atom clashscore for this structure is 5.

All (29) close contacts within the same asymmetric unit are listed below, sorted by their clash magnitude.

Atom-1	Atom-2	Interatomic distance (Å)	Clash overlap (Å)
1:A:159[B]:CYS:SG	1:A:194:PHE:CD2	2.71	0.83
1:B:124:VAL:HG11	3:B:629:HOH:O	1.88	0.74
1:A:88:ILE:HD12	1:A:104[A]:LEU:HD21	1.74	0.70
1:B:124:VAL:CG1	3:B:629:HOH:O	2.41	0.69
1:A:124:VAL:HG11	3:A:642:HOH:O	1.93	0.68
1:B:159[B]:CYS:SG	1:B:194:PHE:CD2	2.87	0.67
1:A:141:GLU:HG2	1:A:210:PHE:HB3	1.77	0.67
1:B:51:LEU:N	3:B:603:HOH:O	2.28	0.66
1:A:51:LEU:N	3:A:602:HOH:O	2.30	0.64
1:B:152:ARG:CD	3:B:622:HOH:O	2.44	0.63
1:B:152:ARG:HD3	3:B:622:HOH:O	1.99	0.63
1:B:119:ASN:O	1:B:188[B]:ARG:NH2	2.33	0.62
1:A:141:GLU:CD	1:A:210:PHE:HB3	2.20	0.62
1:A:119:ASN:O	1:A:188[B]:ARG:NH2	2.34	0.60
1:B:180[A]:GLN:NE2	3:B:606:HOH:O	2.35	0.59
1:A:152:ARG:HD2	3:A:759:HOH:O	2.03	0.58
1:A:141:GLU:CG	1:A:210:PHE:HB3	2.34	0.56
1:A:120:LYS:HD3	1:A:188[A]:ARG:HH11	1.70	0.56
1:B:180[B]:GLN:CD	3:B:604:HOH:O	2.47	0.53
1:A:124:VAL:CG1	3:A:642:HOH:O	2.51	0.52
1:A:51:LEU:N	3:A:607:HOH:O	2.44	0.51
1:A:104[B]:LEU:HG	1:A:109:ILE:O	2.12	0.50
1:A:159[B]:CYS:SG	1:A:194:PHE:CE2	3.07	0.47
1:B:88:ILE:HD12	1:B:104:LEU:HD21	1.96	0.47
1:A:151:LYS:NZ	3:A:612:HOH:O	2.51	0.44
1:A:132:ARG:NH2	1:A:199:ASP:O	2.41	0.43
1:A:88:ILE:HD12	1:A:104[A]:LEU:CD2	2.47	0.42
1:B:126:ILE:HD11	3:B:629:HOH:O	2.20	0.42
1:B:159[B]:CYS:SG	1:B:194:PHE:CE2	3.14	0.41

There are no symmetry-related clashes.

CONFIDENTIAL VALIDATION REPORT

5.3 Torsion angles (i)

5.3.1 Protein backbone (i)

In the following table, the Percentiles column shows the percent Ramachandran outliers of the chain as a percentile score with respect to all X-ray entries followed by that with respect to entries of similar resolution.

The Analysed column shows the number of residues for which the backbone conformation was analysed, and the total number of residues.

Mol	Chain	Analysed	Favoured	Allowed	Outliers	Percentiles	
1	A	172/171 (101%)	167 (97%)	5 (3%)	0	100	100
1	B	166/171 (97%)	162 (98%)	4 (2%)	0	100	100
All	All	338/342 (99%)	329 (97%)	9 (3%)	0	100	100

There are no Ramachandran outliers to report.

5.3.2 Protein sidechains (i)

In the following table, the Percentiles column shows the percent sidechain outliers of the chain as a percentile score with respect to all X-ray entries followed by that with respect to entries of similar resolution.

The Analysed column shows the number of residues for which the sidechain conformation was analysed, and the total number of residues.

Mol	Chain	Analysed	Rotameric	Outliers	Percentiles	
1	A	159/155 (103%)	156 (98%)	3 (2%)	57	34
1	B	153/155 (99%)	151 (99%)	2 (1%)	69	50
All	All	312/310 (101%)	307 (98%)	5 (2%)	60	41

All (5) residues with a non-rotameric sidechain are listed below:

Mol	Chain	Res	Type
1	A	96	TYR
1	A	120	LYS
1	A	206	ILE
1	B	96	TYR
1	B	208	MET

Sometimes sidechains can be flipped to improve hydrogen bonding and reduce clashes. All (1) such sidechains are listed below:

CONFIDENTIAL VALIDATION REPORT

Mol	Chain	Res	Type
1	B	81	GLN

5.3.3 RNA (i)

There are no RNA molecules in this entry.

5.4 Non-standard residues in protein, DNA, RNA chains (i)

There are no non-standard protein/DNA/RNA residues in this entry.

5.5 Carbohydrates (i)

There are no monosaccharides in this entry.

5.6 Ligand geometry (i)

2 ligands are modelled in this entry.

In the following table, the Counts columns list the number of bonds (or angles) for which Mogul statistics could be retrieved, the number of bonds (or angles) that are observed in the model and the number of bonds (or angles) that are defined in the Chemical Component Dictionary. The Link column lists molecule types, if any, to which the group is linked. The Z score for a bond length (or angle) is the number of standard deviations the observed value is removed from the expected value. A bond length (or angle) with $|Z| > 2$ is considered an outlier worth inspection. RMSZ is the root-mean-square of all Z scores of the bond lengths (or angles).

Mol	Type	Chain	Res	Link	Bond lengths			Bond angles		
					Counts	RMSZ	$\#\|Z\| > 2$	Counts	RMSZ	$\#\|Z\| > 2$
2	PO4	A	501	-	4,4,4	1.12	0	6,6,6	1.04	0
2	PO4	B	501	-	4,4,4	1.06	0	6,6,6	0.43	0

There are no bond length outliers.

There are no bond angle outliers.

There are no chirality outliers.

There are no torsion outliers.

There are no ring outliers.

No monomer is involved in short contacts.

The following is a two-dimensional graphical depiction of Mogul quality analysis of bond lengths, bond angles, torsion angles, and ring geometry for all instances of the Ligand of Interest. In

CONFIDENTIAL VALIDATION REPORT

addition, ligands with molecular weight > 250 and outliers as shown on the validation Tables will also be included. For torsion angles, if less then 5% of the Mogul distribution of torsion angles is within 10 degrees of the torsion angle in question, then that torsion angle is considered an outlier. Any bond that is central to one or more torsion angles identified as an outlier by Mogul will be highlighted in the graph. For rings, the root-mean-square deviation (RMSD) between the ring in question and similar rings identified by Mogul is calculated over all ring torsion angles. If the average RMSD is greater than 60 degrees and the minimal RMSD between the ring in question and any Mogul-identified rings is also greater than 60 degrees, then that ring is considered an outlier. The outliers are highlighted in purple. The color gray indicates Mogul did not find sufficient equivalents in the CSD to analyse the geometry.

**Ligand PO4 A 501**	
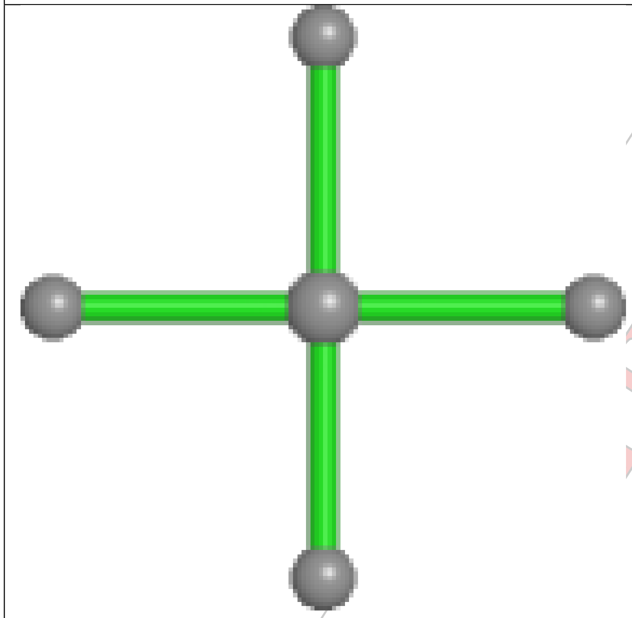 **Bond lengths**	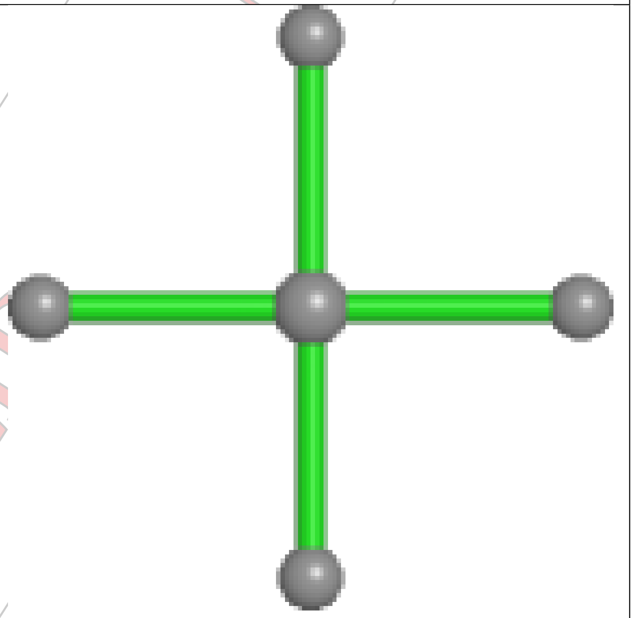 **Bond angles**
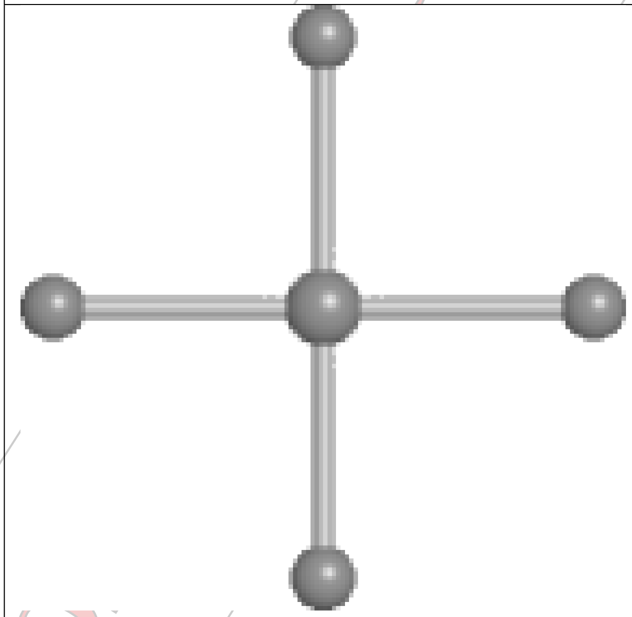 **Torsions**	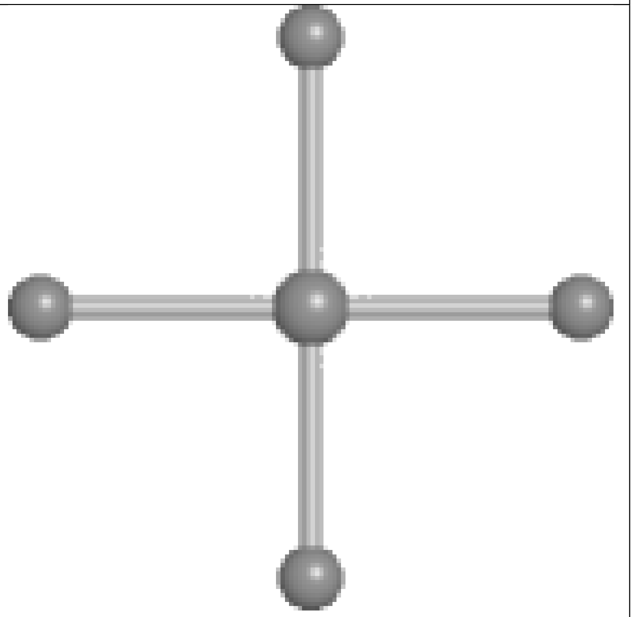 **Rings**

Ligand PO4 B 501

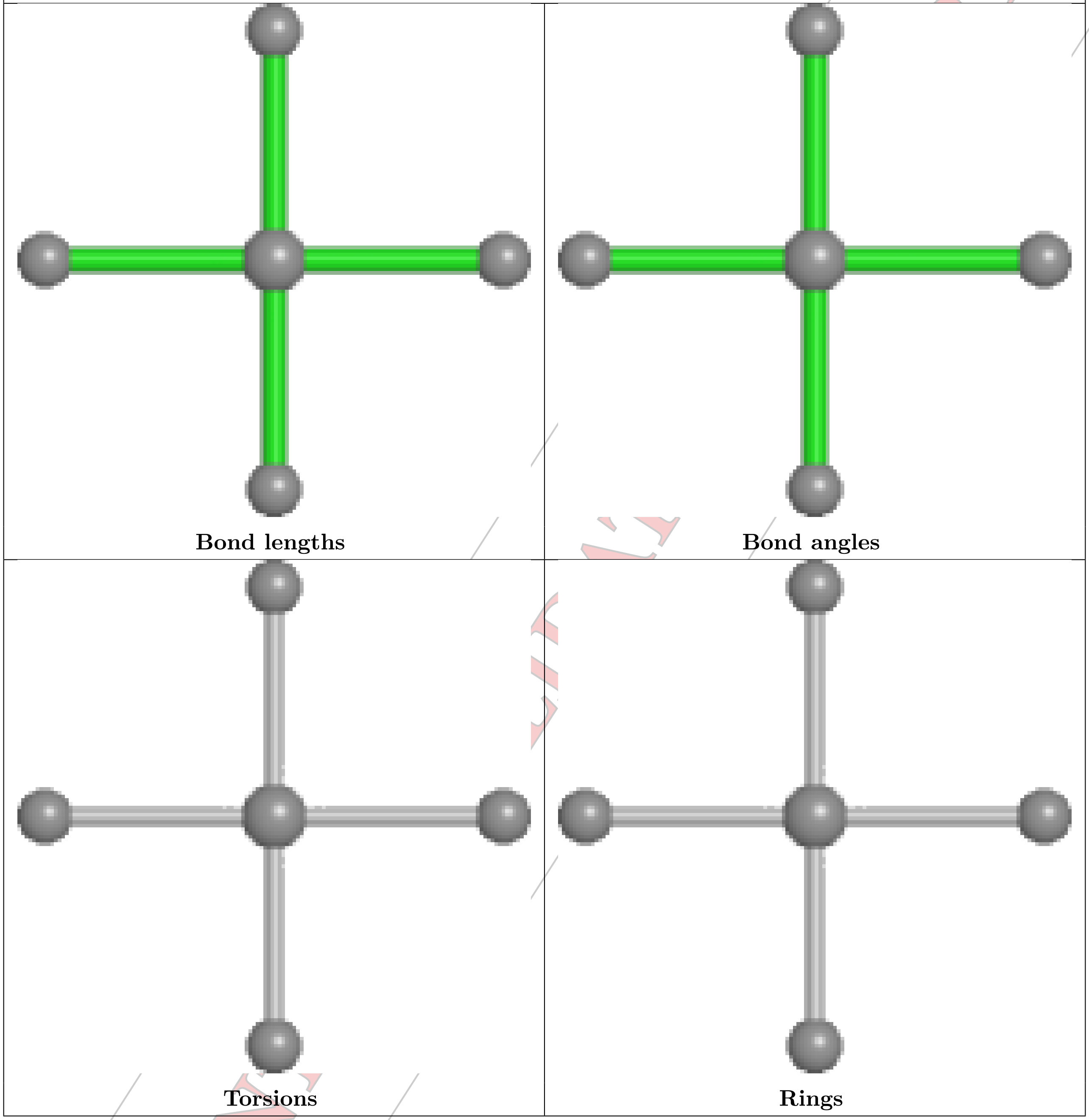

5.7 Other polymers (i)

There are no such residues in this entry.

5.8 Polymer linkage issues (i)

There are no chain breaks in this entry.

6 Fit of model and data (i)

6.1 Protein, DNA and RNA chains (i)

In the following table, the column labelled '#RSRZ> 2' contains the number (and percentage) of RSRZ outliers, followed by percent RSRZ outliers for the chain as percentile scores relative to all X-ray entries and entries of similar resolution. The OWAB column contains the minimum, median, 95th percentile and maximum values of the occupancy-weighted average B-factor per residue. The column labelled 'Q< 0.9' lists the number of (and percentage) of residues with an average occupancy less than 0.9.

Mol	Chain	Analysed	<RSRZ>	#RSRZ>2			OWAB(Å^2)	Q<0.9
1	A	164/171 (95%)	0.18	18 (10%)	5	4	7, 16, 51, 107	0
1	B	161/171 (94%)	-0.22	8 (4%)	28	27	7, 15, 40, 51	0
All	All	325/342 (95%)	-0.02	26 (8%)	12	12	7, 15, 46, 107	0

All (26) RSRZ outliers are listed below:

Mol	Chain	Res	Type	RSRZ
1	A	206	ILE	7.6
1	A	207	PRO	7.4
1	A	204	SER	6.4
1	A	212	CYS	6.3
1	A	214	ILE	5.8
1	A	213	SER	5.5
1	A	205	HIS	5.4
1	B	123	PHE	4.8
1	B	124	VAL	4.5
1	A	209	SER	4.2
1	A	123	PHE	4.1
1	A	202	SER	4.0
1	A	210	PHE	3.5
1	A	201	SER	3.4
1	A	208	MET	3.2
1	A	200	VAL	3.0
1	B	209	SER	2.9
1	B	211	SER	2.8
1	B	208	MET	2.6
1	B	125	ASN	2.6
1	A	203	PHE	2.3
1	A	119	ASN	2.3
1	B	122	PRO	2.1
1	A	124	VAL	2.1

Continued on next page...

CONFIDENTIAL VALIDATION REPORT

Continued from previous page...

Mol	Chain	Res	Type	RSRZ
1	B	119	ASN	2.0
1	A	125	ASN	2.0

6.2 Non-standard residues in protein, DNA, RNA chains (i)

There are no non-standard protein/DNA/RNA residues in this entry.

6.3 Carbohydrates (i)

There are no monosaccharides in this entry.

6.4 Ligands (i)

In the following table, the Atoms column lists the number of modelled atoms in the group and the number defined in the chemical component dictionary. The B-factors column lists the minimum, median, 95th percentile and maximum values of B factors of atoms in the group. The column labelled 'Q< 0.9' lists the number of atoms with occupancy less than 0.9.

Mol	Type	Chain	Res	Atoms	RSCC	RSR	B-factors(Å^2)	Q<0.9
2	PO4	A	501	5/5	1.00	0.05	13,13,19,20	0
2	PO4	B	501	5/5	1.00	0.04	11,11,16,19	0

The following is a graphical depiction of the model fit to experimental electron density of all instances of the Ligand of Interest. In addition, ligands with molecular weight > 250 and outliers as shown on the geometry validation Tables will also be included. Each fit is shown from different orientation to approximate a three-dimensional view.

CONFIDENTIAL VALIDATION REPORT

Electron density around PO4 A 501:

$2mF_o$-DF_c (at 0.7 rmsd) in gray
mF_o-DF_c (at 3 rmsd) in purple (negative)
and green (positive)

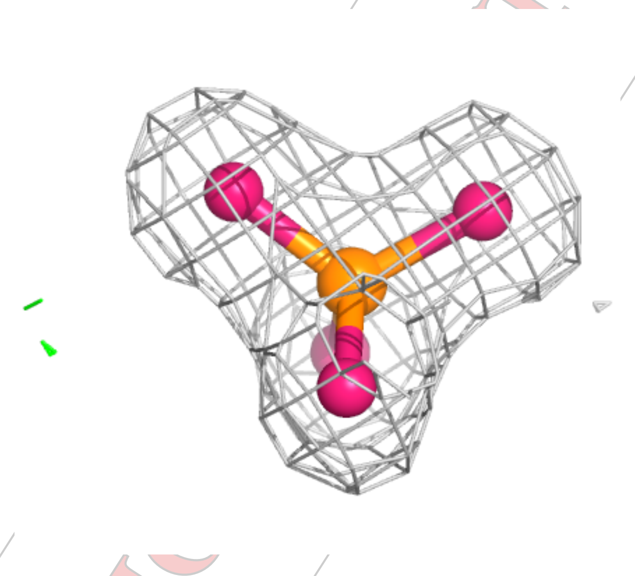

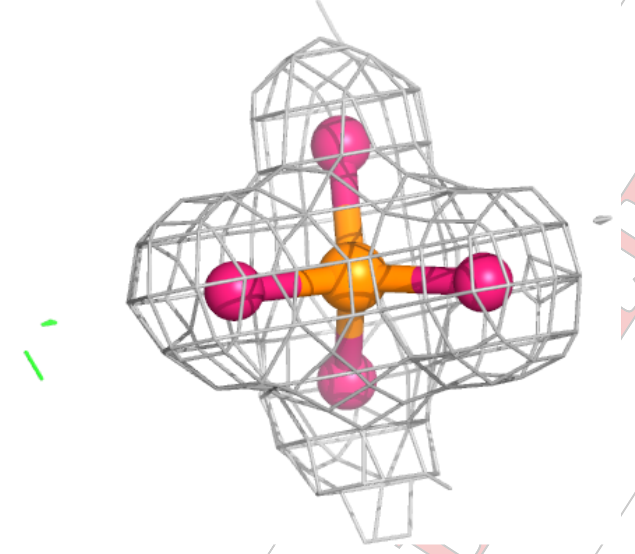

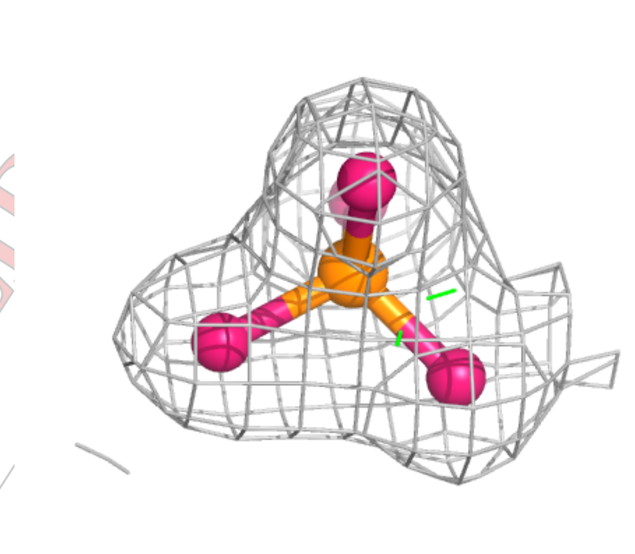

CONFIDENTIAL

Electron density around PO4 B 501:

$2mF_o$-DF_c (at 0.7 rmsd) in gray
mF_o-DF_c (at 3 rmsd) in purple (negative)
and green (positive)

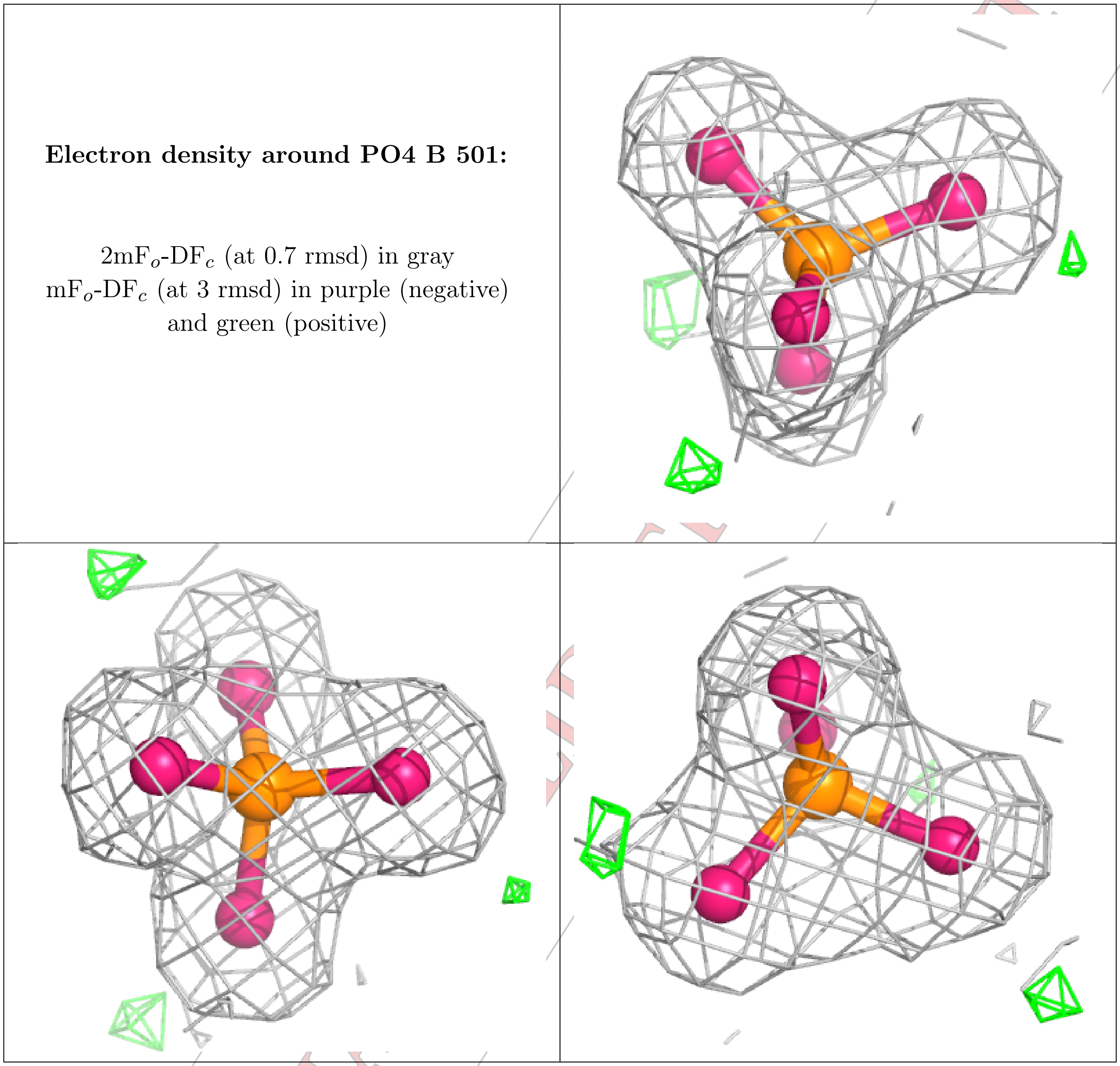

6.5 Other polymers ⓘ

There are no such residues in this entry.

CONFIDENTIAL
