# Supplementary material for: A structural exposé of noncanonical molecular reactivity within the protein tyrosine phosphatase WPD loop
Source: Nat Commun. 2022 Apr 25;13:2231. doi: 10.1038/s41467-022-29673-y (PMC9038691; doi:10.1038/s41467-022-29673-y)
Supplement: Supplementary file 3 [file 41467_2022_29673_MOESM8_ESM.zip › source data/PDB_Validation_File.zip!/PDB_Validation_File/7MOG_D_1000256183_val-report-full-annotate_P1.pdf]

Full wwPDB X-ray Structure Validation Report (i)

May 3, 2021 – 11:45 AM EDT

PDB ID	:	7MOG
Title	:	Crystal Structure of Arabidopsis thaliana Plant and Fungi Atypical Dual Specificity Phosphatase 1(AtPFA-DSP1) Cys150Ser in complex with 5-PCF2 Am-InsP5, an analogue of 5-InsP7
Deposited on	:	2021-05-01
Resolution	:	1.80 Å(reported)

This is a Full wwPDB X-ray Structure Validation Report.

This report is produced by the wwPDB biocuration pipeline after annotation of the structure.

We welcome your comments at *validation@mail.wwpdb.org*
A user guide is available at
https://www.wwpdb.org/validation/2017/XrayValidationReportHelp
with specific help available everywhere you see the (i) symbol.

CONFIDENTIAL VALIDATION REPORT

The following versions of software and data (see references (i)) were used in the production of this report:

MolProbity	:	4.02b-467
Mogul	:	1.8.5 (274361), CSD as541be (2020)
Xtriage (Phenix)	:	1.13
EDS	:	2.18
buster-report	:	1.1.7 (2018)
Percentile statistics	:	20191225.v01 (using entries in the PDB archive December 25th 2019)
Refmac	:	5.8.0158
CCP4	:	7.0.044 (Gargrove)
Ideal geometry (proteins)	:	Engh & Huber (2001)
Ideal geometry (DNA, RNA)	:	Parkinson et al. (1996)
Validation Pipeline (wwPDB-VP)	:	2.18

1 Overall quality at a glance ⓘ

The following experimental techniques were used to determine the structure:
X-RAY DIFFRACTION

The reported resolution of this entry is 1.80 Å.

Percentile scores (ranging between 0-100) for global validation metrics of the entry are shown in the following graphic. The table shows the number of entries on which the scores are based.

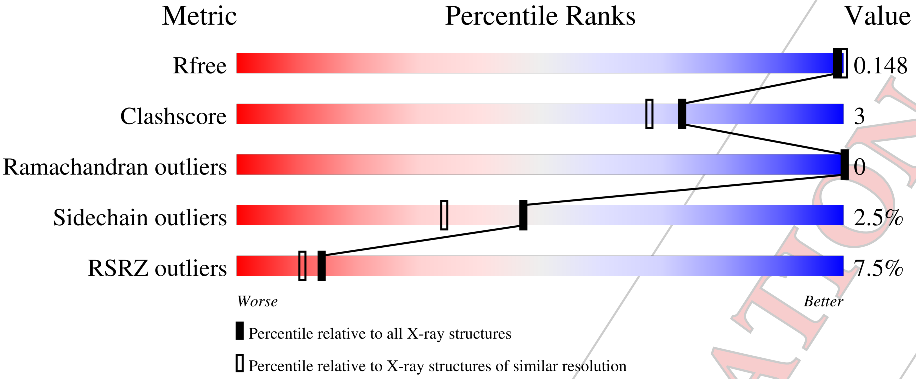

Metric	Whole archive (#Entries)	Similar resolution (#Entries, resolution range(Å))
R_{free}	130704	5950 (1.80-1.80)
Clashscore	141614	6793 (1.80-1.80)
Ramachandran outliers	138981	6697 (1.80-1.80)
Sidechain outliers	138945	6696 (1.80-1.80)
RSRZ outliers	127900	5850 (1.80-1.80)

The table below summarises the geometric issues observed across the polymeric chains and their fit to the electron density. The red, orange, yellow and green segments of the lower bar indicate the fraction of residues that contain outliers for >=3, 2, 1 and 0 types of geometric quality criteria respectively. A grey segment represents the fraction of residues that are not modelled. The numeric value for each fraction is indicated below the corresponding segment, with a dot representing fractions <=5% The upper red bar (where present) indicates the fraction of residues that have poor fit to the electron density. The numeric value is given above the bar.

Mol	Chain	Length	Quality of chain
1	A	171	6% / 82% 8% • 9%
1	B	171	7% / 82% 6% • 11%

CONFIDENTIAL VALIDATION REPORT

2 Entry composition (i)

There are 3 unique types of molecules in this entry. The entry contains 2944 atoms, of which 0 are hydrogens and 0 are deuteriums.

In the tables below, the ZeroOcc column contains the number of atoms modelled with zero occupancy, the AltConf column contains the number of residues with at least one atom in alternate conformation and the Trace column contains the number of residues modelled with at most 2 atoms.

- Molecule 1 is a protein called Tyrosine-protein phosphatase DSP1.

Mol	Chain	Residues	Atoms					ZeroOcc	AltConf	Trace
1	A	155	Total<br>1296	C<br>832	N<br>227	O<br>229	S<br>8	0	6	0
1	B	152	Total<br>1273	C<br>820	N<br>223	O<br>221	S<br>9	0	4	0

There are 10 discrepancies between the modelled and reference sequences:

Chain	Residue	Modelled	Actual	Comment	Reference
A	45	GLY	-	expression tag	UNP Q9ZVN4
A	46	SER	-	expression tag	UNP Q9ZVN4
A	47	PHE	-	expression tag	UNP Q9ZVN4
A	48	THR	-	expression tag	UNP Q9ZVN4
A	150	SER	CYS	engineered mutation	UNP Q9ZVN4
B	45	GLY	-	expression tag	UNP Q9ZVN4
B	46	SER	-	expression tag	UNP Q9ZVN4
B	47	PHE	-	expression tag	UNP Q9ZVN4
B	48	THR	-	expression tag	UNP Q9ZVN4
B	150	SER	CYS	engineered mutation	UNP Q9ZVN4

- Molecule 2 is (1,1-difluoro-2-oxo-2-{[(1s,2R,3S,4s,5R,6S)-2,3,4,5,6-pentakis(phosphonooxy)cyclohexyl]amino}ethyl)phosphonic acid (three-letter code: KDJ) (formula: $C_8H_{19}F_2NO_{24}P_6$) (labeled as "Ligand of Interest" by depositor).

CONFIDENTIAL VALIDATION REPORT

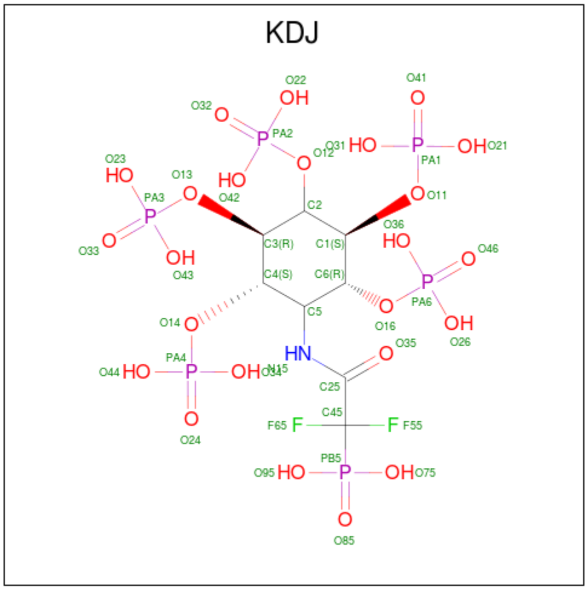

Mol	Chain	Residues	Atoms	ZeroOcc	AltConf
2	A	1	Total C F N O P<br>41 8 2 1 24 6	0	0
2	B	1	Total C F N O P<br>41 8 2 1 24 6	0	0

- Molecule 3 is water.

Mol	Chain	Residues	Atoms	ZeroOcc	AltConf
3	A	150	Total O<br>150 150	0	0
3	B	143	Total O<br>143 143	0	0

CONFIDENTIAL VALIDATION REPORT

3 Residue-property plots (i)

These plots are drawn for all protein, RNA, DNA and oligosaccharide chains in the entry. The first graphic for a chain summarises the proportions of the various outlier classes displayed in the second graphic. The second graphic shows the sequence view annotated by issues in geometry and electron density. Residues are color-coded according to the number of geometric quality criteria for which they contain at least one outlier: green = 0, yellow = 1, orange = 2 and red = 3 or more. A red dot above a residue indicates a poor fit to the electron density (RSRZ > 2). Stretches of 2 or more consecutive residues without any outlier are shown as a green connector. Residues present in the sample, but not in the model, are shown in grey.

• Molecule 1: Tyrosine-protein phosphatase DSP1

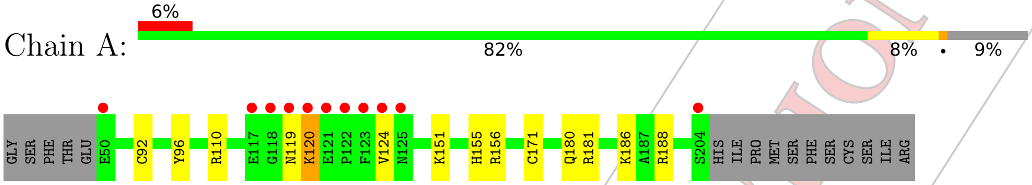

• Molecule 1: Tyrosine-protein phosphatase DSP1

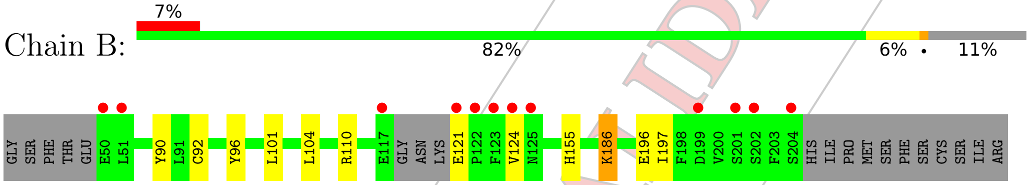

CONFIDENTIAL VALIDATION REPORT

4 Data and refinement statistics (i)

Property	Value	Source
Space group	P 21 3	Depositor
Cell constants<br>a, b, c, α, β, γ	124.65Å 124.65Å 124.65Å<br>90.00° 90.00° 90.00°	Depositor
Resolution (Å)	37.61 – 1.80<br>37.58 – 1.80	Depositor<br>EDS
% Data completeness<br>(in resolution range)	99.7 (37.61-1.80)<br>99.7 (37.58-1.80)	Depositor<br>EDS
R_{merge}	(Not available)	Depositor
R_{sym}	(Not available)	Depositor
$< I/\sigma(I) >$ [1]	4.56 (at 1.79Å)	Xtriage
Refinement program	REFMAC 5.8.0258	Depositor
R, R_{free}	0.119 , 0.147<br>0.120 , 0.148	Depositor<br>DCC
R_{free} test set	3022 reflections (5.06%)	wwPDB-VP
Wilson B-factor (Å^2)	18.2	Xtriage
Anisotropy	0.000	Xtriage
Bulk solvent k_{sol}(e/Å^3), B_{sol}(Å^2)	0.38 , 56.5	EDS
L-test for twinning[2]	$< \|L\| >$ = 0.50, $< L^2 >$ = 0.33	Xtriage
Estimated twinning fraction	0.027 for l,-k,h	Xtriage
F_o,F_c correlation	0.97	EDS
Total number of atoms	2944	wwPDB-VP
Average B, all atoms (Å^2)	32.0	wwPDB-VP

Xtriage's analysis on translational NCS is as follows: *The largest off-origin peak in the Patterson function is 2.55% of the height of the origin peak. No significant pseudotranslation is detected.*

[1] Intensities estimated from amplitudes.

[2] Theoretical values of $< |L| >$, $< L^2 >$ for acentric reflections are 0.5, 0.333 respectively for untwinned datasets, and 0.375, 0.2 for perfectly twinned datasets.

CONFIDENTIAL VALIDATION REPORT

5 Model quality (i)

5.1 Standard geometry (i)

Bond lengths and bond angles in the following residue types are not validated in this section: KDJ

The Z score for a bond length (or angle) is the number of standard deviations the observed value is removed from the expected value. A bond length (or angle) with $|Z| > 5$ is considered an outlier worth inspection. RMSZ is the root-mean-square of all Z scores of the bond lengths (or angles).

Mol	Chain	Bond lengths		Bond angles	
		RMSZ	#$\|Z\|$ >5	RMSZ	#$\|Z\|$ >5
1	A	0.68	0/1327	0.82	1/1785 (0.1%)
1	B	0.66	0/1304	0.79	0/1754
All	All	0.67	0/2631	0.81	1/3539 (0.0%)

There are no bond length outliers.

All (1) bond angle outliers are listed below:

Mol	Chain	Res	Type	Atoms	Z	Observed(^{o})	Ideal(^{o})
1	A	181	ARG	NE-CZ-NH2	-5.75	117.42	120.30

There are no chirality outliers.

There are no planarity outliers.

5.2 Too-close contacts (i)

In the following table, the Non-H and H(model) columns list the number of non-hydrogen atoms and hydrogen atoms in the chain respectively. The H(added) column lists the number of hydrogen atoms added and optimized by MolProbity. The Clashes column lists the number of clashes within the asymmetric unit, whereas Symm-Clashes lists symmetry-related clashes.

Mol	Chain	Non-H	H(model)	H(added)	Clashes	Symm-Clashes
1	A	1296	0	1292	8	0
1	B	1273	0	1274	8	0
2	A	41	0	0	2	0
2	B	41	0	0	2	0
3	A	150	0	0	3	0
3	B	143	0	0	4	0
All	All	2944	0	2566	17	0

CONFIDENTIAL VALIDATION REPORT

The all-atom clashscore is defined as the number of clashes found per 1000 atoms (including hydrogen atoms). The all-atom clashscore for this structure is 3.

All (17) close contacts within the same asymmetric unit are listed below, sorted by their clash magnitude.

Atom-1	Atom-2	Interatomic distance (Å)	Clash overlap (Å)
2:B:501:KDJ:C45	2:B:501:KDJ:F65	1.62	1.37
1:B:186:LYS:HE3	2:B:501:KDJ:O31	1.89	0.73
1:A:171[B]:CYS:SG	3:B:698:HOH:O	2.55	0.65
1:A:180[A]:GLN:NE2	3:A:601:HOH:O	2.28	0.63
1:B:155:HIS:HE1	1:B:186:LYS:O	1.81	0.62
1:A:155:HIS:HE1	1:A:186:LYS:O	1.85	0.59
1:A:188:ARG:NH2	3:A:607:HOH:O	2.38	0.57
1:A:110:ARG:NH1	3:A:608:HOH:O	2.46	0.49
1:A:92:CYS:SG	1:A:156:ARG:NH2	2.86	0.49
1:B:196:GLU:OE2	3:B:601:HOH:O	2.21	0.44
1:B:110:ARG:HD2	3:B:714:HOH:O	2.19	0.43
1:B:121:GLU:HA	1:B:124:VAL:HG23	2.01	0.43
1:A:120:LYS:HB3	2:A:501:KDJ:O42	2.19	0.42
1:B:90:TYR:CZ	1:B:92:CYS:HB2	2.56	0.41
1:B:197:ILE:HD11	3:B:742:HOH:O	2.19	0.41
1:A:151:LYS:HB2	2:A:501:KDJ:O35	2.21	0.41
1:B:101:LEU:HD23	1:B:101:LEU:HA	1.94	0.40

There are no symmetry-related clashes.

5.3 Torsion angles (i)

5.3.1 Protein backbone (i)

In the following table, the Percentiles column shows the percent Ramachandran outliers of the chain as a percentile score with respect to all X-ray entries followed by that with respect to entries of similar resolution.

The Analysed column shows the number of residues for which the backbone conformation was analysed, and the total number of residues.

Mol	Chain	Analysed	Favoured	Allowed	Outliers	Percentiles	
1	A	158/171 (92%)	151 (96%)	7 (4%)	0	100	100
1	B	153/171 (90%)	146 (95%)	7 (5%)	0	100	100
All	All	311/342 (91%)	297 (96%)	14 (4%)	0	100	100

CONFIDENTIAL VALIDATION REPORT

There are no Ramachandran outliers to report.

5.3.2 Protein sidechains (i)

In the following table, the Percentiles column shows the percent sidechain outliers of the chain as a percentile score with respect to all X-ray entries followed by that with respect to entries of similar resolution.

The Analysed column shows the number of residues for which the sidechain conformation was analysed, and the total number of residues.

Mol	Chain	Analysed	Rotameric	Outliers	Percentiles	
1	A	146/155 (94%)	142 (97%)	4 (3%)	44	31
1	B	143/155 (92%)	140 (98%)	3 (2%)	53	42
All	All	289/310 (93%)	282 (98%)	7 (2%)	47	36

All (7) residues with a non-rotameric sidechain are listed below:

Mol	Chain	Res	Type
1	A	96	TYR
1	A	119	ASN
1	A	120	LYS
1	A	124	VAL
1	B	96	TYR
1	B	104	LEU
1	B	186	LYS

Sometimes sidechains can be flipped to improve hydrogen bonding and reduce clashes. All (4) such sidechains are listed below:

Mol	Chain	Res	Type
1	A	119	ASN
1	A	155	HIS
1	B	155	HIS
1	B	180	GLN

5.3.3 RNA (i)

There are no RNA molecules in this entry.

CONFIDENTIAL VALIDATION REPORT

5.4 Non-standard residues in protein, DNA, RNA chains (i)

There are no non-standard protein/DNA/RNA residues in this entry.

5.5 Carbohydrates (i)

There are no monosaccharides in this entry.

5.6 Ligand geometry (i)

2 ligands are modelled in this entry.

In the following table, the Counts columns list the number of bonds (or angles) for which Mogul statistics could be retrieved, the number of bonds (or angles) that are observed in the model and the number of bonds (or angles) that are defined in the Chemical Component Dictionary. The Link column lists molecule types, if any, to which the group is linked. The Z score for a bond length (or angle) is the number of standard deviations the observed value is removed from the expected value. A bond length (or angle) with $|Z| > 2$ is considered an outlier worth inspection. RMSZ is the root-mean-square of all Z scores of the bond lengths (or angles).

Mol	Type	Chain	Res	Link	Bond lengths			Bond angles		
					Counts	RMSZ	$\#\|Z\| > 2$	Counts	RMSZ	$\#\|Z\| > 2$
2	KDJ	A	501	-	38,41,41	2.05	9 (23%)	55,69,69	1.62	9 (16%)
2	KDJ	B	501	-	38,41,41	1.87	7 (18%)	55,69,69	1.73	10 (18%)

In the following table, the Chirals column lists the number of chiral outliers, the number of chiral centers analysed, the number of these observed in the model and the number defined in the Chemical Component Dictionary. Similar counts are reported in the Torsion and Rings columns. '-' means no outliers of that kind were identified.

Mol	Type	Chain	Res	Link	Chirals	Torsions	Rings
2	KDJ	A	501	-	-	4/39/68/68	0/1/1/1
2	KDJ	B	501	-	-	2/39/68/68	0/1/1/1

All (16) bond length outliers are listed below:

Mol	Chain	Res	Type	Atoms	Z	Observed(Å)	Ideal(Å)
2	A	501	KDJ	PB5-O95	-6.77	1.42	1.54
2	B	501	KDJ	PA2-O32	5.42	1.68	1.50
2	B	501	KDJ	PB5-O85	-5.15	1.42	1.50
2	A	501	KDJ	PA2-O32	5.00	1.66	1.50
2	A	501	KDJ	PB5-O75	-4.09	1.47	1.54
2	B	501	KDJ	PB5-O95	-4.04	1.47	1.54

Continued on next page...

CONFIDENTIAL VALIDATION REPORT

Continued from previous page...

Mol	Chain	Res	Type	Atoms	Z	Observed(Å)	Ideal(Å)
2	A	501	KDJ	PB5-O85	-3.74	1.44	1.50
2	A	501	KDJ	C45-C25	-3.74	1.51	1.55
2	A	501	KDJ	PA4-O14	-3.05	1.53	1.59
2	B	501	KDJ	PA4-O14	-2.73	1.54	1.59
2	B	501	KDJ	PB5-O75	-2.32	1.50	1.54
2	B	501	KDJ	C1-C6	2.09	1.56	1.52
2	A	501	KDJ	PA6-O26	-2.06	1.46	1.54
2	B	501	KDJ	PA2-O12	2.04	1.63	1.59
2	A	501	KDJ	PA4-O34	-2.01	1.47	1.54
2	A	501	KDJ	PA4-O44	-2.00	1.47	1.54

All (19) bond angle outliers are listed below:

Mol	Chain	Res	Type	Atoms	Z	Observed(^{o})	Ideal(^{o})
2	B	501	KDJ	F65-C45-F55	6.76	115.27	106.73
2	A	501	KDJ	F65-C45-F55	6.53	114.98	106.73
2	B	501	KDJ	O14-C4-C5	-3.87	102.23	108.38
2	A	501	KDJ	O95-PB5-O75	3.43	117.67	107.99
2	B	501	KDJ	C4-C5-N15	-3.11	105.75	110.91
2	B	501	KDJ	O11-PA1-O41	-3.02	97.72	109.39
2	A	501	KDJ	O16-C6-C5	-2.83	103.87	108.38
2	B	501	KDJ	O11-C1-C6	2.72	115.10	108.69
2	B	501	KDJ	O22-PA2-O42	2.64	117.72	107.64
2	B	501	KDJ	O44-PA4-O34	2.50	117.18	107.64
2	B	501	KDJ	O16-C6-C5	-2.37	104.60	108.38
2	A	501	KDJ	O75-PB5-O85	2.36	118.36	113.06
2	A	501	KDJ	O23-PA3-O43	2.34	116.56	107.64
2	A	501	KDJ	O22-PA2-O42	2.32	116.51	107.64
2	A	501	KDJ	O44-PA4-O34	2.19	116.02	107.64
2	A	501	KDJ	F65-C45-C25	-2.09	104.87	108.80
2	B	501	KDJ	O13-PA3-O33	-2.07	101.40	109.39
2	B	501	KDJ	O16-PA6-O46	-2.06	101.43	109.39
2	A	501	KDJ	C4-C5-N15	-2.00	107.58	110.91

There are no chirality outliers.

All (6) torsion outliers are listed below:

Mol	Chain	Res	Type	Atoms
2	A	501	KDJ	C6-O16-PA6-O46
2	B	501	KDJ	C6-O16-PA6-O46
2	A	501	KDJ	C4-O14-PA4-O44
2	A	501	KDJ	C4-O14-PA4-O34

Continued on next page...

CONFIDENTIAL VALIDATION REPORT

Continued from previous page...

Mol	Chain	Res	Type	Atoms
2	A	501	KDJ	C6-O16-PA6-O26
2	B	501	KDJ	C4-O14-PA4-O44

There are no ring outliers.

2 monomers are involved in 4 short contacts:

Mol	Chain	Res	Type	Clashes	Symm-Clashes
2	A	501	KDJ	2	0
2	B	501	KDJ	2	0

The following is a two-dimensional graphical depiction of Mogul quality analysis of bond lengths, bond angles, torsion angles, and ring geometry for all instances of the Ligand of Interest. In addition, ligands with molecular weight > 250 and outliers as shown on the validation Tables will also be included. For torsion angles, if less then 5% of the Mogul distribution of torsion angles is within 10 degrees of the torsion angle in question, then that torsion angle is considered an outlier. Any bond that is central to one or more torsion angles identified as an outlier by Mogul will be highlighted in the graph. For rings, the root-mean-square deviation (RMSD) between the ring in question and similar rings identified by Mogul is calculated over all ring torsion angles. If the average RMSD is greater than 60 degrees and the minimal RMSD between the ring in question and any Mogul-identified rings is also greater than 60 degrees, then that ring is considered an outlier. The outliers are highlighted in purple. The color gray indicates Mogul did not find sufficient equivalents in the CSD to analyse the geometry.

CONFIDENTIAL VALIDATION REPORT

Ligand KDJ A 501

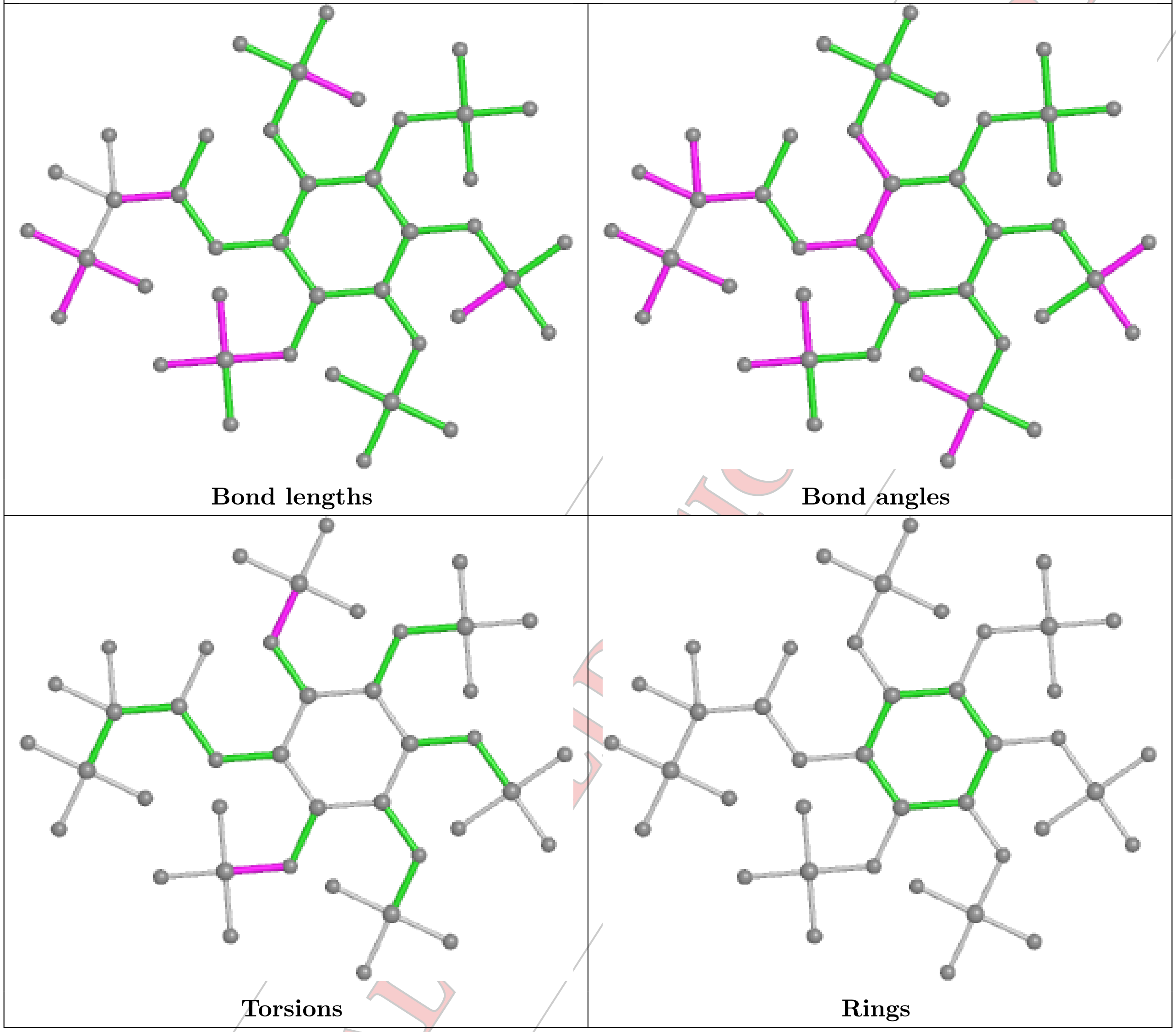

CONFIDENTIAL

Ligand KDJ B 501

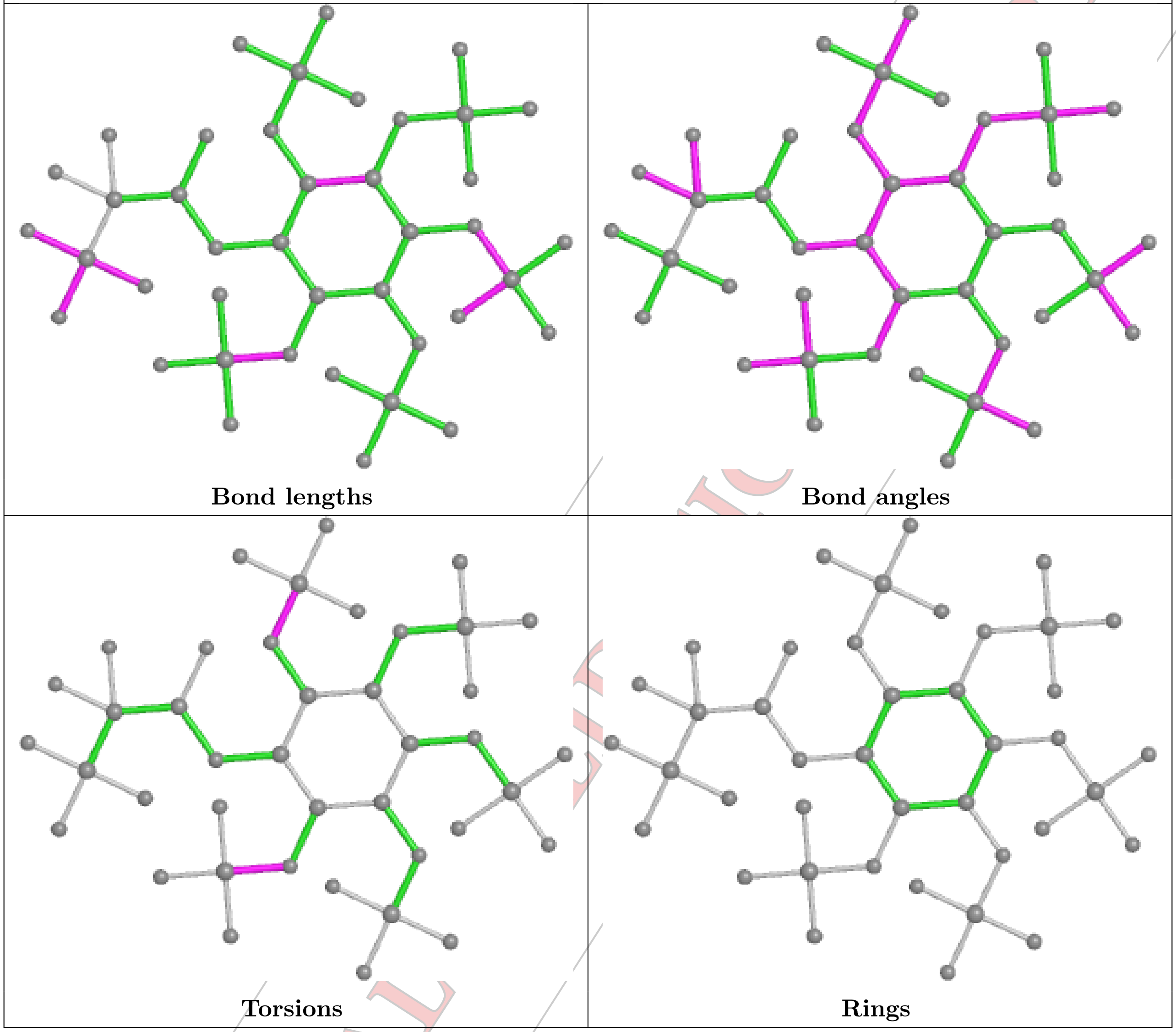

5.7 Other polymers (i)

There are no such residues in this entry.

5.8 Polymer linkage issues (i)

There are no chain breaks in this entry.

CONFIDENTIAL

6 Fit of model and data (i)

6.1 Protein, DNA and RNA chains (i)

In the following table, the column labelled '#RSRZ> 2' contains the number (and percentage) of RSRZ outliers, followed by percent RSRZ outliers for the chain as percentile scores relative to all X-ray entries and entries of similar resolution. The OWAB column contains the minimum, median, 95th percentile and maximum values of the occupancy-weighted average B-factor per residue. The column labelled 'Q< 0.9' lists the number of (and percentage) of residues with an average occupancy less than 0.9.

Mol	Chain	Analysed	<RSRZ>	#RSRZ>2			OWAB(Å^2)	Q<0.9
1	A	155/171 (90%)	-0.19	11 (7%)	16	12	11, 22, 70, 107	0
1	B	152/171 (88%)	-0.07	12 (7%)	12	9	12, 24, 76, 108	0
All	All	307/342 (89%)	-0.13	23 (7%)	14	11	11, 23, 72, 108	0

All (23) RSRZ outliers are listed below:

Mol	Chain	Res	Type	RSRZ
1	B	123	PHE	10.1
1	B	204	SER	9.2
1	A	124	VAL	6.0
1	B	50	GLU	5.9
1	A	118	GLY	5.7
1	B	124	VAL	5.6
1	B	122	PRO	5.3
1	A	119	ASN	5.2
1	A	50	GLU	4.5
1	A	123	PHE	4.4
1	A	204[A]	SER	4.3
1	B	202	SER	3.7
1	B	201	SER	3.5
1	B	125	ASN	3.4
1	B	121	GLU	3.0
1	B	51	LEU	2.8
1	A	122	PRO	2.8
1	A	125	ASN	2.8
1	B	117	GLU	2.8
1	A	120	LYS	2.7
1	A	117	GLU	2.6
1	A	121	GLU	2.2
1	B	199	ASP	2.1

CONFIDENTIAL VALIDATION REPORT

6.2 Non-standard residues in protein, DNA, RNA chains (i)

There are no non-standard protein/DNA/RNA residues in this entry.

6.3 Carbohydrates (i)

There are no monosaccharides in this entry.

6.4 Ligands (i)

In the following table, the Atoms column lists the number of modelled atoms in the group and the number defined in the chemical component dictionary. The B-factors column lists the minimum, median, 95th percentile and maximum values of B factors of atoms in the group. The column labelled 'Q< 0.9' lists the number of atoms with occupancy less than 0.9.

Mol	Type	Chain	Res	Atoms	RSCC	RSR	B-factors(Å^2)	Q<0.9
2	KDJ	B	501	41/41	0.95	0.14	26,56,103,111	0
2	KDJ	A	501	41/41	0.96	0.11	19,46,87,114	0

The following is a graphical depiction of the model fit to experimental electron density of all instances of the Ligand of Interest. In addition, ligands with molecular weight > 250 and outliers as shown on the geometry validation Tables will also be included. Each fit is shown from different orientation to approximate a three-dimensional view.

CONFIDENTIAL VALIDATION REPORT

Electron density around KDJ B 501:

2mF$_o$-DF$_c$ (at 0.7 rmsd) in gray
mF$_o$-DF$_c$ (at 3 rmsd) in purple (negative)
and green (positive)

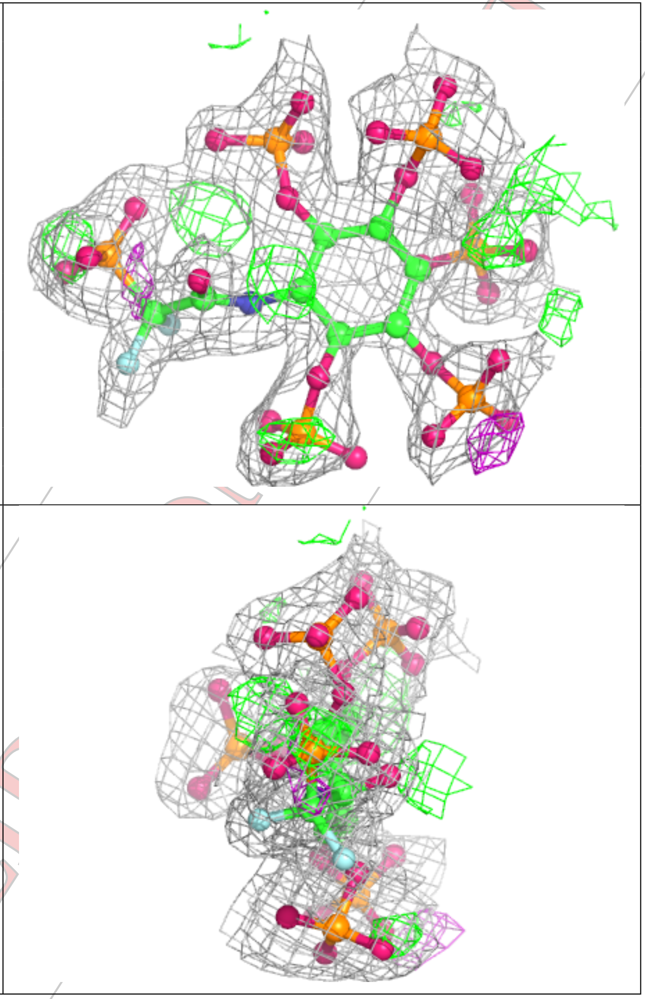

CONFIDENTIAL VALIDATION REPORT

Electron density around KDJ A 501:

$2mF_o$-DF_c (at 0.7 rmsd) in gray
mF_o-DF_c (at 3 rmsd) in purple (negative)
and green (positive)

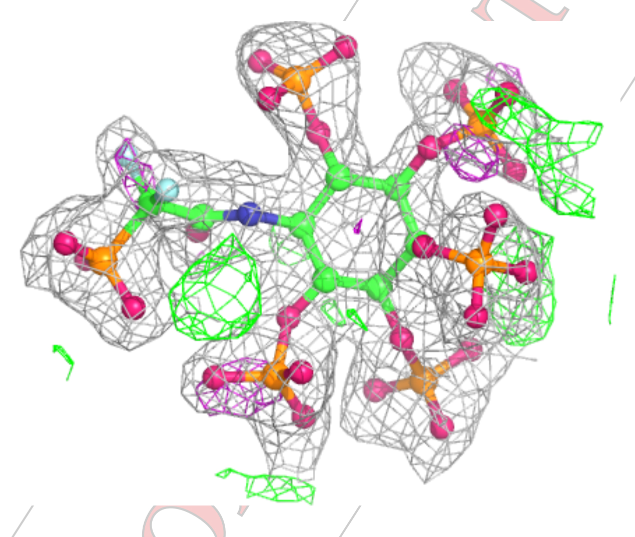

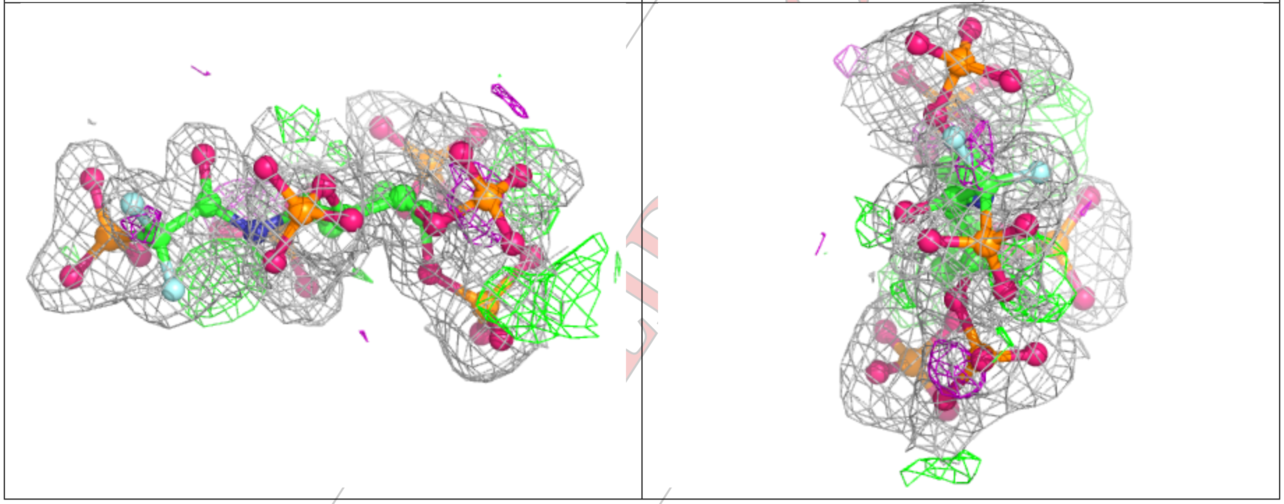

6.5 Other polymers (i)

There are no such residues in this entry.

CONFIDENTIAL
